# A multiscale structure enabled strong, ultra-tough and sustainable polyurethane adhesives

**DOI:** 10.1038/s41467-026-72658-4

**Published:** 2026-05-06

**Authors:** Yubing Fu, Xinyu Chen, Liwei Lu, Wenwei Yang, Siyu Gan, Xueling Yan, Lan Liu

**Affiliations:** School of Materials Science and Engineering, Key Lab of Guangdong Province for High Property and Functional Macromolecular Materials, South China University of Technology, Guangzhou, PR China

**Keywords:** Mechanical properties, Polymers, Organic molecules in materials science

## Abstract

Developing sustainable adhesives with simultaneous high adhesive strength and excellent work of debonding remains challenging. Here, strong, ultra-tough, environmentally reliable, and recyclable polyurethane adhesives are reported, utilizing a multiscale engineering approach. The multiscale structure employs a top-down design philosophy to merge the advantages of both thermoplastic and thermosetting systems, integrating multi-level organizations including macroscale dual-length dynamic crosslinked networks, mesoscale initial and fibrillar crystals, nanoscale hard phase domains, and molecular scale weak hydrogen bonds. The multiscale structure exhibits the following features: multi-level organizations and surface-anchored high energy phases significantly enhance the bulk cohesion and interfacial adhesion, respectively. And hydrophobic organizations provide barrier mechanisms, while dynamic crosslinking enables network reconfiguration. As a result, the developed adhesive exhibits both high adhesive strength (21.24 MPa) and ultrahigh work of debonding (35,731 N m^-1^), coupled with excellent physical and chemical recyclability and environmental reliability (high humidity, water, organic solvents, acids and alkalis, high and ultralow temperatures, aging). This work highlights how multiscale structure enabled by dual-length crosslinked networks simultaneously optimizes bulk cohesion and interfacial adhesion, offering a perspective for designing strong and tough adhesives.

## Abstract (web summary)

Developing sustainable adhesives with simultaneous high adhesive strength and excellent work of debonding remains challenging. Here, the authors report a strong, ultra-tough, and recyclable polyurethane adhesives using a multiscale engineering approach.

## Introduction

Adhesives are critical solutions in daily life and industrial manufacturing, utilized for assembling diverse structural components and parts^1^. Among them, polyurethane (PU) adhesives dominate the field due to their exceptional flexibility, wear resistance, and compatibility with various substrates, finding widespread application in construction, automotive, aerospace, and biomedical industries^2^. However, the trade-off between bulk cohesion and interfacial interactions hinders the simultaneous realization of strong adhesion and ductile adhesion on target surfaces^3–5^. Structural loading requires rigid crosslinking to achieve high adhesive strength, often at the expense of ductility (i.e., low work of debonding)^6,7^. Conversely, ductile adhesion demands high chain mobility to dissipate mechanical stress, typically compromising cohesion (i.e., low adhesive strength)^6,7^. This inherent contradiction in structure significantly restricts their application in structural bonding and high-frequency vibration scenarios. Therefore, there is an urgent need to develop a class of PU adhesives that are able to synergistically exhibit high adhesive strength and high work of debonding.

Currently, there are three primary molecular design strategies for developing thermoplastic PU adhesives that simultaneously exhibit high strength and high toughness: 1) Incorporating sacrificial bond networks into molecular chains, such as weak hydrogen bonds, metal-ligand coordination, and electrostatic interactions^8^. These non-covalent interactions can not only enhance interfacial adhesion but also improve the bulk cohesion and energy dissipation capacity of adhesives to some extent^9–12^. 2) Designing bioinspired soft-hard microphase separation structures^13,14^. Typically, the soft phase dissipates mechanical stress for toughening, while the hard phase domains bear external loads and anchor at interfaces to enhance bulk and interfacial strength, with the strength regulated by their size^7^. For example, Wang et al. developed a soft-hard multiphase structure based on furan rings, enabling the as-prepared polyester adhesive to achieve an adhesive strength of 12.10 MPa and a debonding work of 13,225 N m^-1^, exhibiting over 300% improvement^8^. 3) Introducing crystallizable soft segments to construct mesoscale semi-crystalline structures and/or utilize strain-induced crystallization (SIC) mechanism. For instance, Li et al. employed SIC mechanism of soft-segment poly(tetrahydrofuran) to dynamically enhance the bulk cohesion of adhesive, achieving an adhesive strength of 11.37 MPa^6^. Notably, achieving ideal adhesive performance requires addressing the central challenge of simultaneously enhancing bulk cohesion and interfacial adhesion^8,15–17^, whereas the strategies above often involve trade-offs. Recent advances in synergistically integrating the organizations above to construct multiscale hierarchical structures deepen the understanding of mechanical properties and adhesive chemistry^18–20^. For example, Lu et al. designed a supramolecular oligomer incorporating 2-ureido-4-pyrimidinone and adipic acid units^9^, which initially self-assembled into disordered hydrogen-bonding networks and hard phase domains. Subsequent solution shear-induced alignment further reconfigured them into oriented multiscale structures, including nanoaggregates, nanocrystals, and nanofibrils, thereby overcoming the intrinsic performance limitations of supramolecular oligomers. The resulting adhesive exhibits remarkable adhesive strength (30.60 MPa) and work of debonding (23,600 N m^-1^). Despite these significant advances, linear polymer-based adhesives still exhibit inherent limitations in applications such as structural bonding, particularly in terms of creep resistance, thermal stability, and polar solvent resistance. In contrast, these properties are intrinsic advantages of thermosetting PU systems. However, the dense crosslinked network restricts the polymer chain mobility and rearrangement, coupled with low molecular weight, rendering those strategies effective in thermoplastic systems less effective here. Furthermore, their irreversible curing precludes reuse and recycling, fundamentally constraining sustainable applications necessitating reversible adhesion^9,21^. Therefore, overcoming the thermodynamic and kinetic barriers that impede the integration of those organizations into crosslinked networks remains a critical challenge for developing strong, tough, and sustainable thermosetting PU adhesives.

Here, we propose a multiscale engineering approach that integrates macroscale, mesoscale, nanoscale, and molecular scale features into the PU adhesives. This fundamentally differs from previously reported multiscale structure, such as the oriented one formed in supramolecular oligomer systems through external solution shear fields^9^. Specifically, by constructing a macroscale dual-length dynamic crosslinked network that combines long and short crosslinking chain segments, we strategically merge the advantages of thermoplastic and thermosetting systems: short-chain crosslinking provides high crosslink density, thereby enhancing bulk cohesion, creep resistance, and thermal stability; long-chain crosslinking integrates multi-level organizations that are typically achievable in linear systems with sufficient molecular chain lengths, including molecular scale weak hydrogen bonds, nanoscale hard phase domains, and mesoscale initial and fibrillar crystals, thereby achieving enhanced strength and toughness. This innovative strategy breaks through the inherent limitations of traditional linear and crosslinked networks, simultaneously achieving high bulk cohesion of the adhesives and strong adhesion at the adhesive-substrate interface. As a result, the developed PU adhesive exhibits both high adhesive strength (21.24 MPa) and ultrahigh work of debonding (35,731 N m^-1^). Moreover, the dynamic crosslinked network and multi-level organizations respectively endow the adhesives with efficient chemical/physical recyclability and robust environmental reliability (high humidity, water, acids/alkalis, high/ultralow temperature, organic solvents, and aging). This research underscores the potential of multiscale structural design based on the dual-length dynamic crosslinked network in developing adhesives with high strength and toughness, broadening the application in demanding environments, such as automotive and aerospace.

## Results

### Molecular design and materials characterization

**Fig. 1:**
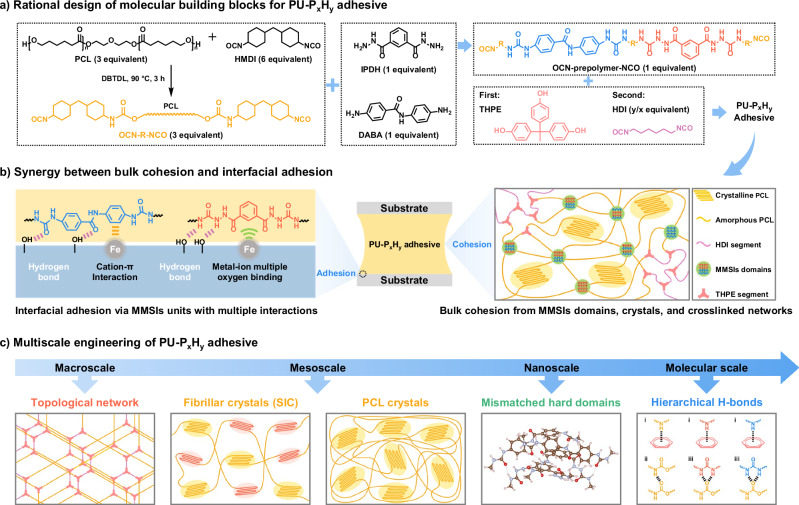
Molecular design and multiscale structure of PU-P_x_H_y_ adhesives. **a** Chemical structure and synthesis of PU-P_x_H_y_ adhesives. **b** Multiscale structure via dual-length crosslinked network for enhanced bulk cohesion and interfacial adhesion, thereby endowing the PU-P_x_H_y_ adhesives with high adhesive strength and high work of debonding. **c** Multiscale engineering of PU-P_x_H_y_ adhesives.

For the molecular design of PU adhesive (Fig. 1a, Supplementary Fig. 1, and Supplementary Table 1), we first developed a dual-length dynamic crosslinked network. Sterically unhindered short-chain hexamethylene diisocyanate (HDI) was employed to provide high crosslink density, thereby enhancing the bulk cohesion. 1,1,1-tris(4-hydroxyphenyl)ethane (THPE) was utilized to form dynamic phenol-carbamate, achieving thermal stimulated dynamic exchange of topological networks. The crosslinked long chain was composed of crystallizable soft segment polycaprolactone diol (PCL), rigid dicyclohexylmethane 4,4’-diisocyanate (HMDI) capable of promoting non-crystalline microphase separation, and mismatched supramolecular interactions (MMSIs) units 4,4’-diaminobenzanilide (DABA) and isophthalic dihydrazide (IPDH), integrating mesoscale, nanoscale, and molecular scale strengthening and toughening modules. This design effectively merges the advantages of linear and crosslinked systems, creating a multiscale structure that simultaneously achieves high bulk cohesion and strong interfacial adhesion in the PU adhesive (Fig. 1b). This adhesive series is denoted as PU-P_x_H_y_, where x/y represents the molar ratio of crosslinked long chain to crosslinked short chain.

These multi-level organizations function hierarchically and synergistically (Fig. 1c). Molecular scale hierarchical weak hydrogen bonds provide effective energy dissipation; MMSIs units form nanoscale hard phase domains for mechanical enhancement, concomitantly dissipating energy via an ongoing dynamic dissociation-reconstruction process. Additionally, multiple interfacial interactions are established with the substrates; Mesoscale initial and fibrillar crystals significantly enhance the bulk cohesion of the PU-P_x_H_y_ adhesives. Among them, high binding energy MMSIs hard domains can prevent the stretched soft segment PCL from relaxing under large strain, thereby facilitating strain-induced crystallization; The macroscale dual-length crosslinked network maintains the skeletal stability and ensures the normal operation of multi-level organizations.

The resultant PU-P_x_H_y_ adhesives were comprehensively characterized, taking PU-P_1_H_3_ as an example. The proton nuclear magnetic resonance (^1^H NMR) spectra are shown in Supplementary Figs. 2–5. After the reaction, the terminal hydroxyl proton peak of PCL (peak a, 4.32 ppm) disappeared, indicating successful prepolymerization. Meanwhile, the primary amine proton peak of IPDH and DABA both shifted downfield, moving from 4.54 to 10.22 ppm and from 4.83 to 8.06 ppm, respectively. This revealed the deshielding effect caused by ureido groups and successful chain extension. According to gel permeation chromatography (GPC) analysis, the number-average molecular weight (*M*_n_) of the obtained prepolymer (i.e., long chain used for crosslinking) was 13.3 kDa, aligning with expectations. Fourier transform infrared (FTIR) spectroscopy was further employed to verify the group reactions (Supplementary Fig. 6). Compared with PCL, PU-P_1_H_3_ exhibited aromatic skeletal vibrations (1600–1460 cm^-1^) and the amide I band (1656 cm^-1^), confirming the successful chain extension of MMSIs units and crosslinking reactions of THPE. The amino peak (3397 cm^-1^) of PU-P_1_H_3_ was between that of IPDH and DABA, and exhibited a wide distribution. This indicates the presence of multiple binding forms of MMSIs units in the polymer network. The carbonyl peak of PU-P_1_H_3_ (1780–1630 cm^-1^) was the strongest due to its highest content. Deconvolution analysis of it (Supplementary Fig. 7) revealed seven subpeaks, assigned to free ester carbonyls (1755 cm^-1^), H-bonded ordered ester carbonyls (1733 cm^-1^), free urethane carbonyls (1743 cm^-1^), H-bonded ordered urethane carbonyls (1714 cm^-1^), free urea carbonyls (1694 cm^-1^), H-bonded ordered urea carbonyls (1632 cm^-1^), and free amide carbonyls (1656 cm^-1^), which were consistent with the molecular design. Additionally, there was no hydroxyl peak (3230 cm^-1^) of THPE or isocyanate peak (about 2260 cm^-1^) in PU-P_1_H_3_, further proving the successful chain extension and crosslinking reaction. The fitting of N-C = O (400.75 eV) from the X-ray photoelectron (XPS) N 1*s* spectrum of PU-P_1_H_3_ (Supplementary Fig. 8) also supported it. The above indicates that the PU-P_x_H_y_ adhesives were successfully synthesized.

The multiscale structure directly influences the thermal properties of PU-P_x_H_y_ adhesives (Supplementary Fig. 9, 10, and Supplementary Table 2). Specifically, as the proportion of crosslinked short chains (y) in the topological network increased, the crosslink density of the adhesive significantly rose, accompanied by a gradual increase in the glass transition temperature (*T*_g_) from −13.4 °C (PU-P_4_H_0_) to −1.7 °C (PU-P_1_H_3_). The overall low *T*_g_ is expected to confer enhanced interfacial wettability and adhesion to the PU-P_x_H_y_ adhesives. Meanwhile, increased crosslink density restricted the motion and rearrangement of crosslinked long chains, leading to a gradual decrease in initial crystallinity. Furthermore, as the proportion of crosslinked long chains (x) in the topological network increased, the content of aromatic MMSIs units in the adhesive rose, enhancing thermal stability. As x increased from 1 to 4, the 5% degradation temperature (*T*_d, 5%_) of PU-P_x_H_y_ adhesives rose from 294.5 °C to 301.7 °C, while the carbon residue at 600 °C increased from 3.53 to 7.07%. Overall, the PU-P_x_H_y_ adhesives exhibit high thermal stability, primarily attributed to the dense hydrogen bonds formed by MMSIs units and the extensive π-conjugated structure of benzene rings.

### Mechanical properties of PU-P_x_H_y_

**Fig. 2:**
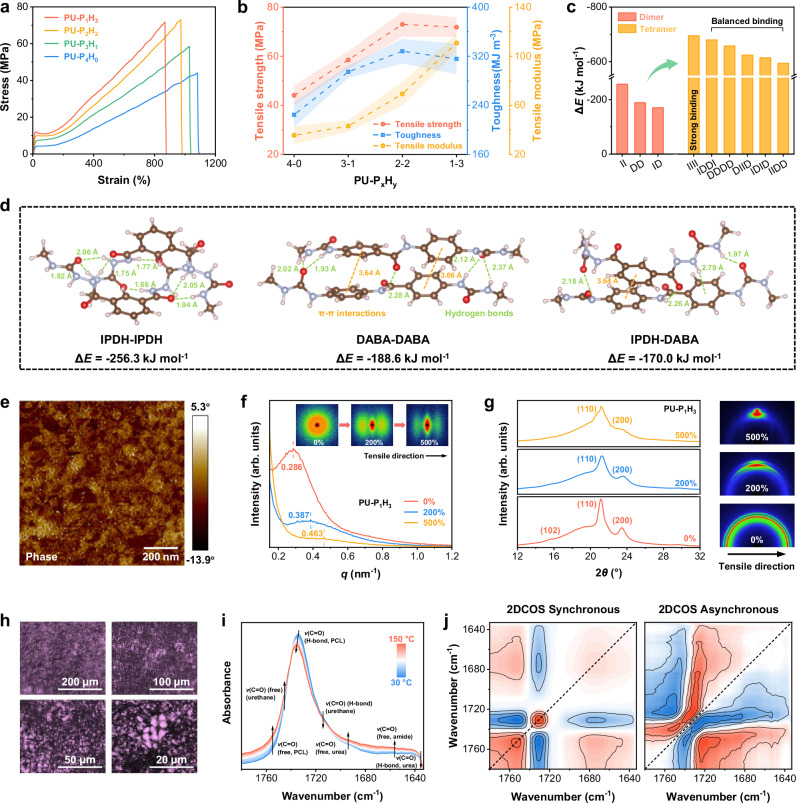
Mechanical properties of PU-P_x_H_y_ and their potential origin. **a** Stress-strain curves of PU-P_x_H_y_. **b** Calculated tensile strength, tensile modulus, and toughness of PU-P_x_H_y_. The sample size (*n*) was 5 for each data point. Error bars denote mean ± standard deviations (SD). **c** Binding energies of dimers and tetramers of MMSIs unit IPDH(I) and/or DABA(D). **d** Three binding modes of MMSIs units dimers: IPDH-IPDH (II), DABA-DABA (DD), and IPDH-DABA (ID). **e** AFM phase diagram of PU-P_1_H_3_. **f** 1D SAXS profiles and 2D SAXS patterns of PU-P_1_H_3_ stretched in situ to different strains. **g** 1D WAXS profiles and 2D WAXS patterns of PU-P_1_H_3_ stretched in situ to different strains. **h** POM images of PU-P_1_H_3_. **i** Variable-temperature FTIR spectra of PU-P_1_H_3_ from 30 °C to 150 °C, with 10 °C increments. **j** 2DCOS synchronous and asynchronous spectra converted from (**i**). Among them, red represents positive intensity, while blue represents negative intensity.

The mechanical properties of PU-P_x_H_y_ determine its load-bearing capacity and ability to dissipate mechanical stress, which is crucial for achieving robust bonding. Figure 2a presents the stress-strain curves of PU-P_x_H_y_. As the proportion of crosslinked short chains (y) increased, the crosslink density of the system significantly rose, leading to systematic changes in mechanical properties: Tensile modulus monotonically increased, ductility gradually decreased, while tensile strength and toughness exhibited a trend of first increasing and then decreasing (Fig. 2b, and Supplementary Table 3). The decrease in tensile strength may be due to the inhibitory effect of gradually dense crosslinked networks on multi-level organizations, including hierarchical weak hydrogen bonds, MMSIs hard phase domains, and PCL and fibrillar crystals. Additionally, the strain hardening and SIC processes of PU-P_x_H_y_ gradually advanced. Notably, PU-P_1_H_3_ began to exhibit plastic yielding behavior, indicating the elastic-to-plastic transition of PU-P_x_H_y_. More importantly, the PU-P_x_H_y_ system demonstrated a surprising synergy between strength and toughness, making it possible to achieve ideal bonding. Based on the influence of modulus, strength, and toughness on adhesion performance of adhesives, PU-P_1_H_3_ exhibits the most comprehensive properties, with a tensile modulus of 110.84 MPa, a strength up to 71.83 MPa, an elongation at break of 868.1%, and remarkable toughness of 315.73 MJ m^-3^. To our knowledge, the synergistic high strength and toughness of PU-P_1_H_3_ surpass most reported thermoset elastomers (Supplementary Table 4)^22–30^.

The contribution of the designed multiscale structure (Fig. 1c) to the mechanical properties of PU-P_x_H_y_ and its underlying mechanism were thoroughly investigated. At the molecular scale, deconvolution analysis of the FTIR carbonyl peak for PU-P_1_H_3_ (Supplementary Fig. 7) provided direct evidence for hierarchical weak hydrogen bonding, including H-bonded ester group and H-bonded ureido group. Additionally, there may be weak N-H···π hydrogen bonds between secondary amines and benzene rings in the system. These hierarchical weak hydrogen bonds provide effective mechanical energy dissipation for PU-P_x_H_y_ through continuous dissociation and reformation. At the nanoscale level, we comprehensively investigated the strengthening and toughening mechanisms of MMSIs units (i.e., IPDH and DABA) using density functional theory (DFT) calculations, atomic force microscopy (AFM), and small-angle X-ray scattering (SAXS). Based on the molecular structure of the PU-P_x_H_y_, supramolecular interactions are evidently concentrated between the rigid segments IPDH and DABA. Therefore, DFT calculations were first employed to investigate their binding energies, revealing their supramolecular interactions (Fig. 2c, d, and Supplementary Fig. 11). Results indicate that IPDH and/or DABA interacted strongly through hydrogen bonds and π-π interactions, and can spontaneously form aggregates. For instance, in the PU-P_x_H_y_ system, equimolar IPDH and DABA segments can form dimers in three distinct combinations: IPDH-IPDH (denoted as II), DABA-DABA (DD), and IPDH-DABA (ID), with binding energies of −256.3, −188.6, and −170.0 kJ mol^-1^ respectively (Fig. 2d). Moreover, they tend to form larger aggregates (e.g., tetramers) with lower binding energies, providing a thermodynamic driving force for microphase separation. Notably, the number of hydrogen bond donors/acceptors and the number of benzene rings participating in π-π stacking are matched in II and DD, whereas the ID is mismatched. Therefore, the interaction between segments IPDH and DABA is defined as mismatched supramolecular interactions (MMSIs). As shown in Fig. 2c and Supplementary Fig. 11, the tetramer containing equimolar IPDH and DABA segments exhibited six aggregation combinations. Among these, IIII demonstrated strong binding, while the remaining five combinations (IDDI, DDDD, DIID, IDID, IIDD) exhibited balanced binding with progressively decreasing stability. This enhances the dynamic reversibility of MMSIs aggregates, facilitating easier dissociation upon external stimulation to dissipate mechanical energy. Actually, unrestricted enhancement and enrichment of supramolecular interactions do not equate to increased material toughness. A balance must be struck between the strengthening property of ultra-strong binding and the energy-dissipating property of dynamic dissociation-reconstitution, particularly when binding energies exceed those of covalent bonds. Therefore, the advantage of MMSIs aggregates lies in their ability to incorporate both matched strong binding for strengthening and mismatched balanced binding for toughening, thereby achieving synergistic strength-toughness enhancement. Furthermore, the AFM phase image of PU-P_1_H_3_ (Fig. 2e) provided visual evidence for the formation of dynamic nano hard phase domains by MMSIs units. Subsequently, the microphase structure evolution of PU-P_x_H_y_ during stretching was characterized by small-angle X-ray scattering (SAXS), taking PU-P_1_H_3_ as an example (Fig. 2f). With increasing strain, the scattering pattern gradually transformed from an isotropic diffuse ring to an elliptical ring, while the scattering signal perpendicular to the stretching direction progressively weakened. The process was accompanied by a gradual decrease in the average spacing of the microphase domains, specifically 22.0 nm (0%), 16.2 nm (200%), and 13.6 nm (500%). This indicates that the initial state of MSSIs hard domains is uniformly distributed, achieving nanoscale reinforcement. During stretching, these domains bear external forces and preferentially align along the stretching direction, leading to disruption of the structural periodicity perpendicular to the stretching direction. The continuous disintegration and reformation of MSSIs hard phase domains efficiently dissipates energy, achieving toughening. At the mesoscale, we employed wide-angle X-ray scattering (WAXS) to investigate the crystallization and its mechanical response evolution of PU-P_x_H_y_ during stretching, using PU-P_1_H_3_ as an example. As shown in Fig. 2g, the initial WAXS profile displayed characteristic diffraction peaks of PCL crystal at 2θ = 21.3° (110), 23.5° (200), and 15.6° (102)^31^. The scattering pattern exhibited a symmetrical ring structure, indicating that PU-P_1_H_3_ exists in a semi-crystalline, isotropic state. The spherulites and their thermal disintegration process were also directly observed through polarized optical microscopy (POM) images of PU-P_1_H_3_ (Fig. 2h, and Supplementary Fig. 12). This phenomenon, difficult to achieve in conventional crosslinked systems, is attributed to the designed macroscale dual-length crosslinked topological network. As strain increased, the scattering signal along the stretching direction gradually weakened, while the equatorial direction evolved into a figure-of-eight pattern. This is a hallmark of highly oriented crystals and even strain-induced crystallization (i.e., fibrillar crystals). Meanwhile, optical images of PU-P_1_H_3_ over multiple tensile cycles (600% strain) demonstrated that the SIC process is reversible (Supplementary Fig. 13). Furthermore, fitting results of 1D WAXS profiles (SupplementaryTable 5) reveal that crystallinity of PU-P_x_H_y_ first decreased and then increased during stretching. The above indicates that PCL forms initial crystalline structures, enhancing the modulus and achieving reinforcement in PU-P_x_H_y_. During stretching, the PU-P_x_H_y_ system successively undergoes lamellae tilting and sliding, lamellae disintegration, crystallite orientation, and strain-induced crystallization, dissipating significant energy. Overall, the multiscale structure provides diverse hierarchical pathways for strengthening and toughening, which are crucial for improving the mechanical properties of PU-P_x_H_y_.

The response sequence of various carbonyl groups in PU-P_x_H_y_ was investigated via variable-temperature FTIR spectroscopy to reflect the hierarchical energy dissipation order of weak hydrogen bonds, MMSIs hard phase domains, and PCL crystals within the system during stretching. As shown in Fig. 2i, with temperature increasing from 30 °C to 120 °C, the absorbance of all the H-bonded ordered carbonyl groups in PU-P_1_H_3_ decreased, while that of all the corresponding free carbonyl groups gradually increased. The variable-temperature FITR spectra were further converted into two-dimensional correlation spectra (2DCOS, Fig. 2j). According to Noda criterion^32^, considering the signs of cross peaks in both synchronous and asynchronous spectra (Supplementary Table 6-8), the response sequence of different carbonyl groups to heating is ( → denotes earlier than): 1694 → 1656 → 1755 → 1743 cm^-1^, i.e., *v*(C = O) (free urea carbonyls) → *v*(C = O) (free amide carbonyls) → *v*(C = O) (free ester carbonyls) → *v*(C = O) (free urethane carbonyls). This indicates that strong hydrogen bonds (urethane and amide) within the MSSIs hard phase domains responded preferentially over weak hydrogen bonds (ester) in the system. This anomalous phenomenon arose because weak hydrogen bonds like the ester carbonyl are shielded by the PCL crystalline regions. Thus, during PU-P_x_H_y_ stretching, the multiscale structure provides a unique hierarchical energy dissipation mechanism: MMSIs hard phase domains disintegration → crystal fracture → weak hydrogen bonds dissociation.

### Adhesion properties of PU-P_x_H_y_ adhesives

**Fig. 3:**
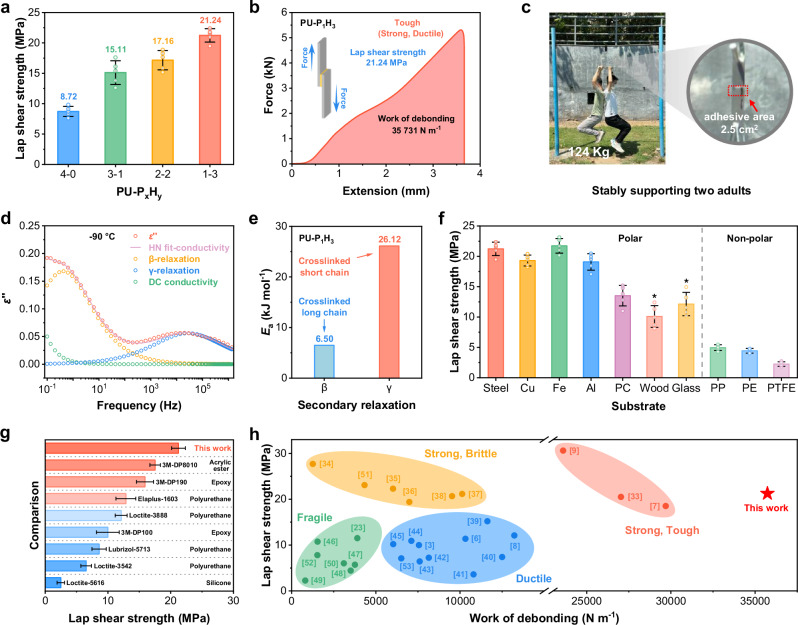
Adhesion properties of PU-P_x_H_y_ adhesives. **a** Lap shear strength of PU-P_x_H_y_ adhesive-bonded stainless steel joints. **b** Force-extension curve from lap shear test of PU-P_1_H_3_ adhesive. **c** Image showing stainless steel joints bonded with PU-P_1_H_3_ adhesive supporting two adults (124 kg). **d** Dielectric loss spectrum and Havriliak-Negami (H-N) fitting of PU-P_1_H_3_ adhesive at −90 °C. **e** Activation energies (*E*_a_) of secondary relaxations (β and γ) for PU-P_1_H_3_ adhesive. **f** Lap shear strength of PU-P_1_H_3_ adhesive bonded on different substrates. Asterisk (*) indicates substrate damage during testing. **g** Comparison of lap shear strength for stainless steel joints bonded with PU-P_1_H_3_ adhesive and various commercial adhesives. **h** Comparison of lap shear strength and work of debonding between PU-P_1_H_3_ adhesive and reported advanced adhesives. Error bars represent mean ± SD (*n* = 5 per data point).

Lap shear tests were employed to evaluate the adhesion properties of PU-P_x_H_y_ adhesives on stainless steel substrates. The adhesive was applied via hot pressing. Specifically, a sandwich-like structure comprising stainless steel sheet-adhesive-stainless steel sheet was clamped, heated at 140 °C for 2 h, and cooled to room temperature to complete the bonding process. Rheological data (Supplementary Fig. 14) indicate that 140 °C lies between the relaxation peak of the MMSIs hard domains physical crosslinked network (130.4 °C) and the solid-liquid transition point (160.3 °C). At this temperature, PU-P_x_H_y_ exhibits sufficient flowability for effective interfacial wetting while retaining sufficient viscoelastic integrity to resist excessive flow. Figure 3a shows the lap shear strength (hereafter referred to as adhesive strength) of PU-P_x_H_y_ adhesive-bonded stainless steel joints. As the proportion of crosslinked short chains (y) in the topological network increased, the adhesive strength rose monotonically, peaking at 21.24 MPa (PU-P_1_H_3_), which meets the requirements of structural bonding. This trend aligns with the tensile modulus trend, driven by significantly enhanced bulk cohesion due to increased crosslink density. Within a certain modulus range, PU-P_1_H_3_ adhesive with higher modulus can more efficiently transfer stress between substrates, limiting deformation and energy accumulation within the adhesive layer. This enables it to withstand greater external loads and exhibit higher adhesive strength. Furthermore, as shown in Fig. 3b and Supplementary Table 9, the debonding work of PU-P_x_H_y_ adhesives exhibited a monotonically increasing trend, jointly influenced by bulk toughness and interfacial interactions, reaching a remarkable peak of 35,731 N m^-1^ (PU-P_1_H_3_). This value ranks among the highest reported for strong and tough adhesives to date, offering potential benefits for applications requiring energy absorption, impact resistance, or overload tolerance. To visually demonstrate the exceptional adhesion properties of PU-P_x_H_y_ adhesives, load test was conducted using stainless steel joints bonded with PU-P_1_H_3_ adhesive. The results showed that it could stably support two adults weighing 124 kg (Fig. 3c), sustain an 8 kg load for 30 days without detectable creep or adhesive failure (Supplementary Fig. 15), and exhibited stabilized creep strain within 30 minutes under 1-4 MPa lap shear stress (Supplementary Fig. 16). The interfacial adhesion between PU-P_x_H_y_ adhesives and substrates primarily relies on multiple interactions between MMSIs units and the substrate surface, such as hydrogen bonding, cation-π interaction, and metal-ion multiple oxygen binding (Fig. 1b). To investigate the mechanism behind this behavior, we employed broadband dielectric spectroscopy (BDS) to study the secondary relaxation of PU-P_1_H_3_ adhesive (Fig. 3d, e, and Supplementary Fig. 17). Results indicate that the dielectric loss spectra of PU-P_1_H_3_ adhesive measured at each temperature below *T*_g_ can be fitted by three Havriliak-Negami (H-N) functions, which resolve into two relaxation curves (β and γ relaxation) and one curve representing the DC conductivity contribution. β relaxation is associated with the local motion of crosslinked long chains, while γ relaxation relates to the local motion of crosslinked short chains. The activation energy (*E*_a_) was further fitted using the Arrhenius equation. The *E*_a_ for β relaxation was 6.50 kJ mol^-1^, significantly lower than that for γ relaxation (26.12 kJ mol^-1^). This inherent and readily activated mobility endows the adhesive functional groups on crosslinked long chains with enhanced conformational adaptability and interfacial diffusion across the entire temperature-time spectrum during bonding, granting PU-P_x_H_y_ adhesives a kinetic advantage for exceptional interfacial adhesion. Besides, relaxations at −90 °C correspond to high frequencies at room temperature. This rapid molecular dynamics facilitates both prompt substrate wetting and efficient formation of strong interfacial interactions through dynamically driven functional group alignment, thereby maximizing intrinsic adhesive strength. Based on established adhesion theory, adhesion properties are determined by bulk cohesion and interfacial interactions^15,16^. The designed multiscale structure endows PU-P_x_H_y_ adhesives with unique characteristics: 1) High bulk cohesion through molecular scale hydrogen bonding, nanoscale MMSIs hard phase domains, and mesoscale PCL and fibrillar crystals; 2) High toughness through a hierarchical energy dissipation mechanism involving MMSIs hard phase domains disintegration - crystal fracture - weak hydrogen bonds dissociation; 3) Strong interfacial adhesion through multiple noncovalent interactions between MMSIs units and the target substrates, including hydrogen bonding, cation-π interactions, and metal-ion multiple oxygen binding. These factors synergistically resolve the trade-off between strength and toughness in PU-P_x_H_y_ adhesives, simultaneously delivering high adhesive strength and ultrahigh work of debonding. Given its comprehensive adhesion properties, PU-P_1_H_3_ adhesive was selected for subsequent investigations.

To investigate the applicability of PU-P_x_H_y_ adhesives to different substrates, multiple substrates bonded with PU-P_1_H_3_ adhesive were subjected to lap shear tests. As shown in Fig. 3f, the adhesive strength of PU-P_1_H_3_ adhesive, when bonded to polar substrates including stainless steel, copper (Cu), iron (Fe), aluminum (Al), polycarbonate (PC), wood, and glass, reached 21.24, 19.31, 21.74, 19.09, 13.52, 10.09, and 12.14 MPa, respectively. Notably, substrate failure occurred for wood and glass due to excessive adhesion, rendering valid results unobtainable. Encouragingly, the PU-P_1_H_3_ adhesive also exhibited promising adhesive strength on non-polar substrates, reaching 4.96 MPa on polypropylene (PP), 4.44 MPa on polyethylene (PE), and 2.25 MPa on polytetrafluoroethylene (PTFE). This demonstrates the broad applicability of PU-P_x_H_y_ adhesives to diverse substrates, including metal and non-metallic materials. Furthermore, we compared the adhesion properties of PU-P_1_H_3_ adhesive against commercial adhesives and those reported in the literature. PU-P_1_H_3_ adhesive exhibited the highest adhesive strength among all tested commercial adhesives (Fig. 3g), including structural adhesives known for their adhesive strength. More importantly, PU-P_1_H_3_ adhesive resolves the strength-toughness trade-off, exhibiting both high adhesive strength and ultrahigh work of debonding (Fig. 3h, and Supplementary Table 10), significantly outperforming the reported state-of-the-art adhesives^3,6–9,23,33–53^.

### Environmental reliability of PU-P_x_H_y_ adhesives

Interestingly, beyond their exceptional combination of high adhesive strength and ultrahigh work of debonding, PU-P_x_H_y_ adhesives demonstrated unanticipated environmental reliability, including excellent resistance to high humidity, water, organic solvent, acids and alkalis, high and ultralow temperatures, and aging, which is a significant advantage over most traditional adhesive systems. This substantially boosts the competitiveness and applicability of PU-P_x_H_y_ adhesives, facilitating their adaptation to progressively intricate service environments.

**Fig. 4:**
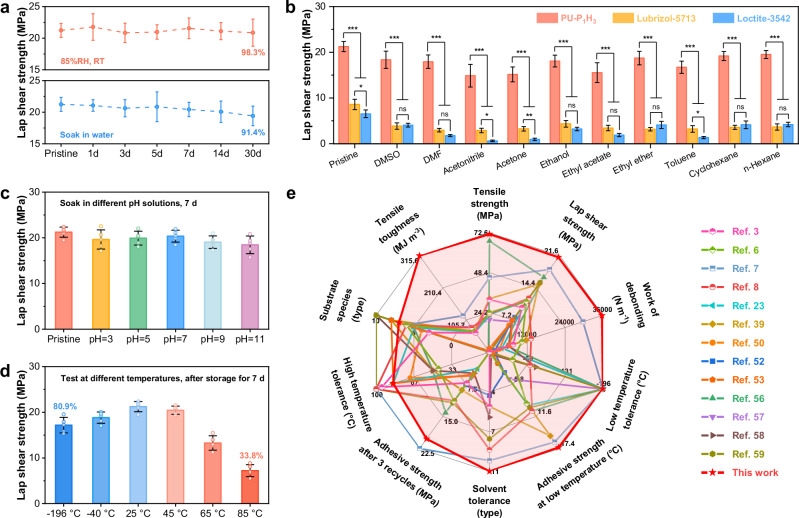
Environmental reliability of PU-P_x_H_y_ adhesives. **a** Lap shear strength of PU-P_1_H_3_ adhesive-bonded stainless steel joints after 30 days of exposure to 85%RH or water immersion. **b** Lap shear strength of stainless steel joints bonded with PU-P_1_H_3_ and commercial PU adhesives (Lubrizol-5713, Loctite-3542) after 24-hour immersion in 10 different organic solvents. **c** Lap shear strength of PU-P_1_H_3_ adhesive-bonded stainless steel joints after 7-day immersion in different pH solutions. **d** Lap shear strength of PU-P_1_H_3_ adhesive-bonded stainless steel joints after 7-day exposure to various temperatures, tested at the exposure temperature. **e** Comprehensive performance comparison of PU-P_1_H_3_ adhesive with advanced adhesives previously reported. Error bars represent mean ± SD (*n* = 5 per data point). Probability (*p*) values were determined by one-way analysis of variance, with significance levels denoted as ^ns^ *p* > 0.05, *p ≤ 0.05, **p < 0.01, and ***p < 0.001.

Due to the attack and penetration of water molecules on the adhesive and the adhesive-substrate interface, prolonged exposure to high humidity or water immersion is a scenario that must be considered in practical adhesive applications. To evaluate this performance, we conducted lap shear tests on PU-P_1_H_3_ adhesive-bonded stainless steel joints, exposed to an 85%RH environment or immersed in water for 30 days. As shown in Fig. 4a, the adhesive strength exhibited consistent stability at 85%RH, ultimately retaining a high value of 20.88 MPa. When immersed in water, only a minor time-dependent reduction was observed, with 91.4% of the initial adhesive strength ultimately preserved. These results fully reveal the moisture and water resistance of PU-P_x_H_y_ adhesives. This excellent performance is primarily attributed to a dual mechanism: the combined barrier effect of hydrophobic PCL segments (including their crystals) and the crosslinked network against water vapor transport within the adhesive bulk, coupled with strong interfacial interactions between MMSIs units and the substrate surface that effectively impede water vapor ingress at the adhesive-substrate interface. Organic solvents or acidic/alkaline solutions represent extreme environments that adhesives occasionally encounter, frequently leading to the bond failure of most traditional adhesives. However, PU-P_x_H_y_ adhesives demonstrated unexpected resistance. As shown in Fig. 4b and Supplementary Fig. 18, the PU-P_1_H_3_ adhesive exhibited tolerance to ten different organic solvents. Specifically, after 24-hour immersion in n-hexane, cyclohexane, toluene, ethyl ether, ethyl acetate, ethanol, acetone, acetonitrile, N,N-dimethylformamide (DMF), or dimethyl sulfoxide (DMSO), the adhesive strength retention rates of PU-P_1_H_3_ adhesive-bonded stainless steel joints reached 91.9%, 90.3%, 78.7%, 88.2%, 73.2%, 85.1%, 71.3%, 70.1%, 84.5%, and 86.5%, respectively. This adhesive strength retention significantly outperformed that of commercial adhesives. For instance, when immersed in DMF, the PU-P_1_H_3_ adhesive exhibited an adhesive strength retention of 86.5%, substantially higher than that of commercial adhesives Lubrizol-5713 (34.5%) and Loctite-3542 (27.1%). After 24-h immersion in organic solvents, stainless steel joints bonded with PU-P_1_H_3_ adhesive could still easily support a 2.5 kg weight in organic solvents (Supplementary Fig. 19). To further investigate acid/alkali resistance, we immersed PU-P_1_H_3_ adhesive-bonded stainless steel joints in acidic/alkaline solutions at different pH values (3, 5, 7, 9, and 11) for 7 days (Fig. 4c). Results indicate that PU-P_1_H_3_ adhesive showed tolerance to all tested acidic/alkaline solutions. Adhesive strength exhibited only minor decreases, with the lowest value still reaching 18.46 MPa (pH = 11), sufficient for practical applications. Notably, the rate of adhesive strength loss was highest in alkaline environments, followed by acidic, and slowest in neutral, a phenomenon caused by the pH-dependent hydrolysis of the soft segment PCL. Overall, PU-P_x_H_y_ adhesives exhibited excellent resistance to organic solvents and acidic/alkaline environments. These resistances stem from the barrier mechanisms provided by the designed multiscale structure, including MMSIs hard phase domains, hydrophobic PCL segments and their crystals, and crosslinked networks. Furthermore, PU-P_x_H_y_ adhesives demonstrate excellent thermal stability, maintaining high adhesive strengths at both high and ultralow temperatures. As shown in Fig. 4d and Supplementary Fig. 20, PU-P_1_H_3_ adhesive-bonded stainless steel joints retained 80.9% of its adhesive strength at −196 °C, without experiencing bond failure due to cryogenic embrittlement. This is attributed to the lubrication effect of bound water stabilized by dense hydrogen bonds^35,54^, the broadened energy dissipation window enabled by hierarchical hydrogen bonds, and the free volume provided by nanoscale microphase separation^55^. When the temperature rose to 85 °C, the adhesive strength decreased to 7.21 MPa due to the thermal disintegration of PCL crystals. Nevertheless, this adhesive strength is sufficient to meet the requirements of most applications at 85 °C. Evaluating the long-term stability of adhesives is critical for predicting their service life and ensuring sustained functional integrity under various environmental conditions. After storage under ambient conditions for 6 months, the adhesive strength of PU-P_1_H_3_ adhesive-bonded joints across various substrates (stainless steel, Cu, Fe, Al, and PC) remained largely consistent with that of initial samples (Supplementary Fig. 21). Furthermore, even after thermal accelerated aging at 100 °C for 14 days, no decrease in adhesive strength was observed (Supplementary Fig. 22). These results highlight the excellent long-term stability of PU-P_x_H_y_ adhesives. Finally, a comprehensive performance comparison of PU-P_1_H_3_ adhesive against advanced adhesives previously reported (Fig. 4e, and Supplementary Table 11) reveals its advantages in tensile strength, tensile toughness, lap shear strength, work of debonding, substrate adaptability, environmental reliability, and recyclability^3,6–8,23,39,50,52,53,56–59^. To the best of our knowledge, PU-P_1_H_3_ adhesive represents one of the most comprehensive adhesives currently available, particularly achieving both high adhesive strength and ultrahigh work of debonding simultaneously.

### Synergistic enhancement of bulk cohesion and interfacial adhesion

The multiscale structure-enhanced bulk cohesion and interfacial adhesion was systematically investigated. As previously illustrated (Fig. 2), the macroscale dual-length dynamic crosslinked network strategically merges the advantages of thermoplastic and thermosetting systems, enabling integration of multiscale features that synergistically endow PU-P_x_H_y_ with high bulk cohesion. The short-chain crosslinking provides high crosslink density, while the long-chain crosslinking integrates multi-level organizations typically achievable in linear systems with sufficient molecular chain lengths, including molecular scale weak hydrogen bonds, nanoscale hard phase domains, and mesoscale initial and fibrillar crystals. These multi-level organizations function hierarchically and synergistically, achieving concurrent enhancement of both strength and toughness. Notably, PU-P_1_H_3_ demonstrated exceptional mechanical properties with tensile modulus, strength, and toughness up to 110.84 MPa, 71.83 MPa, and 315.73 MJ m^-3^, respectively, fully satisfying the bulk requirements for tough adhesives.

**Fig. 5:**
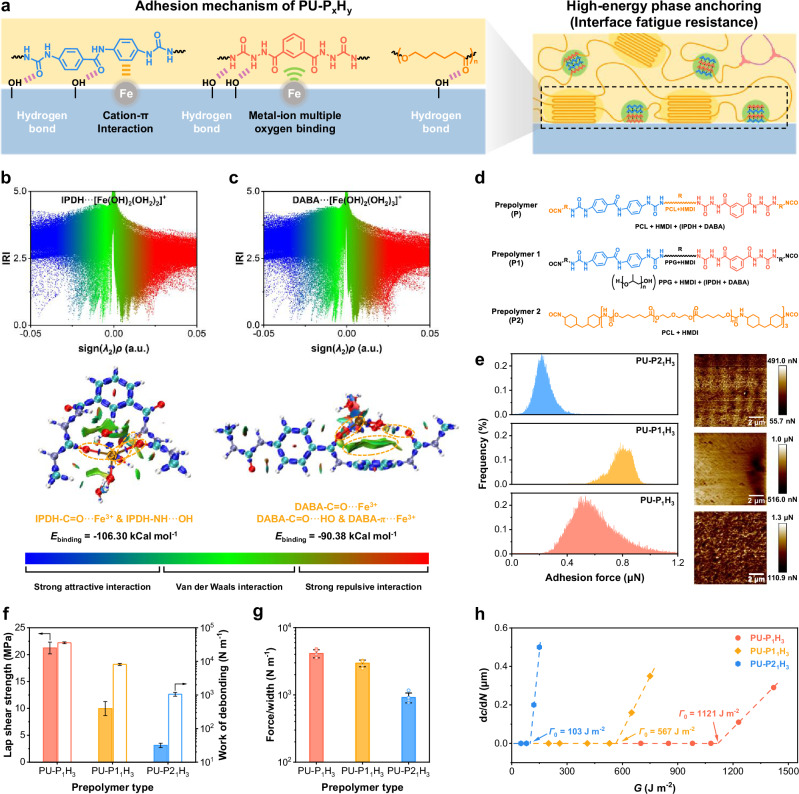
Interfacial characterizations of PU-P_x_H_y_ adhesives. **a** Interfacial interaction and fatigue-resistant interface adhesion mechanism. Plots of IRI versus sign(*λ*_2_)*ρ* and the gradient isosurfaces for the complexes: **b** IPDH···[Fe(OH)_2_(OH_2_)_2_]^+^, **c** DABA···[Fe(OH)_2_(OH_2_)_3_]^+^. **d** Structures of the three prepolymers, imparting distinct multi-scale organizations. **e** Adhesion force distribution maps and **f** comparison of the adhesion properties of PU-P_1_H_3_, PU-P1_1_H_3_, and PU-P2_1_H_3_ adhesives. **g** Interfacial fracture toughness, and **h** interfacial fatigue threshold for PU-P_1_H_3_, PU-P1_1_H_3_, and PU-P2_1_H_3_ on stainless steel substrates. Error bars represent mean ± SD (*n* = 5 per data point).

We further conducted interfacial characterization to investigate the adhesion mechanism, interfacial interactions, surface properties and contributions of multi-level organizations in PU-P_x_H_y_. The potential adhesion mechanism of PU-P_x_H_y_ adhesives are as follows (Fig. 5a): During the hot pressing adhesion process, the MMSIs units with low relaxation time and PCL chain segments synergistically enhance interfacial adhesion through multiple non-covalent interactions, including hydrogen bonding, cation-π interaction, metal-ion multiple oxygen binding. These interactions promote intimate contact between the adhesive and substrate, while partially achieving mechanical interlocks with microscopic interfacial gaps. Consequently, multiscale high energy phases can form in situ at the adhesive-substrate interface, comprising MMSIs hard domains and PCL crystals. They anchor at the interface and effectively hold the interfacial crack tip, thereby elevating the interfacial adhesion and fatigue threshold. DFT calculations were first employed to simulate the adhesive-substrate interfacial adhesion, utilizing mononuclear Fe(III) hydroxo-aqua complexes to characterize the hydroxyl-terminated iron substrate surface (Fig. 5b, c, and Supplementary Fig. 23). Both interaction region indicator (IRI) scatter plots and isosurface maps clearly confirm that IPDH and DABA engage in the aforementioned non-covalent interactions with the substrate. Furthermore, comparative surface spectroscopy before and after adhesion (Supplementary Fig. 24, 25) provides direct chemical evidence for these interfacial interactions. The observed redshifts of *v*(N-H) at 3369.0 cm^-1^ and the benzene ring *v*(C = C) at 1515.8 cm^-1^ in FTIR spectra indicate hydrogen bonding and cation-π interactions at the interface. Raman spectra revealed a redshift of C = O stretching peak (1768.7 cm^-1^) and the emergence of Fe-O peak (515.6 cm^-1^), confirming metal-ion multiple oxygen binding at the interface. To elucidate the respective contributions of MMSIs hard domains and PCL crystals to interfacial adhesion, two additional prepolymers were synthesized for decoupled comparison (Fig. 5d). The soft segment in prepolymer 1 (P1) was substituted with polypropylene glycol (PPG), whereas prepolymer 2 (P2) was synthesized without MMSIs units, in contrast to the original prepolymer (P). Thus, the cured PU-P1_1_H_3_ adhesive contained only MMSIs hard domains, while PU-P2_1_H_3_ retained exclusively PCL crystals. Peak Force Quantitative Nanomechanical Mapping (PF QNM) was then employed to characterize the modulus and adhesion distributions across surface layers. As shown in Supplementary Fig. 26, PU-P_1_H_3_ exhibited higher and more broadly distributed surface modulus compared to PU-P1_1_H_3_ and PU-P2_1_H_3_, suggesting the accumulation of both high energy phases (MMSIs hard domains and PCL crystals) at its surface, achieved through multiscale design. Notably, the mean interfacial adhesion forces for PU-P_1_H_3_, PU-P1_1_H_3_, and PU-P2_1_H_3_ were quantified as 0.53, 0.80, and 0.22 μN, respectively (Fig. 5e), demonstrating that MMSIs hard domains play a dominant role in interfacial adhesion enhancement, while PCL crystals exhibit a weaker but non-negligible contribution. We further conducted lap shear tests (Fig. 5f). The PU-P_1_H_3_ adhesive, which combines high bulk cohesion and strong interfacial interactions, exhibited the highest adhesive strength and work of debonding. Due to the absence of PCL crystals, the PU-P1_1_H_3_ adhesive showed reduced bulk cohesion (Supplementary Fig. 27), leading to decreased adhesive strength (9.96 MPa) and work of debonding (8137 N·m^-1^). As for the PU-P2_1_H_3_ adhesive, the lack of MMSIs units substantially weakened interfacial interactions, resulting in significantly compromised adhesive properties. Besides, although the adhesive strength of PU-P_x_H_y_ correlates strongly with tensile strength trends, indicating bulk cohesion-dominated failure, PU-P_1_H_3_ anomalously exhibited interfacial failure (Supplementary Fig. 28). This deviation stems from two mechanisms: i) Increasing crosslinked short chains (y) proportion enhances bulk cohesion (Fig. 2a) while degrading interfacial interactions through enhanced modulus, reduced wettability, and diminished MMSIs mass fraction. When the ratio of long to short crosslinked chain reaches 1:3, the bulk cohesion of PU-P_1_H_3_ may match or even exceed interfacial interactions; ii) The SIC behavior of PU-P_1_H_3_ during lap shear test triggered dynamic bulk reinforcement that exceed interfacial thresholds, enabling strategic stress redistribution that preferentially initiated forced interfacial failure. In contrast, PU-P1_1_H_3_ demonstrates improved interfacial adhesion with reduced bulk strength, resulting in mixed failure mode that validates this mechanism. As for PU-P2_1_H_3_, it showed characteristic interfacial failure behavior due to weaker interfacial interactions. These differences highlight the distinct roles of hierarchical structure: PCL crystals primarily enhance bulk cohesion, while MMSIs hard domains are critical for improving interfacial interactions. The above analysis confirms that PU-P_x_H_y_ adhesives form strong interactions with functional groups (e.g., hydroxyl and metal ions) on the substrate through surface-enriched non-covalent units such as ester, urea, amide, and phenyl groups. Then, the resulting multiscale high energy phases (MMSIs hard domains and PCL crystals) become densely anchored at the adhesive-substrate interface, leading to significantly enhanced interfacial adhesion of the adhesive coatings.

To further elucidate how the multiscale structure synergistically enhances both bulk cohesion and interfacial adhesion, we performed single-cycle and cyclic 90° peeling tests on stainless steel substrates to decouple and quantify both interfacial fracture toughness and fatigue threshold^60^. As shown in Fig. 5g, PU-P_1_H_3_, PU-P1_1_H_3_, and PU-P2_1_H_3_ exhibited interfacial fracture toughness values of 4125, 913 and 2957 J m^-2^, respectively, further evidencing the dominant role of interfacial MMSIs hard domains in adhesion enhancement. Besides, PU-P_1_H_3_ yielded higher interfacial fatigue threshold (*Γ*_0_ = 1121 J m^-2^) than both counterparts (Fig. 5h), demonstrating that co-anchored MMSIs hard domains and PCL crystals effectively hold the interfacial crack tip while dissipating energy. These quantitative metrics provide compelling support for the synergistic improvement of both bulk cohesion and interfacial adhesion through multiscale engineering.

### Recyclability of PU-P_x_H_y_ adhesives

Traditional strong and tough adhesives are often irreversible, frequently hindering the partial disassembly for repair or end-of-life recycling of bonded components, which presents significant environmental challenges and economic inefficiencies. Therefore, exploring strong and tough adhesive systems with debonding-recycling capabilities is imperative for promoting greener manufacturing and facilitating the effective recovery of high-value materials.

**Fig. 6:**
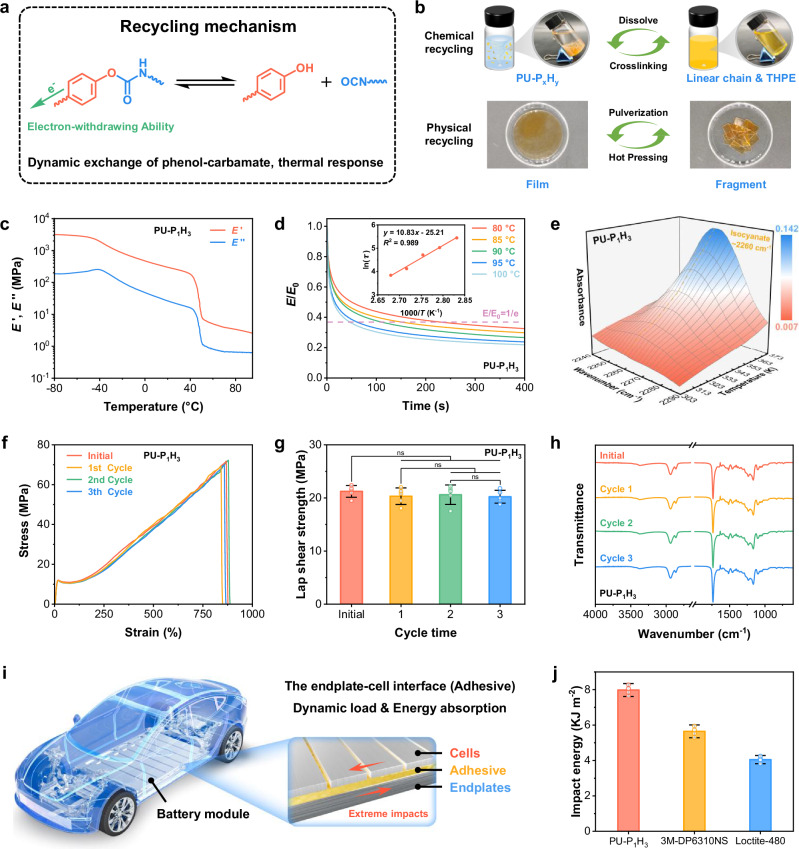
Recyclability and interfacial impact model of PU-P_x_H_y_ adhesives. **a** Recycling mechanism of PU-P_x_H_y_ adhesives. **b** Chemical and physical recycling process for PU-P_x_H_y_. **c** Temperature sweep dynamic mechanical analysis (DMA) curves of PU-P_1_H_3_ from -80 °C to 95 °C. **d** Stress relaxation curves of PU-P_1_H_3_ from 80 °C to 100 °C, with 5 °C intervals (Inset: Arrhenius fitting plot). **e** Variable-temperature FTIR spectra (2290–2240 cm^-1^) of PU-P_1_H_3_ from 30 °C to 100 °C, with 10 °C increments. Color scale represents absorbance. **f** Sress-strain curves of PU-P_1_H_3_ before and after three recycling cycles. **g** Lap shear strength of PU-P_1_H_3_ adhesive-bonded stainless steel joints before and after three recycling cycles. **h** FTIR spectra of PU-P_1_H_3_ before and after three recycling cycles. **i** Schematic showing the electric vehicle battery module and its endplate-cell interfaces. **j** Comparison of impact energy for T-type stainless steel models bonded with PU-P_1_H_3_ adhesive and two different commercial adhesives. Error bars represent mean ± SD (*n* = 5 per data point). Probability (*p*) values were determined by one-way analysis of variance, with significance levels denoted as ^ns^ *p* > 0.05, *p ≤ 0.05, **p < 0.01, and ***p < 0.001.

Unlike traditional covalent crosslinked adhesives, PU-P_x_H_y_ adhesives utilize phenol-carbamate as dynamic crosslinking points. As shown in Fig. 6a, phenol-carbamate exhibits thermally stimulated responsiveness^61–64^, promoting topological network rearrangement in PU-P_x_H_y_ and thereby conferring on-demand disassembly and recycling capability. PU-P_x_H_y_ adhesives can be recycled through both chemical and physical pathways (Fig. 6b). Specifically, PU-P_x_H_y_ adhesives bonded between components dissolve upon 15-minute immersion in 80 °C DMF, enabling chemical recycling with a mass recovery efficiency of 98.4%. This process involves thermally driven dissociation of phenol-carbamate crosslinks and dissolution of linear segments. Physical recycling is achieved by hot-pressing PU-P_x_H_y_ adhesive film fragments at 120 °C and 2.0 MPa for 20 minutes, reconstituting them into uniform adhesive films with a mass recovery efficiency above 99.5%. This process involves thermally driven phenol-carbamate crosslinking and crosslinked network rearrangement. The dynamic exchange process of phenol-carbamate was further validated. Based on fitting from temperature sweep dynamic mechanical analysis (DMA) and stress relaxation curves (Fig. 6c, d), the activation energy (*E*_a_^*^) for dynamic exchange of phenol-carbamate in PU-P_1_H_3_ was determined to be 90.01 kJ mol^-1^, with a topological freezing transition temperature (*T*_v_) of 10.5 °C. This *E*_a_^*^ falls within the typical range of activation energies for dynamic covalent bonds^7,33,65^, confirming the dynamic nature of phenol-carbamate. This dynamic reversibility is enhanced by the electron-withdrawing effect of the benzene ring, which lowers the dissociation temperature of the carbamate^61^. The low *T*_v_ indicates the potential for dynamic exchange of phenol-carbamate within the crosslinked network of PU-P_x_H_y_ below room temperature. However, the topological network rearrangement is still restricted by the weak movement of molecular chains, which is attributed to the MMSI hard phase domains and PCL crystals. Furthermore, variable-temperature FTIR spectra of PU-P_1_H_3_ reveals that the characteristic isocyanate peak (2260 cm⁻^1^) appeared and gradually intensified as the temperature increases from 30 to 100 °C, providing direct evidence for the dynamic exchange of phenol-carbamate (Fig. 6e). As shown in Fig. 6f, g, the mechanical and adhesive properties of PU-P_1_H_3_ remained largely unchanged after three recycling cycles, with tensile strength and adhesive strength retention rates up to 97.7% and 95.2%, respectively. Notably, the chemical structure of PU-P_1_H_3_ also remained consistent before and after three recycling cycles (Fig. 6h). These results demonstrate that PU-P_x_H_y_ adhesives, leveraging the dynamic exchange characteristics of crosslinking points phenol-carbamate, exhibit efficient chemical/physical recyclability, meeting the requirement for on-demand disassembly and recycling in practical applications.

### Interface impact simulation for battery module application

The PU-P_x_H_y_ adhesive-bonded stainless steel T-joint configuration was employed as an experimental model to simulate extreme impact scenarios in battery modules, demonstrating its independent load-bearing capacity and broad application potential. In new energy battery, metal endplates and frames provide macroscopic constraints. The adhesive, meanwhile, serves dual roles: compensating for microscopic assembly gaps to prevent fretting wear, and more critically, acting as a continuous medium for uniform force distribution. Consequently, its adhesive performance, especially work of debonding during extreme dynamic loads such as collisions, directly governs the safety threshold of battery packs under mechanical abuse conditions (Fig. 6i).

As shown in Supplementary Fig. 29, T-joint specimens composed of stainless steel plates and rods bonded with PU-P_1_H_3_ and two different commercial adhesives (3M-DP6310NS, Loctite-480) were subjected to pendulum impact tests to evaluate their impact energy, simulating extreme dynamic loading scenarios in battery module endplate-cell interfaces. The results showed that PU-P_1_H_3_, 3M-DP6310NS, and Loctite-480 adhesives exhibited impact energies of 7.98, 5.64, and 4.05 kJ·m^-2^, respectively (Fig. 6j). These results highlight the exceptional adhesive strength and debonding work of PU-P_1_H_3_, demonstrating its potential for applications demanding high energy absorption, impact resistance, or tolerance to sudden overloads.

## Discussion

In summary, we have successfully developed strong, ultra-tough, sustainable, and environmentally reliable polyurethane adhesives PU-P_x_H_y_ by leveraging a multiscale engineering approach that combines the advantages of thermoplastic and thermoset systems. Specifically, the multiscale structure integrates multi-level organizations, including macroscale dual-length crosslinked networks, mesoscale PCL and fibrillar crystals, nanoscale MMSIs hard phase domains, and molecular scale weak hydrogen bonds. These multi-level organizations function hierarchically and synergistically, overcoming the inherent limitations of traditional linear and crosslinked networks, thereby simultaneously enhancing the bulk cohesion and interfacial adhesion of PU-P_x_H_y_ adhesives. Consequently, the developed PU-P_1_H_3_ adhesive exhibits both high adhesive strength (21.24 MPa) and ultrahigh work of debonding (35,731 N m^-1^), representing the best combination reported to date. Meanwhile, the barrier effect of the multiscale structure endows PU-P_x_H_y_ adhesives with robust environmental reliability, including resistance to moisture, water, organic solvent, acids and alkalis, high and ultralow temperatures, and aging, which is a significant advantage over most traditional adhesives. Moreover, the dynamic crosslinked network endows PU-P_x_H_y_ adhesives with efficient chemical and physical recyclability, facilitating the effective recovery of high-value materials. This research underscores the merits of multiscale engineering for synergistic high strength-toughness and functionalization, thus pioneering an efficient and technically feasible strategy for developing strong and tough adhesives and even other advanced materials.

## Methods

### Materials

Polycaprolactone diol (HO-PCL-OH, *M*_n_ ~ 3000 g mol^-1^, 99%) was bought from Hongming Chemical Reagent Co., Ltd (Jining, China). Dicyclohexylmethane 4,4’-diisocyanate (HMDI, > 90%), hexamethylene diisocyanate (HDI, ≥ 99%), and 4,4’-diaminobenzanilide (DABA, 98%) were purchased from Aladdin Reagent Co., Ltd. (Shanghai, China). Isophthalic dihydrazide (IPDH, 98%) was obtained from Macklin (Shanghai, China). 1,1,1-tris(4-hydroxyphenyl)ethane (THPE, > 98%) was supplied by Titan Scientific Co.,Ltd. (Shanghai, China). Dibutyltin dilaurate (DBTDL, 95%) was bought from Sigma-Aldrich (USA). All reagents were used without further purification.

### Synthesis of PU-P_x_H_y_

The synthetic procedure for PU-P_x_H_y_ was carried out as described in Supplementary Fig. 1. A three-neck round bottom flask was charged with PCL (9.00 g, 3 mmol), and subjected to vacuum drying at 90 °C for 2 h to eliminate moisture. Subsequently, a mixture of HMDI (1.75 g, 6 mmol) and DBTDL (0.10 g) was introduced. The reaction was conducted under nitrogen atmosphere at 90 °C for 3 h, followed by cooling to 40 °C. At this stage, the chain extender system comprising IPDH (0.198 g, 1 mmol) and DABA (0.231 g, 1 mmol), dissolved in N,N-Dimethylformamide (DMF), was added. The reaction was maintained at 40 °C under nitrogen atmosphere for 21 h, yielding transparent and viscous prepolymer solutions, which is used for long chain crosslinking. For crosslinking, the calculated amount of THPE (Supplementary Table 1), dissolved in DMF, was introduced into the reaction system as the crosslinking agent. The mixture was heated at 40 °C under nitrogen atmosphere and stirring for 2 h. Then, HDI was added as a crosslinked short chain (Supplementary Table 1). The reaction system was further stirred at 40 °C under nitrogen atmosphere for 0.5 h. The as-obtained polymer solutions were finally poured into glass petri dishes and then subjected to thermal curing and dring at 80 °C for 48 h to obtain PU-P_x_H_y_ films.

### Preparation of PU-P_x_H_y_ adhesives

The PU-P_x_H_y_ adhesives were applied using a hot pressing method. Specifically, the adhesive films were cut into 12.5 × 20.0 mm to meet the adhesion area requirements of lap shear tests. Then, the adhesive films were placed between the overlaps of two substrates, and the adhesive layer thickness was set to 0.2 mm by using metal wires as spacers. The samples were held by clips and heated at 140 °C for 2 h, followed by cooling down to room temperature. After cooling to room temperature, the PU-P_x_H_y_-adhered samples were obtained for further testing.

### Characterization

The FTIR spectra, attenuated total reflection (ATR) spectra, and variable-temperature infrared spectra of the raw materials and PU-P_x_H_y_ were measured by VERTEX 70 spectrometer (Bruker, Germany), with the temperature range for variable-temperature measurements set from 30 °C to 150 °C. ^1^H NMR spectra of the raw materials and prepolymer were measured by the AVANCE III HD 600 instrument (Bruker, Germany). The sample was dissolved in dimethyl sulfoxide-d6 (DMSO-d6). The Raman spectra were recorded using a LabRAM HR Evolution spectrometer (HORIBA, Japan). The molecular weight of the prepolymer was determined by GPC using an Agilent-1260 Infinity II system (USA). DMF was employed as the eluent, and monodisperse polystyrene standards were used for calibration. XPS spectra of PU-P_1_H_3_ were measured by ESCALAB XI+ equipment (Thermo Fisher Scientific, USA). Differential scanning calorimetry (DSC) was performed on a Q20 calorimeter (TA Instruments, USA) with a scanning temperature range from −50 °C to 120 °C and a heating rate of 10 °C min^-1^. Thermogravimetric analysis (TGA) was measured by a TG209 F1 analyzer (NETZSCH, Germany) with a temperature range from 30 °C to 600 °C and a heating rate of 10 °C min^-1^, and the nitrogen atmosphere was used. The rheological behavior was performed using a Haake Mars40 rheometer (Thermo Fisher Scientific, USA) with a parallel-plate geometry, a temperature range of 60 °C–168 °C, a heating rate of 5 °C min^-1^, a fixed frequency of 1.0 Hz, and a strain amplitude of 1%. AFM phase images, surface elastic modulus, and adhesion force distribution maps were measured by the Dimension Icon instrument (Bruker, Germany). POM images of PU-P_1_H_3_ were observed using the Axiolab 5 microscope (ZEISS, Germany). We have an informed consent from the research participant depicted in Fig. 3c.

### Small-angle X-ray scattering (SAXS)

The in situ tensile 2D SAXS patterns of PU-P_1_H_3_ were collected on the Xeuss 2.0 instrument (Xenocs, France) equipped with a Pilatus 3 R 300 K detector and a Cu Kα X-ray source (*λ* = 1.54189 Å). The sample-to-detector distance (SDD) was set to 1188 mm, and scattering vector (*q*) range of 0.1-1.2 nm^-1^ were adopted. The average spacing of the microphase structure was calculated using Bragg’s Eq. (1):

1 $$d=\frac{2\pi }{{q}_{\max }}$$

Where *d* is the average spacing of the microstructure, and *q*_max_ is the scattering vector corresponding to the peak in the 1D SAXS profiles.

### Wide-angle X-ray scattering (WAXS)

The in situ tensile 2D WAXS patterns of PU-P_1_H_3_ were collected on the same instrument used for the SAXS tests. For WAXS tests, the SDD was 86.5 mm, and *q* range were 3.0-28.5 nm^-1^.

The weight crystallinity (*W*_c_) and volume crystallinity (*V*_c_) of PCL in PU-P_x_H_y_ were calculated based on the peak splitting fitting of 1D WAXS profiles. The *W*_c_ of PCL can be calculated by Eq. (2):

2 $${W}_{{{\rm{c}}}}=\frac{{I}_{{{\rm{c}}}}}{{I}_{{{\rm{c}}}}+{I}_{{{\rm{a}}}}}\times {{\rm{PCL\; wt}}}\%=\frac{{S}_{{{\rm{total}}}}-{S}_{{{\rm{amorphous}}}}}{{S}_{{{\rm{total}}}}}\times {{\rm{PCL\; wt}}}\%$$

where *S*_total_ represents the total area of the 1D WAXS profiles, and *S*_amorphous_ denotes the area of the amorphous region obtained by peak splitting.

Further, the *V*_c_ can be calculated using Eq. (3):

3 $${V}_{{{\rm{c}}}}=\frac{\rho -{\rho }_{{{\rm{a}}}}}{{\rho }_{{{\rm{c}}}}-{\rho }_{{{\rm{a}}}}}=\frac{\rho {W}_{{{\rm{c}}}}}{{\rho }_{{{\rm{c}}}}}$$

where *ρ*_c_ and *ρ*_a_ represent the density of PCL crystalline and amorphous region, respectively. *ρ* is the average density of PCL region and can be calculated by Eq. (4):

4 $$\frac{1}{\rho }=\frac{1-{W}_{{{\rm{c}}}}}{{\rho }_{{{\rm{c}}}}}+\frac{{W}_{{{\rm{c}}}}}{{\rho }_{{{\rm{a}}}}}$$

The calculated *W*_c_ and *V*_c_ are listed in Supplementary Table 5.

### Broadband dielectric spectroscopy (BDS)

BDS measurements of PU-P_1_H_3_ were conducted using a Novocontrol Concept 80 dielectric spectrometer equipped with a temperature control system. The samples were disk-shaped thin films with a diameter of 20 mm and a thickness of 0.65 mm. Frequency sweep mode was selected within the frequency range of 10^-1^-10^7 ^Hz at each temperature of −90 °C, −70 °C, −50 °C, and 25 °C, with temperature stability better than 0.1 °C. To comprehensively analyze the complex permittivity (ε*) data and further elucidate the underlying local molecular dynamics (*T* < *T*_g_), particularly the secondary relaxations (β and γ) in the PU-P_x_H_y_, the Havriliak-Negami (H-N) function was employed. Given the presence of multiple relaxation processes and potential contributions from DC conductivity, the total ε* can be described by Eq. (5)^66^:

5 $${\varepsilon }{*}\left(\omega \right)={\varepsilon }_{{{\infty }}}+\mathop{\sum }\limits_{k=1}^{2}\frac{{\Delta \varepsilon }_{k}}{{\left[1+{\left(i\omega {\tau }_{{{\rm{H}}}{{\rm{N}}},\,k}\right)}^{{\alpha }_{k}}\right]}^{{\beta }_{k}}}-i\frac{{\sigma }_{{{\rm{D}}}{{\rm{C}}}}}{\omega {\varepsilon }_{0}}$$

Where *ε*_∞_ is the high-frequency limit of the real part of complex permittivity (*ε*′), *ε*_0_ is the vacuum permittivity, and *σ*_DC_ represents the DC conductivity contribution at low frequencies. For each HN function (subscript *k* refers to the specific relaxation, e.g., β or γ), Δ*ε*_*k*_ is the dielectric strength, *τ*_HN, *k*_ is the characteristic relaxation time, and *α*_*k*_ and *β*_*k*_ are dimensionless parameters (typically 0 <*α*_*k*_, *β*_*k*_ ≤ 1) describing the symmetric and asymmetric broadening of the dielectric loss peak, respectively.

From the fitted HN parameters, the average relaxation time (*τ*_max, *k*_) for each relaxation process, which corresponds to the frequency (*f*_max, *k*_) where the dielectric loss (*ε*′′) passes through its maximum value, can be calculated using Eqs. (6) and (7)^67^:

6 $${\tau }_{{{\rm{m}}}{{\rm{ax}}},k}={\tau }_{{{\rm{HN}}},k}{\left[\sin \frac{\pi {\alpha }_{k}{\beta }_{k}}{2\left(1+{\beta }_{k}\right)}\right]}^{\frac{1}{{\alpha }_{k}}}{\left[\sin \frac{\pi {\alpha }_{k}}{2\left(1+{\beta }_{k}\right)}\right]}^{-\frac{1}{{\alpha }_{k}}}$$

7 $${f}_{\max,k}=\frac{1}{2\pi {\tau }_{\max,k}}$$

The temperature dependence of *τ*_max_ typically follows an Arrhenius-like behavior. This relationship can be described by the Arrhenius Eq. (8):

8 $${\tau }_{\max }={\tau }_{0}\exp \left(\frac{{E}_{{{\rm{a}}}}}{{RT}}\right)$$

where *E*_a_ is the activation energy, *T* is the absolute temperature, *τ*_0_ is the pre-exponential factor, and *R* is the gas constant.

### Dynamic mechanical analysis (DMA)

The stress relaxation and temperature sweep tests were conducted on a Q850 rheometer (TA Instruments, USA). Stress relaxation tests were performed at 80 °C, 85 °C, 90 °C, 95 °C, and 100 °C, with a 5-minute temperature equilibration and 1% strain. Temperature sweep tests were carried out over a temperature range of -80 °C–100 °C at a heating rate of 5 °C min^-1^ and a frequency of 1 Hz. The activation energy (*E*_a_) of PU-P_1_H_3_ was obtained by fitting, using the Arrhenius Eq. (9)^68^:

9 $$\tau \left(T\right)={\tau }_{0}\exp \left(\frac{{E}_{a}}{{RT}}\right)$$

Where *τ* is the relaxation time, *R* is the gas constant, and *T* is the temperature. *T*_v_ was calculated using Eqs. (10) and (11)^69^:

10 $$\eta=G{{\cdot }}{\tau }{*}$$

11 $$G=\frac{{E}^{{\prime} }}{2\left(1+v\right)}\approx \frac{{E}^{{\prime} }}{3}$$

Where *η* is the viscosity, *G* is the shear modulus, *υ* is the Poisson’s ratio (typically, 0.5 for rubber material), *E*’ is the tensile modulus of the rubber plateau region measured by temperature sweep tests.

### Mechanical test

The mechanical tensile properties of PU-P_x_H_y_ were measured using a UTM2203 instrument (SUNS, China) with a testing speed of 50 mm min^-1^. The impact resistance was evaluated using a PTM700 pendulum impact tester (SUNS, China).

### Adhesion measurement

Lap shear tests were conducted on PU-P_x_H_y_ adhesive-bonded joints using a Z010 instrument (Zwick Roell, Germany) at a testing speed of 3 mm min^-1^. The substrate overlap area was 12.5 × 20.0 mm. Lap shear strength, referred to as adhesive strength in this article, is defined as the maximum debonding force of the joint divided by the overlap area, as shown in Eq. (12)^3^:

12 $${{\rm{L}}}{{\rm{ap}}}\,{{\rm{s}}}{{\rm{h}}}{{\rm{ear}}}\,{{\rm{strengt}}}{{\rm{h}}}\,\left({{\rm{MPa}}}\right)=\frac{{{\rm{F}}}{{\rm{orce}}}\,\left({{\rm{N}}}\right)}{{{\rm{Adhesive\; area}}}\left({{{\rm{mm}}}}^{2}\right)}$$

Work of debonding is defined as the integral area under the force-extension curve divided by the overlap area, as shown in Eq. (13)^3^:

13 $${{\rm{Work}}}\; {{\rm{of}}}\; {{\rm{debonding}}}\,\left({{\rm{N}}}\;{{{\rm{m}}}}^{-1}\right)=\frac{{{\rm{Integral}}}\; {{\rm{area}}}\left({{\rm{N}}}{{\cdot }}{{\rm{mm}}}\right)}{{{\rm{Adhesive}}}\; {{\rm{area}}}\left({{{\rm{mm}}}}^{2}\right)}\times {10}^{3}$$

The 90° peeling test was conducted to evaluate the interfacial fracture toughness of PU-P_1_H_3_ adhesive to stainless steel substrates (ASTM D2861). The adhesive, thickness-controlled by a mold, was sandwiched between a plasma-treated PET backing and a stainless steel substrate for hot-pressing bonding. During testing, the sample (110 × 20 × 0.5 mm) was mounted on a 90° peel fixture, with the stainless steel substrate fixed horizontally and the PET backing clamped to the force sensor of UTM2203 instrument (SUNS, China). The backing was peeled at a constant 90° angle (50 mm min^-1^) via a pulley system. Interfacial fracture toughness was calculated as the average steady-state peel force (*F*_s_) divided by the specimen width.

The cyclic 90° peeling test was conducted to characterize the interfacial fatigue threshold of PU-P_1_H_3_ adhesive to stainless steel substrates. The methodology involved applying cyclic peeling forces *F*_a_ (*F*_a_ < *F*_s_) over *N* cycles while monitoring the interfacial crack extension (*c*) as a function of cycle number *N*. Accordingly, the applied energy release rate *G* can be calculated as the *F*_a_ divided by the specimen width, and the interfacial crack propagation rate as d*c*/d*N*. Through systematic variation of applied force amplitudes, the characteristic d*c*/d*N*-*G* relationship was established, from which *Γ*_0_ was extrapolated as the *G*-axis intercept of the linear regression.

### Binding energy calculation

Theoretical computations were executed employing the density functional theory (DFT) method in Gaussian 16 program package^70^. Geometry optimizations and frequency analyses were conducted at the B3LYP/6-311 G(d,p) level for MMSIs unit and B3LYP/Def2svp/SDD level for interfacial adhesion, incorporating the D3(BJ) empirical dispersion correction in the gas phase. After that, conformations lacking imaginary frequencies in their vibrational spectra were considered stable. Single-point energy calculations for all stable conformations were then carried out at the M062X-D3/def2-TZVP level for MMSIs unit and B3LYP/Def2TZVP/SDD level for interfacial adhesion to obtain the binding energies (Δ*E*). The basis set superposition error energy (*E*_BSSE_) was corrected using the standard counterpoise method. The Δ*E* values of dimers and tetramers were evaluated by Eqs. (14) and (15), respectively:

14 $$\Delta E={E}_{{{\rm{W}}}{{\rm{X}}}}-{E}_{{{\rm{W}}}}-{E}_{{{\rm{X}}}}+{E}_{{{\rm{B}}}{{\rm{SSE}}}}$$

15 $$\Delta E={E}_{{{\rm{W}}}{{\rm{XYZ}}}}-{E}_{{{\rm{W}}}}-{E}_{{{\rm{X}}}}-{E}_{{{\rm{Y}}}}-{E}_{{{\rm{Z}}}}+{E}_{{{\rm{B}}}{{\rm{SSE}}}}$$

For MMSIs unit: Where W, X, Y, and Z represent the four supramolecular monomers. Specifically, the dimer WX is composed of W and X, while the tetramer WXYZ consists of W, X, Y, and Z. The calculated single-point DFT energies for dimer WX and tetramer WXYZ are represented by *E*_WX_ and *E*_WXYZ_, respectively. The total energies of individual monomers are denoted as *E*_W_, *E*_X_, *E*_Y_, and *E*_Z_. The optimized conformations were visualized via VESTA software.

For interfacial adhesion: The IRI analysis was then performed using the Multiwfn program (3.8 dev). The resulting IRI data were visualized using VMD software, allowing for clear depiction of interaction regions and their spatial distributions.

### Statistical analysis

Mechanical and adhesion performance were determined from the average of five samples, and adhesive strength was reported as the means ± standard deviation. Probability (*p*) values were determined by one-way analysis of variance (ANOVA). Significance levels were indicated as ^ns^*p* > 0.05, ^*^p ≤ 0.05, ^**^p < 0.01, and ^***^p < 0.001. The data and its visualization were processed by Origin software.

## Supplementary information

Supplementary Information

Transparent Peer Review file

## Source data

Source Data

## Data Availability

The data supporting the findings of this study are available within the Article and its Supplementary Information. All data are available from the corresponding author upon request. Source data are provided with this paper.
