# Supplementary material for: A multiscale structure enabled strong, ultra-tough and sustainable polyurethane adhesives
Source: Nat Commun. 2026 May 6;17:6091. doi: 10.1038/s41467-026-72658-4 (PMC13385818; doi:10.1038/s41467-026-72658-4)
Supplement: Supplementary file 1 — Supplementary Information [file 41467_2026_72658_MOESM1_ESM.pdf]

Supplementary Information for

A multiscale structure enabled strong, ultra-tough and sustainable polyurethane adhesives

Yubing Fu[1,†], Xinyu Chen[1,†], Liwei Lu[1], Wenwei Yang[1], Siyu Gan[1], Xueling Yan[1] & Lan Liu[1,*]

[1]School of Materials Science and Engineering, Key Lab of Guangdong Province for High Property and Functional Macromolecular Materials, South China University of Technology, Guangzhou, 510641, P. R. China.

[†]These authors are equal contribution first authors.

*Corresponding author: Lan Liu (psliulan@scut.edu.cn)

This PDF file includes:

Supplementary Figures 1-29……………..………………………………………….……......2

Supplementary Tables 1-11..…………………………………………………...…..........19

Supplementary References……………………….…………………………………........25

Supplementary Figures

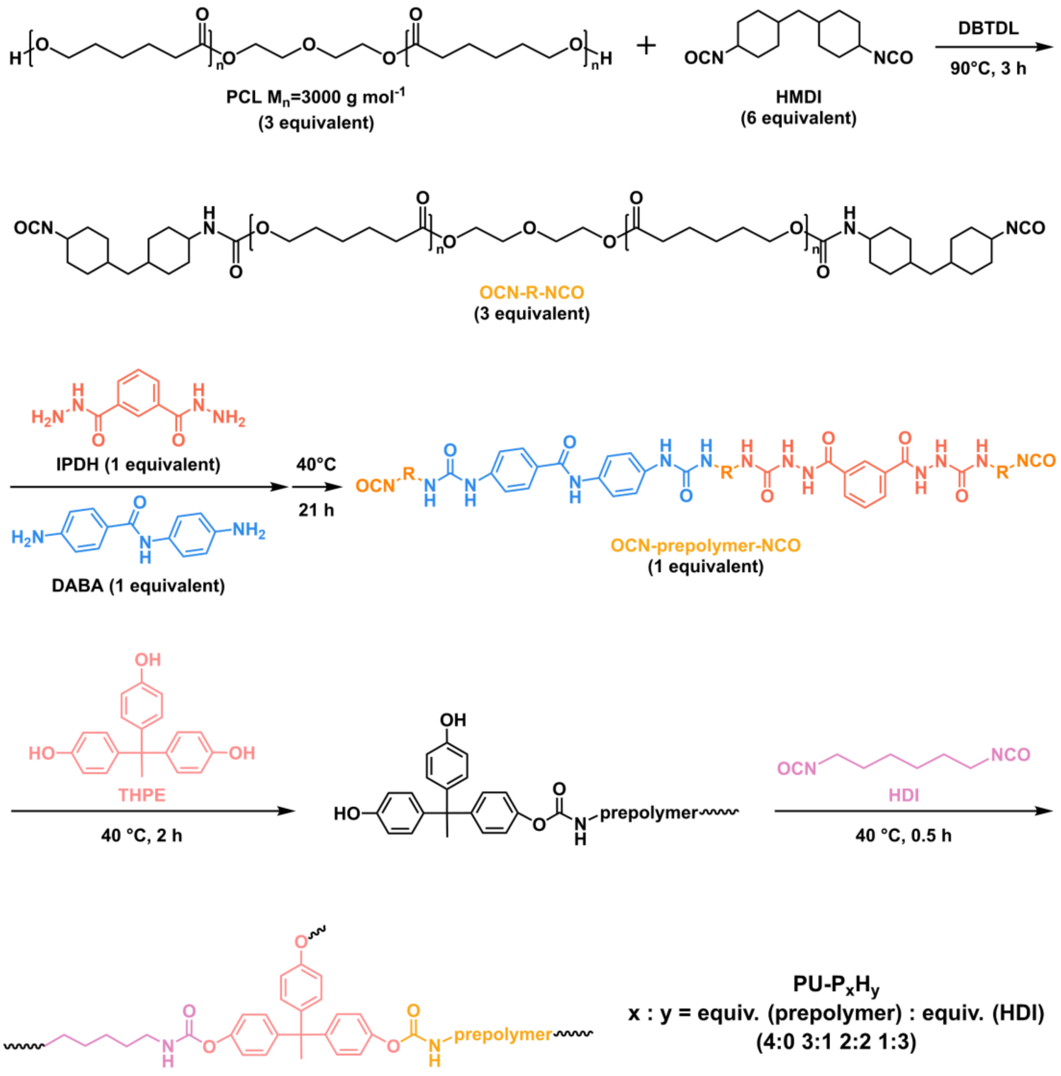

Supplementary Fig. 1 | The synthetic route of PU-P_xH_y adhesives with a dual-length dynamic crosslinked network and multiscale structure.

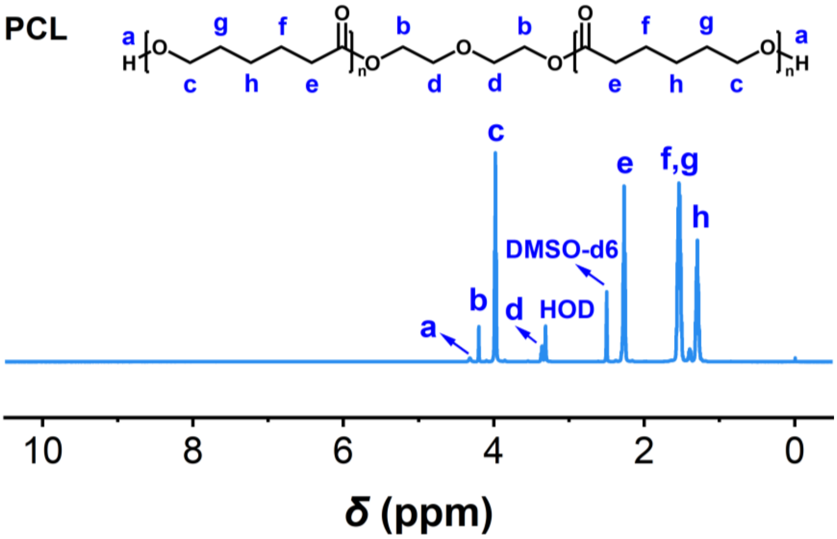

Supplementary Fig. 2 | ^{1}H NMR spectrum of PCL (600 MHz, DMSO-d6). PCL ^{1}H NMR (600 MHz, DMSO-d6): δ 4.32 (s, 1H), 4.20 (s, 2H), 3.98 (t, J = 6.6 Hz, 32H), 3.36 (t, J = 6.6 Hz, 3H), 2.27 (q, J = 7.0 Hz, 35H), 1.54 (dp, J = 15.1, 7.2 Hz, 68H), 1.30 (h, J = 7.3, 6.4 Hz, 35H).

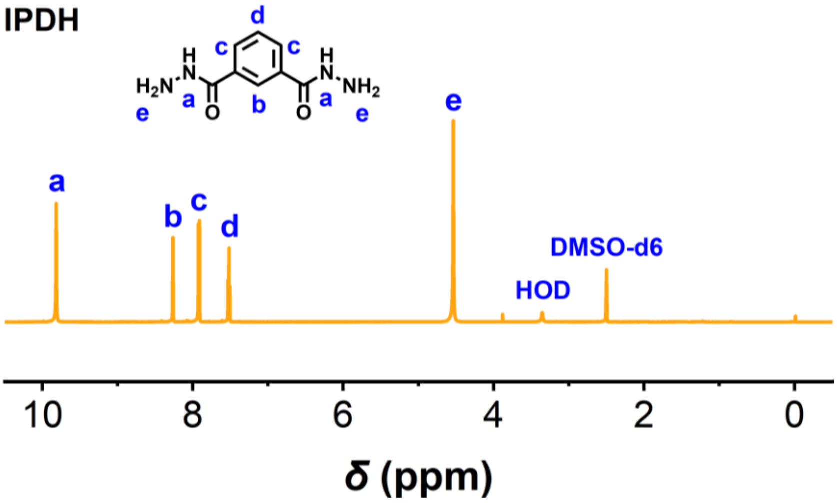

Supplementary Fig. 3 | ^{1}H NMR spectrum of IPDH (600 MHz, DMSO-d6). IPDH ^{1}H NMR (600 MHz, DMSO-d6): δ 9.82 (s, 2H), 8.26 (s, 1H), 7.92 (dd, J = 7.7, 1.8 Hz, 2H), 7.52 (s, 1H), 4.54 (s, 4H).

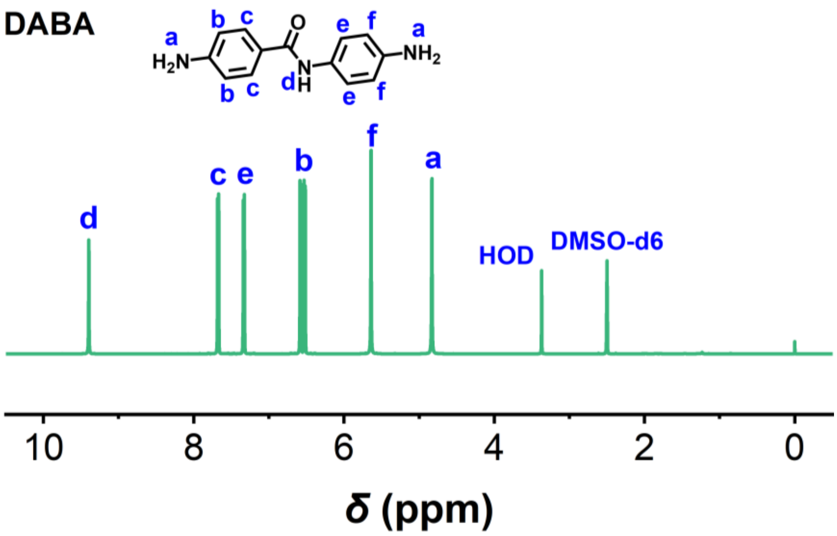

Supplementary Fig. 4 | ^{1}H NMR spectrum of DABA (600 MHz, DMSO-d6). DABA ^{1}H NMR (600 MHz, DMSO-d6) δ 9.40 (s, 1H), 7.68 (d, J = 8.6 Hz, 2H), 7.33 (d, J = 8.7 Hz, 2H), 6.55 (dd, J = 33.1, 8.7 Hz, 4H), 5.64 (s, 2H), 4.83 (s, 2H).

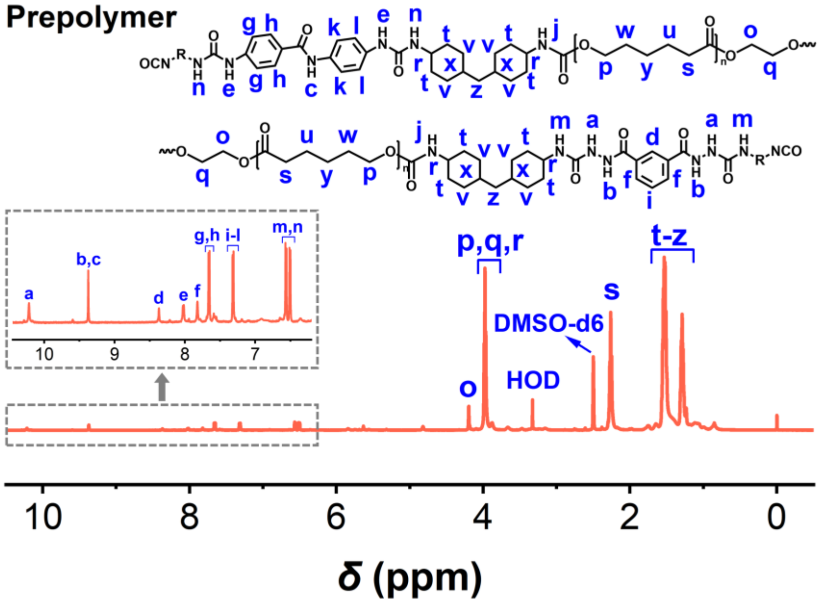

Supplementary Fig. 5 | ^{1}H NMR spectrum of prepolymer (600 MHz, DMSO-d6). Prepolymer ^{1}H NMR (600 MHz, DMSO-d6): δ 10.22 (s, 2H), 9.37 (s, 2H), 8.41 (s, 1H), 8.06 (s, 2H), 7.82 (s, 2H), 7.72–7.60 (m, 3H), 7.38–7.26 (m, 3H), 6.56 (d, J = 8.5 Hz, 8H), 4.20 (s, 11H), 3.98 (t, J = 6.6 Hz, 137H), 2.26 (t, J = 7.4 Hz, 140H), 1.53 (dq, J = 14.8, 7.5 Hz, 348H), 1.35–1.03 (m, 197H).

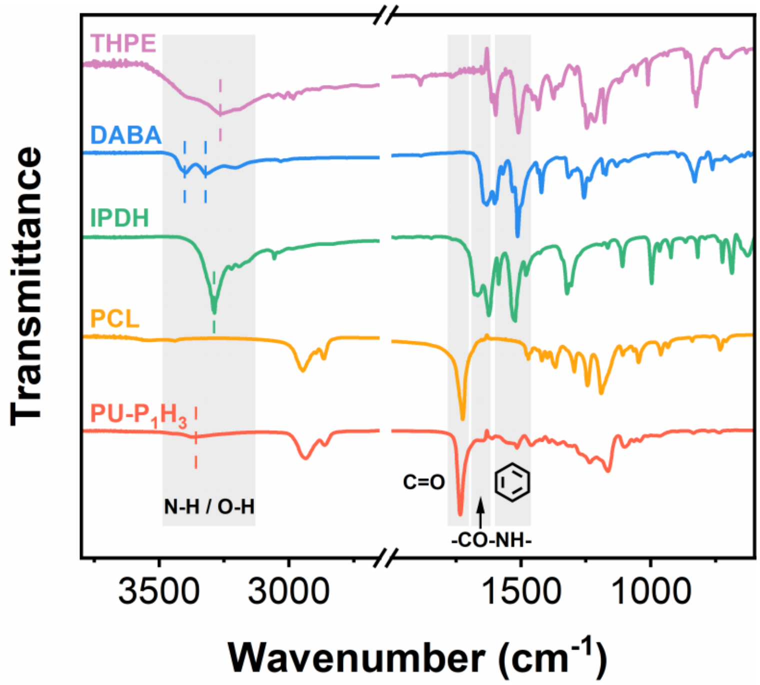

Supplementary Fig. 6 | FTIR spectra of PU-P_1H_3 adhesive and its raw materials (including IPDH, DABA, THPE, and PCL).

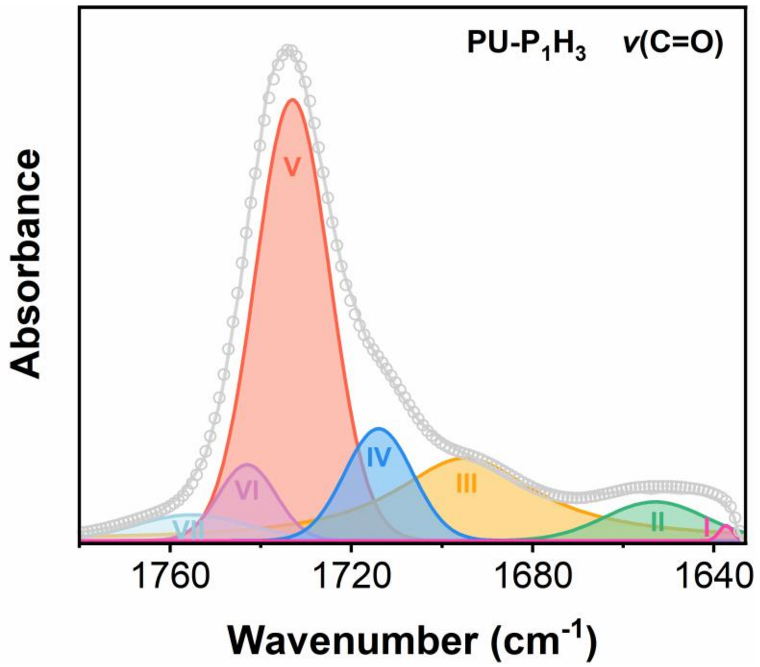

Supplementary Fig. 7 | Deconvolution analysis of the infrared C=O stretching vibration peaks (1780 ~ 1630 cm^{-1}) in PU-P_1H_3 adhesive.

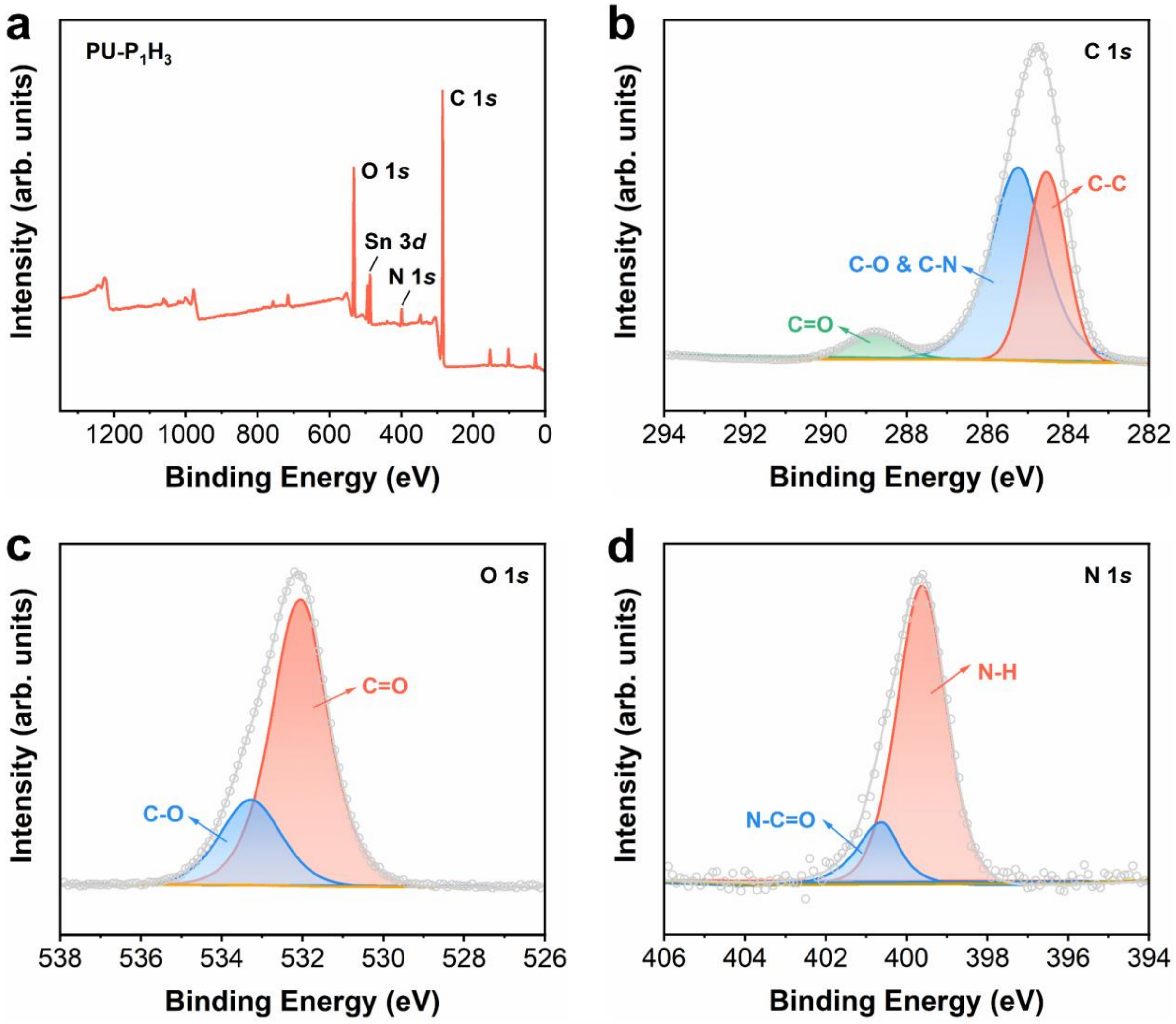

Supplementary Fig. 8 | XPS characterization of PU-P_1H_3 adhesive. a XPS survey, **b** C 1*s*, **c** O 1*s*, and **d** N 1*s* spectra.

The characteristic peaks of PU-P_1H_3 at 532, 399, and 285 eV correspond to O 1*s*, N 1*s*, and C 1*s*, respectively. The C 1*s* spectrum can be divided into three peaks, C=O (288.80 eV), C-O & C-N (285.25 eV) and C-C (284.55 eV). The O 1*s* spectrum can be divided into two peaks, C-O (533.30 eV) and C=O (532.05 eV). The N 1*s* spectrum can be divided into two peaks, N-C=O (400.65 eV) and N-H (399.60 eV).

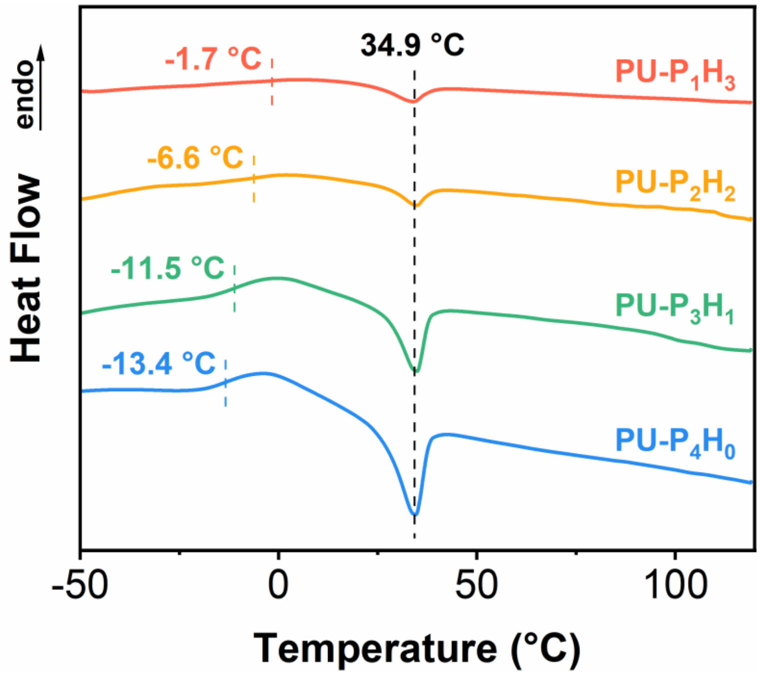

Supplementary Fig. 9 | DSC curves of PU-P_xH_y adhesives.

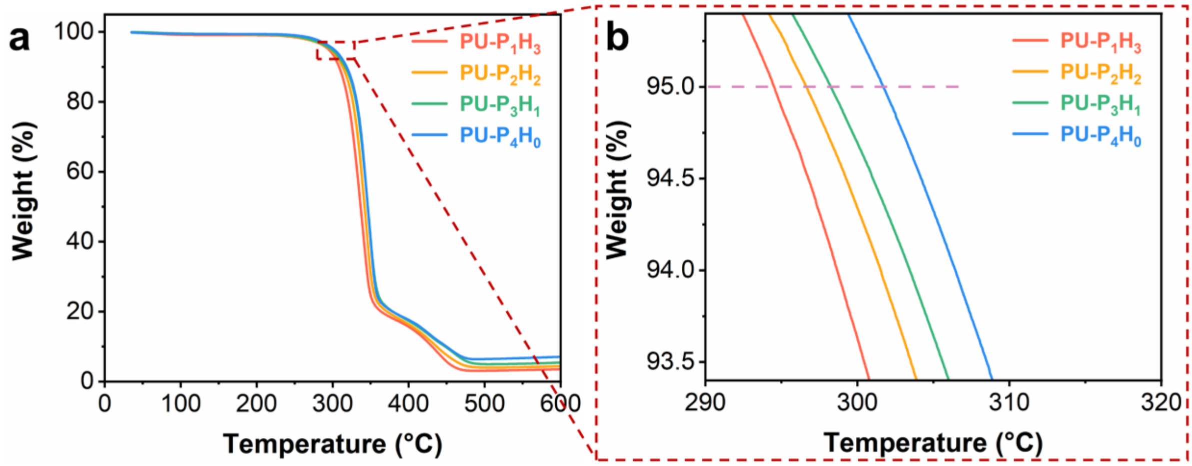

Supplementary Fig. 10 | TG analysis. a TG curve of PU-P_xH_y adhesives and **b** its local enlarged image.

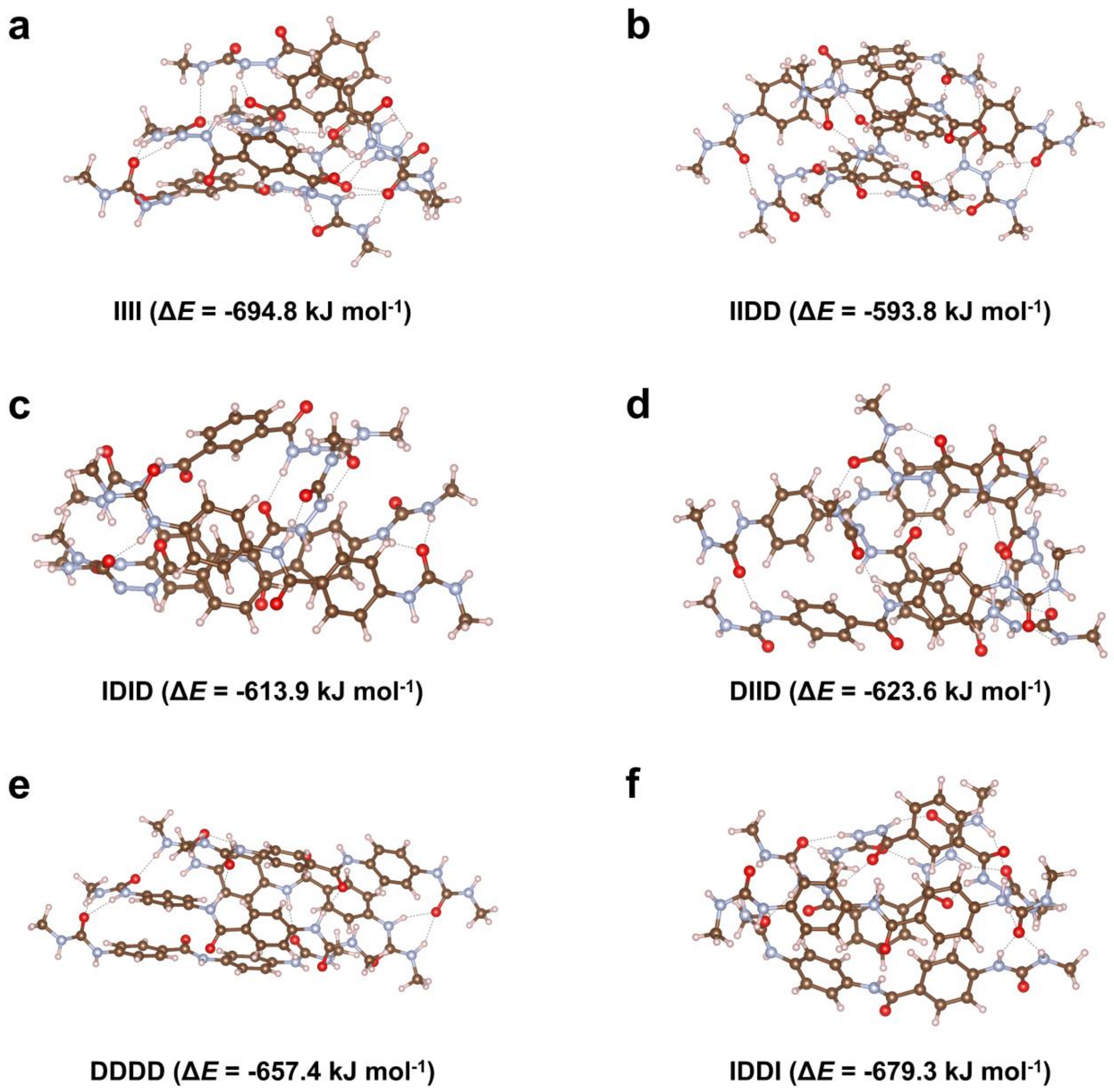

Supplementary Fig. 11 | The structure and binding energies of tetramers. a IIII, **b** IIDD, **c** IDID, **d** DIID, **e** DDDD, and **f** IDDI.

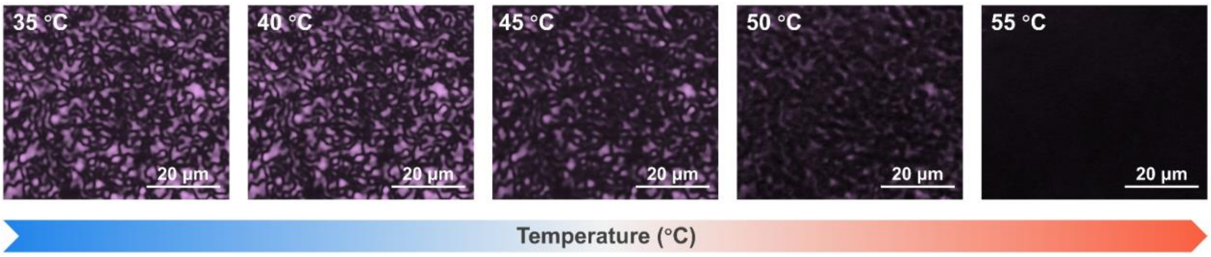

Supplementary Fig. 12 | Variable-temperature polarized optical microscopy images of PU-P_1H_3 from 35 to 55 °C, with 5 °C increments.

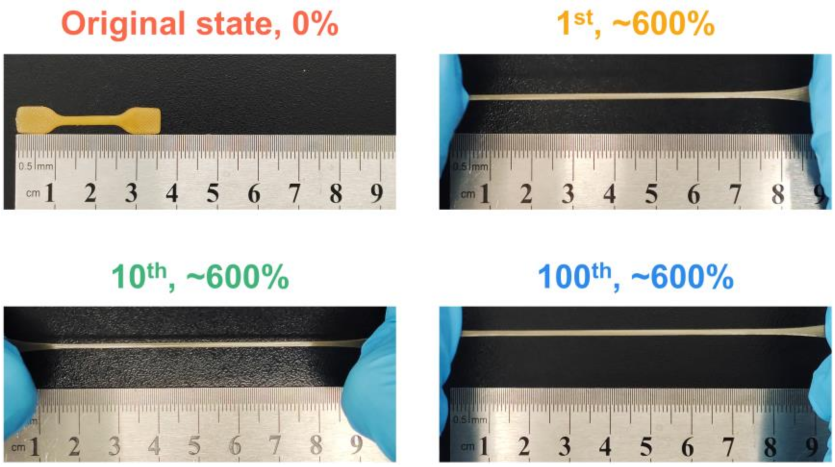

Supplementary Fig. 13 | Optical photos showing strain-induced crystallization of PU-P_1H_3 over multiple tensile cycles (600% strain).

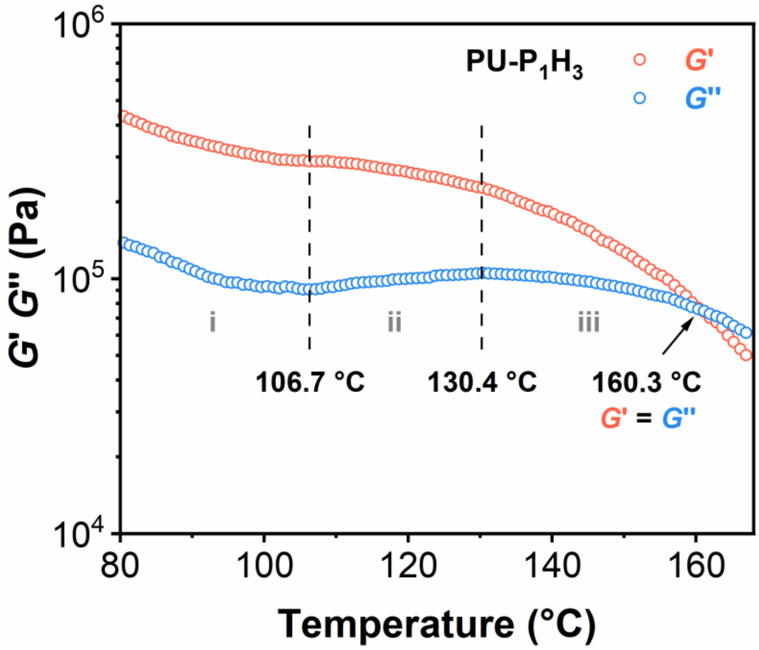

Supplementary Fig. 14 | Temperature-sweep rheological curves of PU-P_1H_3 from 80 to 168 °C.

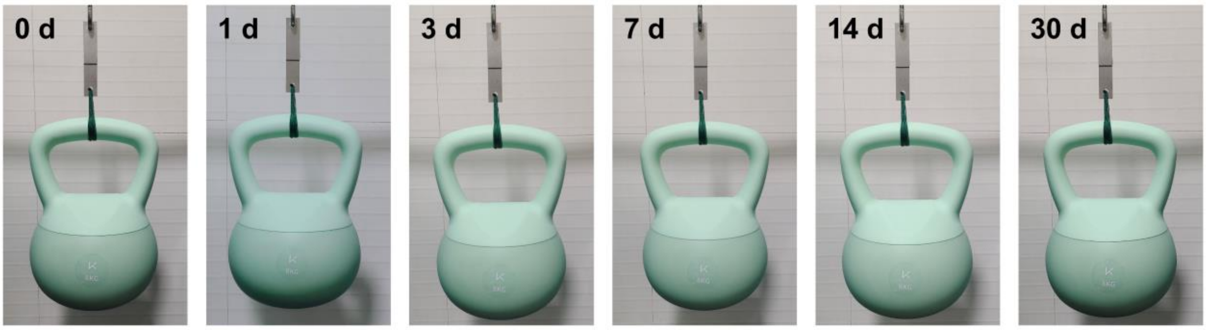

Supplementary Fig. 15 | Images of the PU-P_1H_3 adhesive-bonded stainless steel joints over a 30-day period under a sustained 8 kg load, showing no detectable creep or adhesive failure.

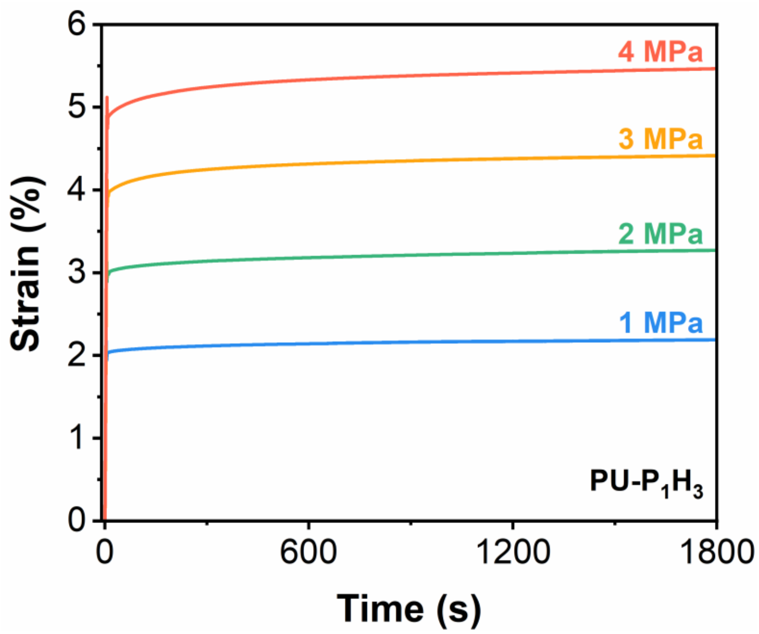

Supplementary Fig. 16 | Creep of the PU-P_1H_3 adhesive-bonded stainless steel joints at constant lap shear stress levels corresponding to 1, 2, 3, and 4 MPa.

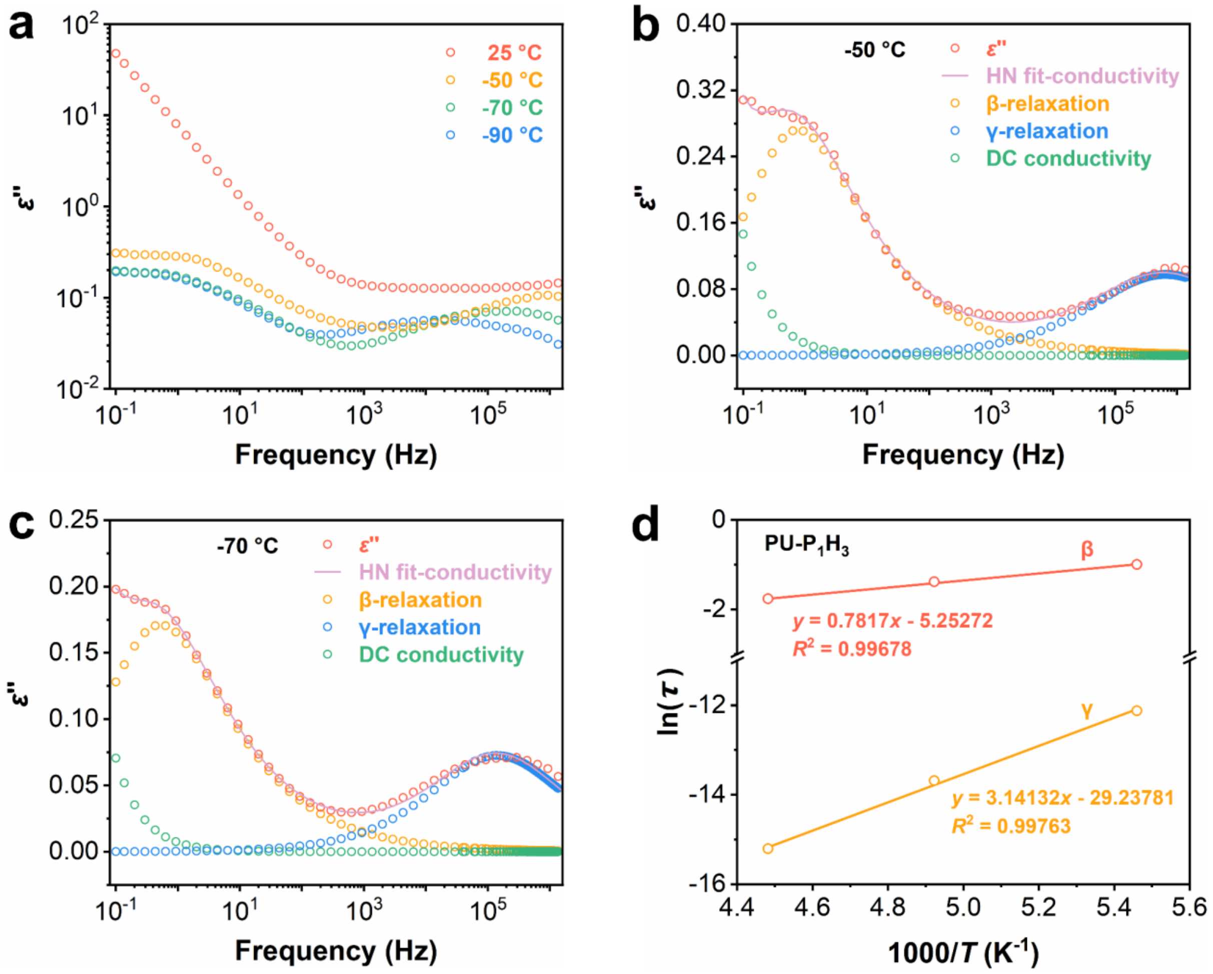

Supplementary Fig. 17 | BDS spectra of PU-P_1H_3 adhesive. a Dielectric loss (ε'') spectra of PU-P_1H_3 adhesive at 25, -50, -70, and -90 °C. Dielectric loss spectra and its Havriliak-Negami (H-N) of PU-P_1H_3 adhesive at **b** -50 °C and **c** -70 °C. **d** Arrhenius fitting of the secondary relaxations (β and γ relaxations) of PU-P_1H_3 adhesive.

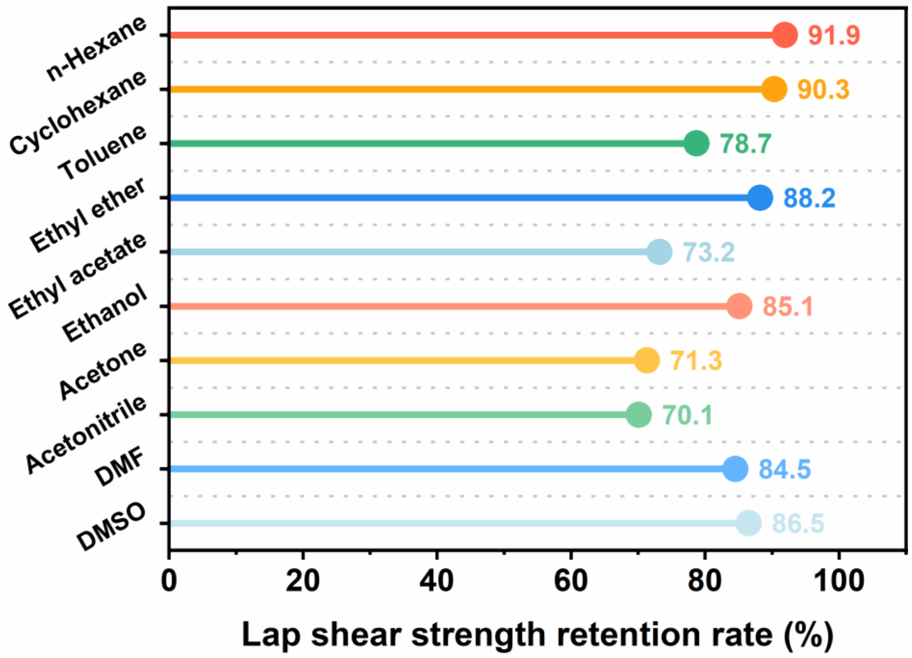

Supplementary Fig. 18 | Retention rate of lap shear strength of PU-P_1H_3 adhesive-bonded stainless steel joints after 24-hour immersion in 10 different organic solvents.

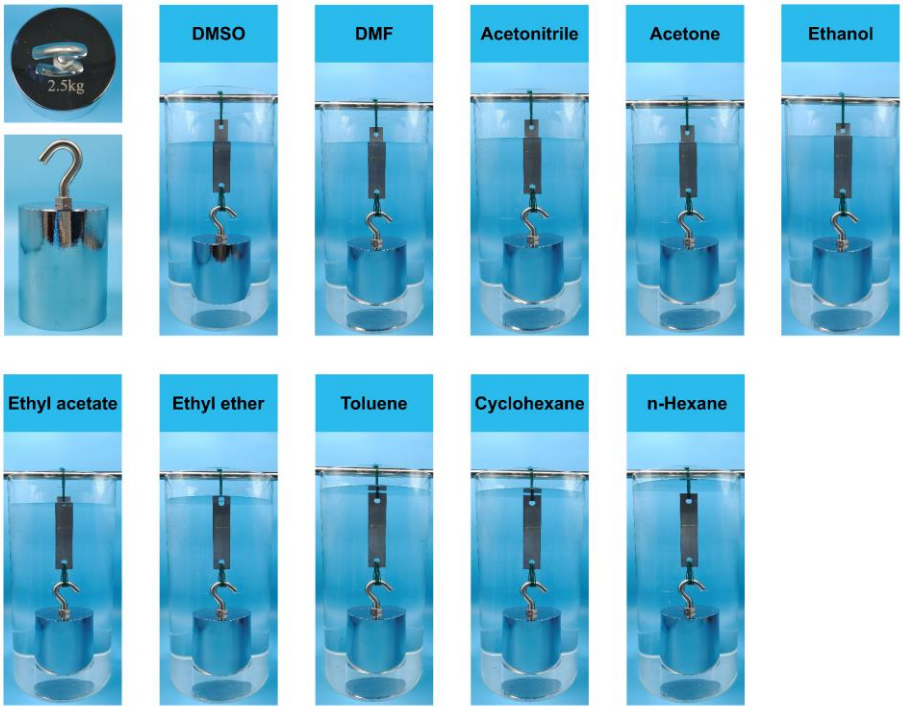

Supplementary Fig. 19 | Images showing stainless steel joints bonded with PU-P_1H_3 adhesive stably supporting a 2.5 kg weight in various organic solvents after immersion for 24 h.

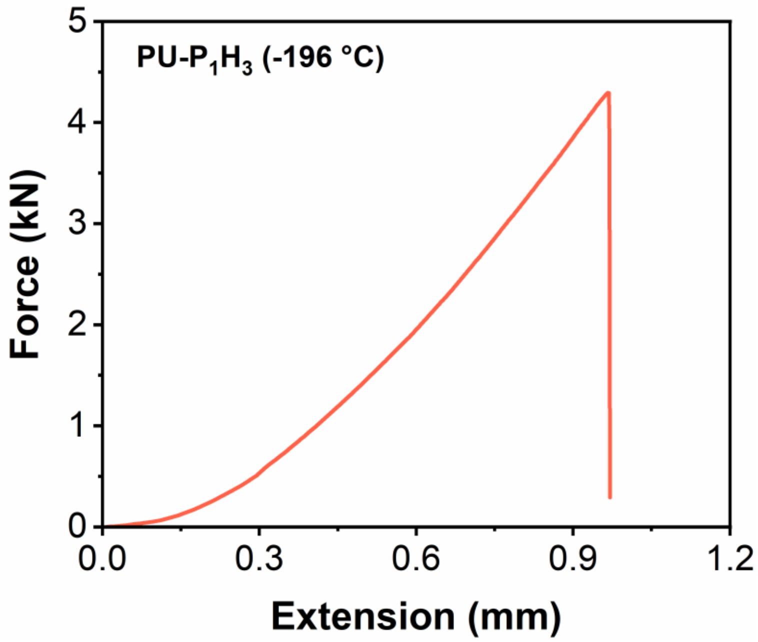

Supplementary Fig. 20 | Force-extension curve from lap shear test of PU-P_1H_3 adhesive at -196 °C.

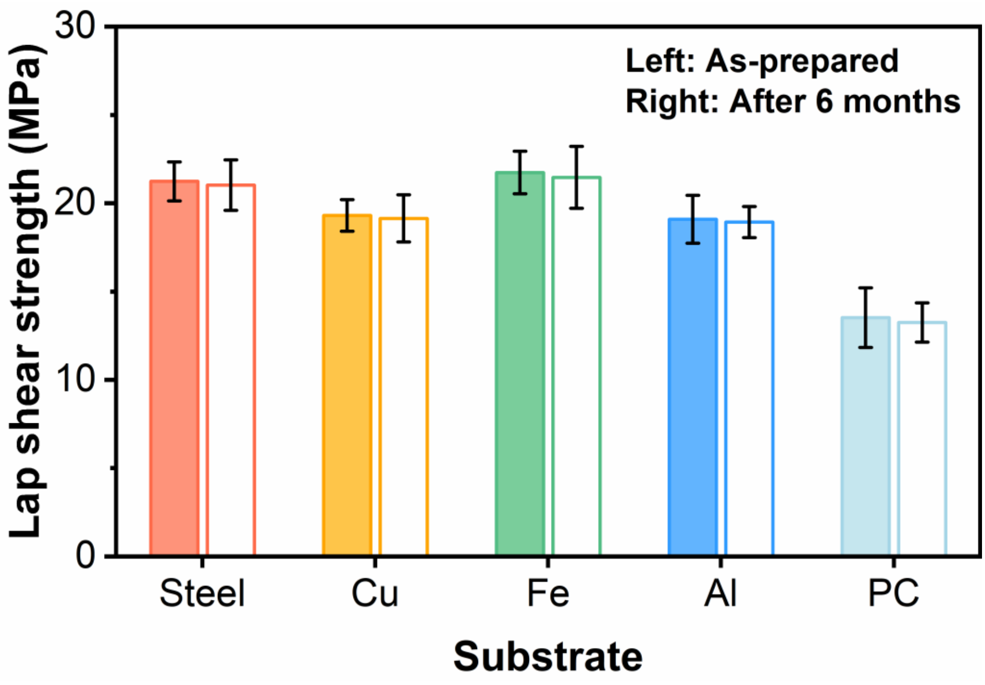

Supplementary Fig. 21 | Comparison of lap shear strength of PU-P_1H_3 adhesive bonded on different substrates before and after storage under ambient conditions for 6 months. Error bars represent mean ± SD (n=5 per data point).

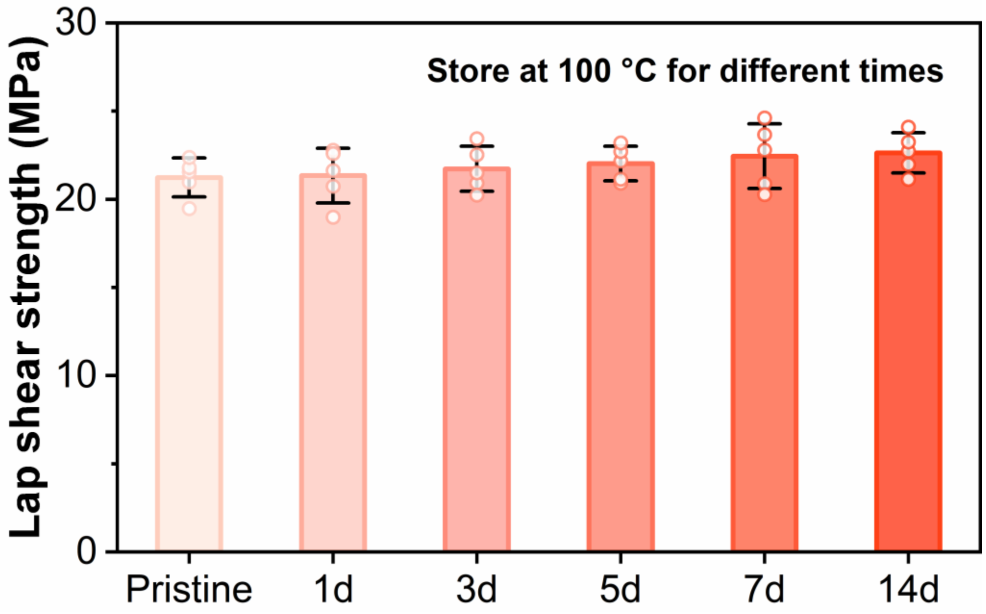

Supplementary Fig. 22 | Lap shear strength of PU-P_1H_3 ahesive-bonded stainless steel joints after thermal aging at 100 °C for 14 days. Error bars represent mean ± SD (n=5 per data point).

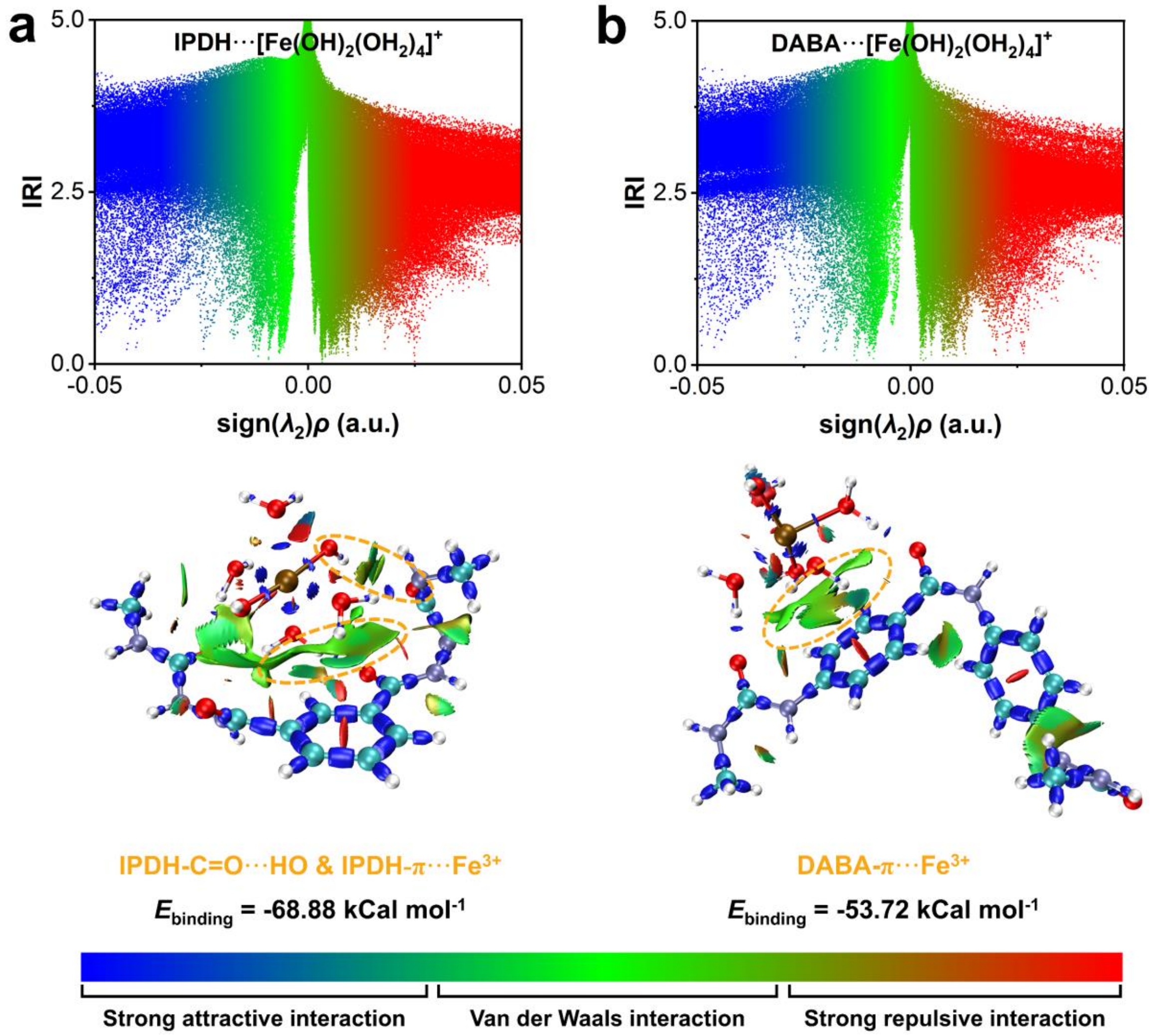

Supplementary Fig. 23 | Plots of IRI versus sign(λ_2)ρ and the gradient isosurfaces for the complexes: **a** IPDH···$[Fe(OH)_2(OH_2)_4]^+$, **b** DABA···$[Fe(OH)_2(OH_2)_4]^+$.

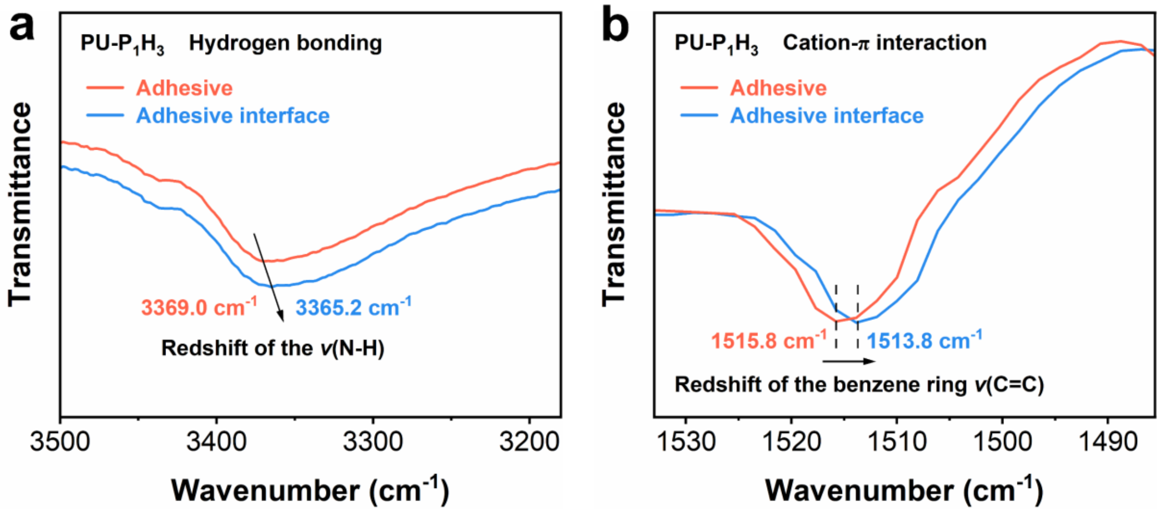

Supplementary Fig. 24 | FTIR spectra of PU-P1$_1$H$_3$ before and after interfacial failure with stainless steel substrate. a N-H stretching vibration, **b** C=C stretching vibration of benzene ring.

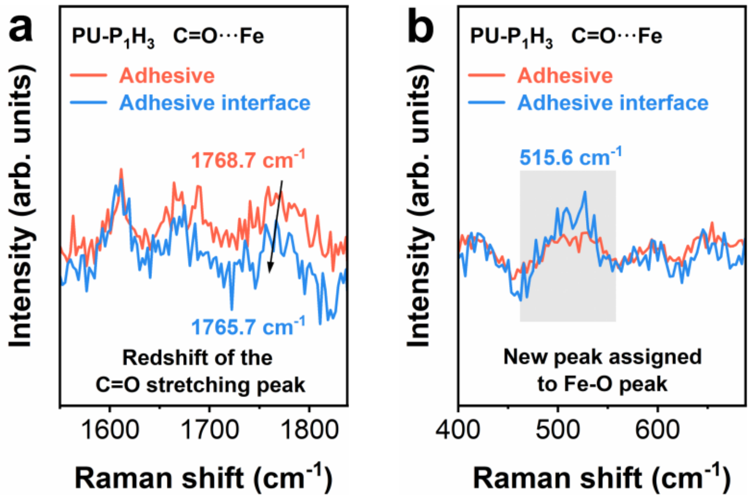

Supplementary Fig. 25 | Raman spectra of PU-P$_1$H$_3$ before and after interfacial failure with stainless steel substrate. a C=O stretching peak, **b** new Fe-O peak formed at the adhesive interface.

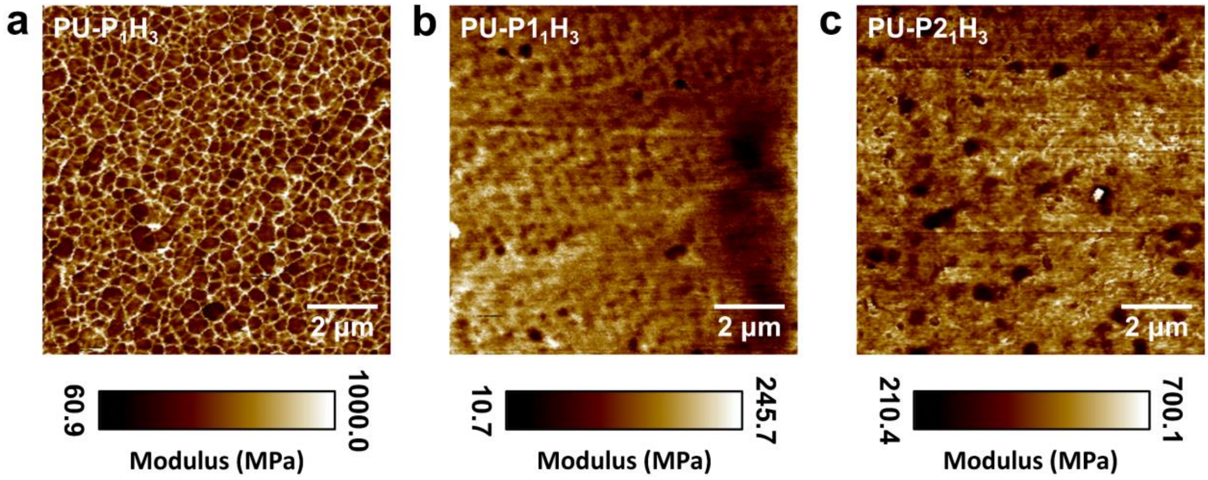

Supplementary Fig. 26 | AFM-based surface modulus measurements. a PU-P_1H_3, **b** PU-$P1_1H_3$, and **c** PU-$P2_1H_3$.

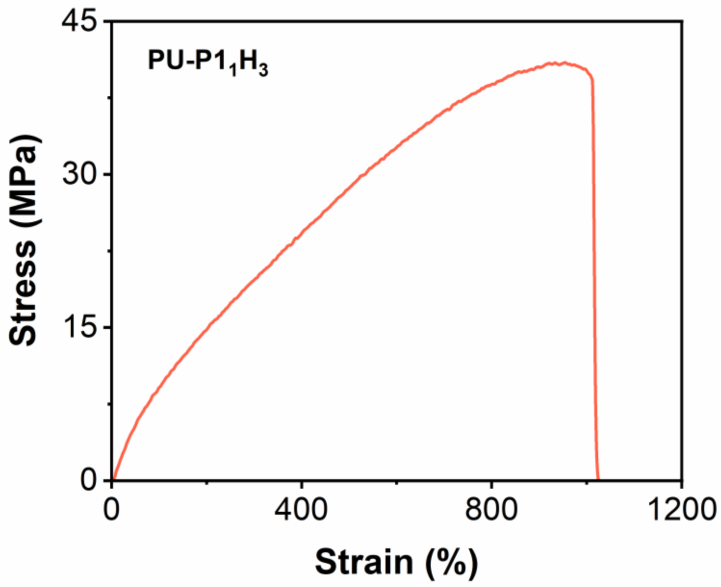

Supplementary Fig. 27 | Stress-strain curve of PU-$P1_1H_3$.

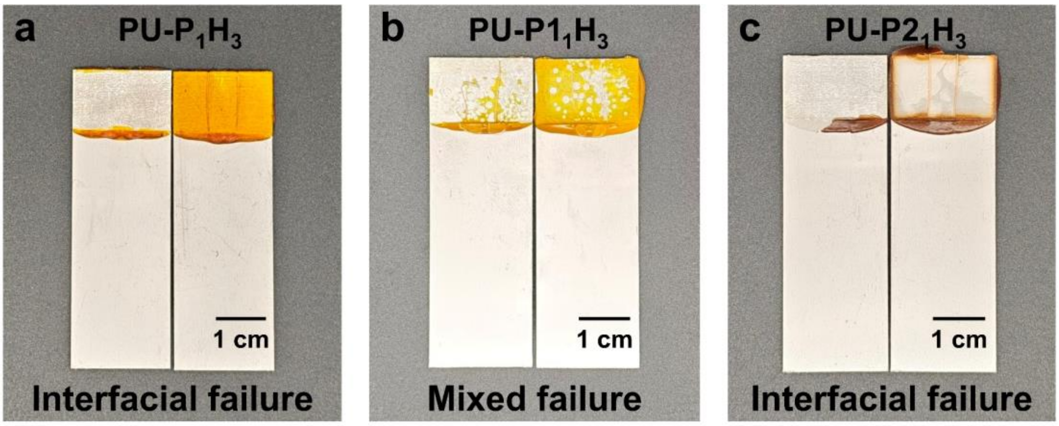

Supplementary Fig. 28 | Failure modes. a Interfacial failure (PU-P_1H_3), **b** mixed failure (PU-$P1_1H_3$), and **c** interfacial failure (PU-$P2_1H_3$).

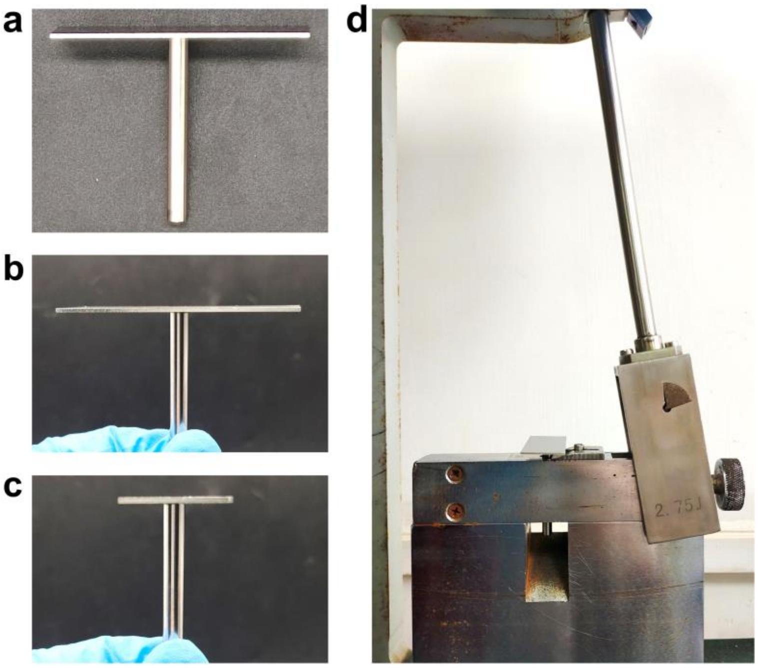

Supplementary Fig. 29 | Optical images showing PU-P_1H_3 adhesive-bonded stainless steel T-joint specimen during impact testing.

Supplementary Tables

Supplementary Table 1 | Synthesis formula of PU-P_xH_y adhesives.

Samples	PCL (mmol)	HMDI (mmol)	IPDH (mmol)	DABA (mmol)	THPE (mmol)	HDI (mmol)
PU-P_1H_3	3	6	1	1	2.67	3
PU-P_2H_2	3	6	1	1	1.33	1
PU-P_3H_1	3	6	1	1	0.89	0.33
PU-P_4H_0	3	6	1	1	0.67	0

Supplementary Table 2 | Thermal properties of PU-P_xH_y adhesives.

Samples	T_g (°C)	$T_{d,5\%}$ (°C)	Residual mass fraction (%)
PU-P_1H_3	-1.7	294.5	3.53
PU-P_2H_2	-6.6	296.6	4.41
PU-P_3H_1	-11.5	298.2	5.43
PU-P_4H_0	-13.4	301.7	7.07

Supplementary Table 3 | Mechanical properties of PU-P_xH_y.

Samples	Tensile modulus (MPa)	Tensile strength (MPa)	Ductility (%)	Toughness (MJ m^{-3})
PU-P_4H_0	35.61	44.09	1080.5	224.67
PU-P_3H_1	43.10	58.51	1028.8	294.67
PU-P_2H_2	69.33	72.99	972.2	328.19
PU-P_1H_3	110.84	71.83	868.1	315.73

Supplementary Table 4 | Comparison of mechanical properties between PU-P_1H_3 and reported thermosetting elastomers with high strength and toughness.

Samples	Tensile strength (MPa)	Ductility (%)	Toughness (MJ m^{-3})	Ref.
PU-P_1H_3	71.83	868.1	315.73	Our work
$S_{0.05}N_{0.11}C_{0.11}$	50.00	1500.0	300.00	1
ODAPU0	44.20	872.0	118.00	2
HPU-$D_{3.5}$	35.60	579.5[a)]	111.32[a)]	3
DMG-MCPU	64.53	605.2[a)]	266.39	4
PTU2	44.85[a)]	418.9[a)]	121.23[a)]	5
PASC-HM-0.04	61.20	193.0	97.00	6
PTUU-N_3	18.02	635.0	35.53	7
Cu-DOU-CPU	14.80	1182.0	87.00	8
POU 6b	49.10	677.0	125.12[a)]	9

[a)] indicates data estimated from figures in the literature.

Supplementary Table 5 | The weight and volume crystallinity of PU-P_1H_3 under different strains calculated from 1D WAXS profiles.

PU-P_1H_3	0%	200%	500%
W_c (%)	18.50	17.19	18.46
V_c (%)	17.50	16.25	17.46

Supplementary Table 6 | Signs of cross peaks in synchronous spectrum (corresponding to Fig. 2j).

1656	+	+	+	
1694	+	+		
1743	+			
1755				
	1755	1743	1694	1656

Supplementary Table 7 | Signs of cross peaks in asynchronous spectrum (corresponding to Fig. 2j).

1656	–	–	+	
1694	–	–		
1743	+			
1755				
	1755	1743	1694	1656

Supplementary Table 8 | Final results of the multiplication of the signs of each cross peak in 2DCOS synchronous and asynchronous spectra of PU-P_1H_3.

1656	–	–	+	
1694	–	–		
1743	+			
1755				
	1755	1743	1694	1656

Supplementary Table 9 | Lap shear strength and work of debonding of PU-P_xH_y adhesives bonded on stainless steel substrates.

Samples	Lap shear strength (MPa)	Work of debonding (N m^{-1})
PU-P_4H_0	8.72 ± 0.83	14 343
PU-P_3H_1	15.11 ± 1.95	26 932
PU-P_2H_2	17.16 ± 1.58	29 876
PU-P_1H_3	21.24 ± 1.10	35 731

Supplementary Table 10 | Comparison of lap shear strength and work of debonding between PU-P_1H_3 adhesive and reported advanced adhesives.

Type	Lap shear strength (MPa)	Work of debonding (N m^{-1})	Ref.
Strong Tough	**21.24**	**35 731**	**Our work**
	30.60	23 600	10
	18.52	29 692	11
	20.50	27 034[a)]	12
Strong Brittle	23.08	4330[a)]	13
	27.70	1258	14
	22.30	6020[a)]	15
	19.40	6990[a)]	16
	21.15	10 129[a)]	17
	20.67	9530	18
Ductile	15.20	11 623	19
	7.1 (70 °C)	6510	20
	12.10	13 225	21
	11.37	10 320	22
	7.39	12 501[a)]	23
	3.60[a)]	10 801[a)]	24
	7.24	8145	25
	10.00	7559[a)]	26
	6.43	7603[a)]	27
	10.92	7110	28
	10.20	6033	29
Fragile	6.02	3098[a)]	30
	7.80	1528[a)]	31
	11.51	3900[a)]	2
	10.75	1541	32
	5.71	3757[a)]	33
	4.43[a)]	3507[a)]	34
	2.26	808[a)]	35

[a)] indicates data estimated from figures in the literature.

Supplementary Table 11 | Comprehensive performance comparison of PU-P_1H_3 adhesive with advanced adhesives previously reported.

Ref.	Tensile strength (MPa)	Tensile toughness (MJ/m^3)	Lap shear strength (MPa)	Work of debonding (N m^{-1})	High temperature tolerance (°C)
**Our work**	**71.83**	**315.7**	**21.24 (Steel)**	**35 731**	**85**
30	/	/	6.02 (Steel)	3126[a)]	70
31	/	/	7.80 (Fe)	1528[a)]	/
36	67.68	4.6	16.74 (Steel)	5662[a)]	50
37	19.71[a)]	82.2[a)]	6.80 (Al)	/	/
19	32.60	68.1	15.20 (Steel)	11 623	50[b)]
2	22.80	64.8	11.51 (Steel)	3900[a)]	85
38	/	/	6.95 (Steel)	6076	50
39	/	/	15.60 (Ceramic)	/	100
20	2.32[c)]	4.2[c)]	7.10 (Brass)[d)]	6510	85
11	45.50	119.2	18.52 (Steel)	29 692	100
21	23.20	79.5	12.10 (Steel)	13 225	100
22	24.13	67.8	11.37 (Steel)	10 320	45
26	32.00	62.6	10.00 (Steel)	7559[a)]	95

Ref.	Low temperature tolerance (°C)	Adhesive strength at low temperature (MPa)	Solvent tolerance (type)	Adhesion strength after 3 recycles (MPa)	Substrate species (type)
**Our work**	**-196**	**17.18**	**11**	**20.23**	**7**
30	/	/	/	5.56	4
31	-10	3.70	4	7.60	8
36	-196	10.63[a)]	/	14.12[a)]	5
37	-196	4.87[a)]	/	5.89[a)]	6
19	0[b)]	15.10	5	8.86[a)]	8
2	-196	9.57	/	3.88	5
38	-80	0.42	6	4.94[a)]	10
39	-196	10.00	8	11.80[a)]	10
20	-5	2.04[a)]	/	5.20[a)]	8
11	-196	16.20	10	22.47	6

21	-70	10.60	9	11.30[a)]	6
22	-40	9.24	/	11.44	6
26	/	/	5	7.22 (Al)[a)]	3

[a)] indicates data estimated from figures in the literature.

[b)] indicates data measured in an underwater environment.

[c)] indicates data measured at 55 °C.

[d)] indicates data measured at 70 °C.

Supplementary References

1. Lai, W., Qin, B., Cao, X., Chen, Q., Xu, J.-F. & Zhang, X. Supertough and multirecyclable cross-linked polyurethane enabled by supramolecular chain extenders and noncovalent cross-linkers. *J. Am. Chem. Soc.* **147**, 29517-29525 (2025).
2. Qian, B. et al. Synergistic dual-dynamic covalent bonds enable robust, harsh environment-resistance, and structure evolution self-monitoring polyurethane adhesives. *Adv. Funct. Mater.* **36**, e09916 (2026).
3. Liu, Z. L. et al. Dynamic covalent polyurethane surface activation enabled stretchable, tough-adhesive, and conductive interface for soft_rigid hybrid electronics. *Nano Res.* **18**, 94907615 (2025).
4. Wang, Y. et al. Bone-inspired stress-gaining elastomer enabled by dynamic molecular locking. *Sci. Adv.* **10**, eadk5177 (2024).
5. Feng, H. et al. Ultratough yet dynamic crystalline poly(thiourethane) network directly from low viscosity precursors. *CCS Chem.* **6**, 682-692 (2024).
6. Fu, D. H. et al. Acylsemicarbazide moieties with dynamic reversibility and multiple hydrogen bonding for transparent, high modulus, and malleable polymers. *Macromolecules* **53**, 7914-7924 (2020).
7. Fan, C.-J. et al. Adaptable strategy to fabricate self-healable and reprocessable poly(thiourethane-urethane) elastomers via reversible thiol–isocyanate click chemistry. *Macromolecules* **53**, 4284-4293 (2020).
8. Zhang, L. et al. A highly efficient self-healing elastomer with unprecedented mechanical properties. *Adv. Mater.* **31**, 1901402 (2019).
9. Liu, W.-X., Zhang, C., Zhang, H., Zhao, N., Yu, Z.-X. & Xu, J. Oxime-based and catalyst-free dynamic covalent polyurethanes. *J. Am. Chem. Soc.* **139**, 8678-8684 (2017).

10. Lu, G. et al. Solution-sheared supramolecular oligomers with enhanced thermal resistance in interfacial adhesion and bulk cohesion. *Nat. Commun.* **16**, 7754 (2025).
11. Fu, Y. B. et al. A hierarchical energy dissipated structure enabled strong, ultra-tough, and sustainable adhesives. *Adv. Funct. Mater.* **34**, 2314561 (2024).
12. Zheng, H., Chen, S., Fu, Y., Chen, X., Zhang, G. & Liu, L. Robust dynamic epoxy adhesives with high environmental tolerance enabled by the coordination of small-molecule coupling agents with boroxine. *ACS Appl. Polym. Mater.* **5**, 9346-9353 (2023).
13. Huo, K. et al. Amino acid-induced dynamic networks of interface-enriched adhesive molecules for high-strength, recyclable, and substrate-adaptive protein adhesives. *Adv. Mater.* **38**, e14907 (2026).
14. Zhou, H. et al. Reversible and ultrastrong epoxy-polyurethane adhesives with microphase separation. *Adv. Funct. Mater.* **35**, 2506419 (2025).
15. Zhao, K. et al. Molecular engineered crown-ether-protein with strong adhesion over a wide temperature range from -196 to 200 °C. *Angew. Chem. Int. Ed.* **61**, e202207425 (2022).
16. Wei, Y., Mei, Y., Wu, M., Chen, S. & Liu, L. Electrically detaching behavior and mechanism of ionic conductive adhesives. *Chin. J. Polym. Sci.* **41**, 1142-1154 (2023).
17. Wu, M., Chen, S., Mei, Y., Liu, L. & Wei, Y. Interfacial electrochemistry-induced detachable adhesives with ultra-high bonding strength and detaching efficiency. *ACS Appl. Mater. Interfaces* **14**, 41456-41467 (2022).
18. Zhang, P. L., Zhang, B., Pan, J. S., Zhang, G. L., Ma, C. F. & Zhang, G. Z. Ultrastrong and versatile nonisocyanate polyurethane adhesive under extreme conditions. *Chem. Mater.* **35**, 7730-7740 (2023).
19. Xu, Y. C., Zou, S. L., Xu, C. L., Xiao, L. P. & Sun, R. C. Dual-network cellulose adhesives via copper coordination: Bridging ultrahigh adhesive performance and environmental stability. *Adv. Funct. Mater.* **35**, e11048 (2025).

20. Wang, Y. M. et al. Mechanically interlocked [an]daisy chain adhesives with simultaneously enhanced interfacial adhesion and cohesion. *Angew. Chem. Int. Ed.* **63**, e202409705 (2024).

21. Wang, H. S. et al. A furan-containing biomimetic multiphase structure for strong and supertough sustainable adhesives. *Cell Rep. Phys. Sci.* **4**, 101374 (2023).

22. Li, C. L. et al. A strain-reinforcing elastomer adhesive with superior adhesive strength and toughness. *Mater. Horiz.* **10**, 4183-4191 (2023).

23. Wu, Z. et al. Robust, self-healing, and multi-use poly(urethane-urea-imide) elastomer as a durable adhesive for thermal interface materials. *Small* **20**, 2401815 (2024).

24. Liu, J. et al. Multiple non-covalent interactions for mechanically robust and electrically detachable liquid-free poly(ionic liquids) ionoadhesives. *Chem. Eng. J.* **491**, 151967 (2024).

25. Yao, Y., Xu, Z. Y., Liu, B., Xiao, M., Yang, J. H. & Liu, W. G. Multiple h-bonding chain extender-based ultrastiff thermoplastic polyurethanes with autonomous self-healability, solvent-free adhesiveness, and aie fluorescence. *Adv. Funct. Mater.* **31**, 2006944 (2021).

26. Rahman, M. A. et al. Design of tough adhesive from commodity thermoplastics through dynamic crosslinking. *Sci. Adv.* **7**, eabk2451 (2021).

27. Fu, C. et al. Reprocessable supramolecular polymer adhesives for on-demand adhesion in multiple scenarios. *Mater. Horiz.* **12**, 9173-9184 (2025).

28. Sun, S., Liu, J., Yue, Q., Lv, J., Wang, S. & Wei, Y. Charge delocalization for electrically detachable poly(ionic liquids) ionoadhesives with ultrahigh mechanical robustness. *Macromolecules* **57**, 9355-9366 (2024).

29. Sun, P., Li, Y. Q., Qin, B., Xu, J. F. & Zhang, X. Super strong and multi-reusable supramolecular epoxy hot melt adhesives. *ACS Mater. Lett.* **3**, 1003-1009 (2021).

30. Lv, J. et al. Reversible biobased adhesives enable closed-loop engineered composites. *Nat. Commun.* **16**, 7871 (2025).

31. Gao, X., Fang, X., Wang, H., Tian, N. & Sun, J. Fully erasable amphibious adhesives derived from soybean oil with record-high underwater adhesion strength. *Adv. Mater.* **37**, e07894 (2025).
32. Chen, S. W., Lu, P., Zhao, Z. Y., Deng, C. & Wang, Y. Z. Recyclable strong and tough polyamide adhesives via noncovalent interactions combined with energy-dissipating soft segments. *Chem. Eng. J.* **446**, 137304 (2022).
33. Wang, S. et al. Strong, detachable, and self-healing dynamic crosslinked hot melt polyurethane adhesive. *Mater. Chem. Front.* **3**, 1833-1839 (2019).
34. Zhu, Q. et al. Sustainable snail-inspired bio-based adhesives with ultra-high adhesion. *Adv. Funct. Mater.* **34**, 2402734 (2024).
35. Zhou, Y. et al. Tough, waterproofing, and sustainable bio-adhesive inspired by the dragonfly wing. *Adv. Funct. Mater.* **34**, 2406557 (2024).
36. Zhou, Y. P. et al. Ultra-robust and tough epoxy resin enabled by pulley mechanism-based curing agent for strong and reversible adhesion. *Adv. Funct. Mater.* **35**, e17052 (2025).
37. Zhao, Y. et al. Chemically recyclable and tunable polyolefin-like multiblock copolymer adhesives. *Angew. Chem. Int. Ed.* **64**, e202513286 (2025).
38. Wang, S. et al. Dual-terminal molecular strategy for robust and reversible supramolecular adhesion. *Adv. Sci.* **12**, e11818 (2025).
39. Zhang, J. et al. Concentration-induced spontaneous polymerization of protic ionic liquids for efficient in situ adhesion. *Nat. Commun.* **15**, 4265 (2024).
