# Supplementary material for: A multiscale structure enabled strong, ultra-tough and sustainable polyurethane adhesives
Source: Nat Commun. 2026 May 6;17:6091. doi: 10.1038/s41467-026-72658-4 (PMC13385818; doi:10.1038/s41467-026-72658-4)
Supplement: Supplementary file 2 — Transparent Peer Review file [file 41467_2026_72658_MOESM2_ESM.pdf]

nature portfolio

Peer Review File

A multiscale structure enabled strong, ultra-tough and sustainable polyurethane adhesives

Corresponding Author: Professor Lan Liu

This file contains all reviewer reports in order by version, followed by all author rebuttals in order by version.

Version 0:

Reviewer comments:

Reviewer #1

(Remarks to the Author)
This manuscript reports a polyurethane adhesive with impressive performance, including high strength, ultra-high toughness, recyclability, and excellent stability under various environments. The experimental work is systematic and the characterization is solid. However, the conceptual novelty is incremental, as the core strategy of "top-down multiscale engineering" and "mismatched supramolecular interactions" have been extensively explored. The major advance of this work appears to be the integration of these known concepts into a single system, resulting in excellent but not paradigm-shifting properties.

1. The comparison in Figure 3h is a good start, but should be expanded. Some recent key work is lacking.

2. The adhesion mechanism is not very clear. The central claim of synergistically enhanced bulk and interfacial adhesion lacks direct proof. The lap shear test is a composite measurement that cannot decouple these two contributions. To validate the mechanism, direct measurements of interfacial fracture toughness and interfacial fatigue threshold are essential.

3. The spatial distribution of the various hard domains (from MMSIs, PCL, or HDI) and specific non-covalent interactions (cation-π, metal-oxygen binding) the adhesive-substrate interface remains speculative and warrants further investigation.

4. The direct evidence of the failure mode is lacking. Can you provide photographic documentation (macro and/or micro) of the failure surfaces after lap-shear testing?

5. The retention of high adhesive strength is shown at -196°C, which is far from the glass transition. The authors should provide a mechanistic explanation for this exceptional low-temperature performance. Does the polymer maintain its toughness at -196°C? Characterization of the bulk mechanical behavior and fracture mechanics at cryogenic temperatures is necessary to understand this phenomenon.

6. For a material targeting demanding applications, data on creep resistance and fatigue performance under cyclic loading are critical to assess long-term reliability and are currently absent.

7. To bridge the gap between laboratory performance and real-world utility, the authors are encouraged to demonstrate the adhesive's use in a prototype device or a simulated real-world scenario.

Reviewer #2

(Remarks to the Author)
The authors report a polyurethane adhesive that exhibits high adhesive strength and ultra-high work of debonding. The work integrates a dual-length dynamic crosslinked network with hierarchical organizations. The manuscript presents an amount of characterization data, and the reported numerical values for bulk mechanical properties are impressive. However, after a careful review, I have concerns regarding the fundamental design philosophy and the practical interpretation of the data. The manuscript appears to be a collection of modular characterizations where the connection between the high numerical values and actual adhesive utility is not fully justified. The study emphasizes bulk mechanical properties, while the critical analysis of interfacial failure mechanisms seems secondary. It appears that the high adhesive performance is merely a derivative of

the high bulk tensile strength, rather than a result of specific interfacial engineering. To improve the scientific rigor and practical relevance of this work, significant revisions are required. The authors should address the following points:
1. The manuscript claims to simultaneously optimize bulk cohesion and interfacial adhesion. However, the results suggest that the performance is dominated by the bulk mechanics of the material. It appears the failure mode is primarily cohesive failure rather than adhesive failure, yet the interfacial adhesion mechanism is rarely discussed.
2. The substrate scope is limited, and there is little evidence showing how the multiscale structure specifically improves wetting or interfacial interaction distinct from bulk reinforcement. The authors should explain if the adhesive strength is simply controlled by the bulk modulus or strength.
3. The authors highlight the ultra-high work of debonding (35,731 N m^{-1}) as a major advantage, but this value is derived from massive elongation (displacement > 3.5 mm). Typical structural adhesives fracture at much lower displacements (e.g., ~0.8 mm) to maintain structural rigidity. A joint that undergoes millimeter-scale deformation under load may compromise the assembly's integrity or suffer from creep at low stress levels. Is this high ductility practically useful for an adhesive, or is it simply a high numerical value derived from the material's elastomeric nature? Are there any specific applications that require such extreme compliance?
4. The authors rely on BDS data at -90 °C to explain beta/gamma relaxations. However, adhesive performance is evaluated at room temperature. According to time-temperature superposition, relaxations at -90 °C correspond to extremely high frequencies at room temperature. The authors should justify how these specific molecular motions at cryogenic temperatures contribute to quasi-static adhesive strength at service temperatures.
5. What is the specific rationale for using HMDI for the prepolymer while using HDI for the short-chain crosslinking? Additionally, why was 140 °C chosen for hot pressing? How would the performance change if hot pressing were conducted at higher or lower temperatures?
6. Conceptual and terminological accuracy:
-"Top-Down" Strategy: The authors describe their synthesis as "top-down." In materials science, synthesizing polymers from monomers is a classic "bottom-up" approach. This terminology is misleading.
-"Cross-linked Long Chain": The authors refer to the prepolymer as a "cross-linked long chain." However, the prepolymer appears to be linear at that stage.

Reviewer #3

(Remarks to the Author)
The authors design a polyurethane adhesive with multi-scale architecture that integrates dual-length dynamic crosslinks. This strategy successfully combines thermoplastic and thermosetting characteristics, achieving an balance between high strength, toughness, environmental stability, and recyclability. Overall this is a nice and comprehensive work. I would like to recommend it to be published after addressing the following points.
1. How about the quantitative contribution of each hierarchical structure across multiple length scales to the adhesive strength and work of debonding?
2. The authors present the temperature-dependent dynamics of hydrogen bonding. Will it be feasible to conduct similar investigations into the thermal evolution of other hierarchical structures such as mesoscale PCL crystallite and nanoscale MMSI hard domain. How about the temperatures where each hierarchical structure begins to disintegrate?
3. The dynamic phenol-carbamate bonds are key design of this adhesive. Relevant references are recommend to be cited. E.g. Science Bulletin 2024 , 69(12), 1875-1886. Science China Materials 2022, 65(9), 2553–2564. Chemical Engineering Journal 2022, 430(4), 133081.
4. Figure 2h illustrates strain-induced crystallization. The stretching direction is recommended to provide.
5. Is the strain-induced crystallization (SIC) of PCL reversible after multiple loading cycles? Have fatigue or cyclic tensile tests been performed to confirm the durability of SIC?
6. Could the authors supplement the data for the heat resistance with a rheological temperature sweep to pinpoint the solid-liquid transition temperature (G’ = G’’) of the materials?
7. The adhesive exhibits superior bonding to polar substrates. Its performance on non-polar surfaces such as polypropylene (PP), polyethylene (PE), or polytetrafluoroethylene (PTFE) are recommend to be explored.
8. What is the mass recovery efficiency of the adhesive following each recycling route (chemical dissolution and hot-press reprocessing)?

Version 1:

Reviewer comments:

Reviewer #1

(Remarks to the Author)
The authors have made considerable efforts to revise the manuscript and address the reviewers' comments. However, upon careful re-evaluation, several fundamental concerns remain inadequately resolved. These pertain to the conceptual novelty of the work, the rigor of the structural characterization, the directness of the evidence for interfacial interactions, and the reliability of the key mechanical performance data.

Major Concerns:

1. Incremental Novelty and Overlap with Recent Literature: In my initial assessment, I noted that the conceptual advance of this work appears incremental. The authors respond by framing their innovation as "the successful integration of known

concepts into the thermosetting polyurethane systems." This argument, however, is substantially undermined by the existence of a highly relevant and recently published article in Nature Communications (Lu, G. et al. Nat. Commun. 2025, 16, 7754). Both studies address the central challenge of synergistically enhancing bulk cohesion and interfacial adhesion through multiscale structural design. To establish a distinct contribution, the authors must clearly articulate the essential scientific differentiation between their work and this published study. Claiming novelty based solely on a different material platform (thermoset vs. thermoplastic/supramolecular) is, in this context, insufficient.

2. Ambiguity in Structural Differentiation: A critical mechanistic question remains unaddressed: Does the crystallization of the PCL chains influence the microphase separation scale or the ordering of the MMSIs hard domains within the original PU-P_1H_3 sample? The potential interplay between these two structural elements requires explicit investigation to validate the proposed decoupled contributions to bulk cohesion and interfacial adhesion.

3. Lack of Direct Chemical Evidence for Interfacial Bonding: The authors have supplemented their manuscript with DFT simulations and AFM adhesion force mapping. While these techniques provide useful indirect probes, they do not constitute direct chemical evidence for the proposed interfacial interactions (e.g., hydrogen bonding, cation-π interaction, metal-ion multiple oxygen binding). More definitive spectroscopic evidence at the interface is necessary to substantiate these claims.

4. Insufficient Experimental Details for Key Mechanical Tests: The inclusion of interfacial fracture toughness and fatigue threshold data is a positive addition. However, the experimental details provided are insufficient to assess the reliability of these critical measurements. A comprehensive description of the testing protocols is essential for the data to be properly evaluated.

Reviewer #2

(Remarks to the Author)
The authors have significantly improved the manuscript and provided substantial new data. However, a few critical points regarding the underlying mechanism and terminology require further clarification.
1. Clarification of the Bulk-Interface Relationship
A logical gap persists in the interpretation of the adhesion mechanism. Previous results indicated that the lap shear strength correlates strongly with the bulk tensile strength. However, the optical images show a clean interfacial failure (Supplementary Fig. 25), which typically indicates that the interfacial interaction is the performance-limiting factor. Furthermore, Fig. 5e shows that the adhesion force of PU-P1H3 is only moderate. These observations strongly suggest that interfacial adhesion chemistry may not be the dominant factor driving the macroscopic performance. The authors should clarify which factor is the ultimate determinant of the lap shear limit in this system: the bulk yield strength or the interfacial bonding strength? A more systematic discussion on this relationship would significantly improve the scientific impact of the work.
2. Additional Data for PPG Control
While surface adhesion data for the PPG-based adhesive were provided, please supplement its bulk tensile stress-strain curves as well. This is necessary to determine if its mixed failure mode stems from interfacial effects or simply a consequence of reduced bulk cohesive strength.
3. Terminology: "Top-Down" Design
In materials science, "top-down" (e.g., lithography) and "bottom-up" (e.g., molecular synthesis) have well-established, distinct definitions. The authors are encouraged to re-examine these standard definitions and reconsider if "top-down" accurately describes a molecular-scale synthesis and assembly process.

Reviewer #3

(Remarks to the Author)
The previous comments have been addressed.

Version 2:

Reviewer comments:

Reviewer #1

(Remarks to the Author)
The authors have made substantial efforts to revise the manuscript and address previous concerns, and the overall quality of the work has been significantly improved. However, before final acceptance, I would like to offer a few final suggestions to further strengthen the contextualization and completeness of the manuscript.
1. Differentiation from a closely related work in Introduction
In the rebuttal letter, the authors provided a detailed point-by-point differentiation between their work and a recently published Nature Communications article by Lu et al. (Nat. Commun. 2025, 16, 7754). To better guide readers and clearly establish the conceptual novelty of the present study, I strongly recommend that the authors incorporate this critical comparison directly into the Introduction section of the main manuscript. Specifically, the authors should highlight how their thermosetting dual-length dynamic crosslinked network approach fundamentally differs from the solution shear-induced alignment strategy used in Lu et al.'s supramolecular oligomer system, particularly regarding how each platform addresses

the central challenge of simultaneously enhancing interfacial adhesion and bulk cohesion.

2. Missing important references in the second paragraph of Introduction
In the second paragraph of the Introduction, when discussing molecular design strategies for simultaneously improving the strength and toughness of adhesives, several important recent contributions have been overlooked. The authors should cite the following relevant works to provide a more comprehensive literature background:
Science Advances 2025, 11, eaea3097
Advanced Functional Materials 2023, 33, 2306914
ACS Applied Materials & Interfaces 2023, 15, 44194–44204
These studies present complementary approaches to addressing the strength-toughness dilemma in polymeric adhesives and deserve appropriate acknowledgment in the revised manuscript.

Given the overall novelty of the multiscale design concept, the impressive performance metrics, and the comprehensive set of characterization data provided, the manuscript is suitable for publication pending the incorporation of the above minor revisions.

Reviewer #2

(Remarks to the Author)
The revised manuscript can be accepted.

Open Access This Peer Review File is licensed under a Creative Commons Attribution 4.0 International License, which permits use, sharing, adaptation, distribution and reproduction in any medium or format, as long as you give appropriate credit to the original author(s) and the source, provide a link to the Creative Commons license, and indicate if changes were made.
In cases where reviewers are anonymous, credit should be given to 'Anonymous Referee' and the source.
The images or other third party material in this Peer Review File are included in the article's Creative Commons license, unless indicated otherwise in a credit line to the material. If material is not included in the article's Creative Commons license and your intended use is not permitted by statutory regulation or exceeds the permitted use, you will need to obtain permission directly from the copyright holder.
To view a copy of this license, visit https://creativecommons.org/licenses/by/4.0/

Responses to Reviewers' Comments

Reviewer #1: This manuscript reports a polyurethane adhesive with impressive performance, including high strength, ultra-high toughness, recyclability, and excellent stability under various environments. The experimental work is systematic and the characterization is solid. However, the conceptual novelty is incremental, as the core strategy of "top-down multiscale engineering" and "mismatched supramolecular interactions" have been extensively explored. The major advance of this work appears to be the integration of these known concepts into a single system, resulting in excellent but not paradigm-shifting properties.

General Response: We sincerely appreciate the reviewer for reviewing the manuscript and giving valuable comments and constructive comments. All significant issues have been carefully considered and addressed, with all changes in the revised manuscript marked in blue. A detailed point-to-point response to your valuable comments can be found below.

We thank you for your insightful comment, which allows us to clarify the core novelty of our work. We fully acknowledge that "top-down multiscale engineering and mismatched supramolecular interactions have been extensively explored" and "the integration of these known concepts into a single system". However, this has been exclusively within the context of thermoplastic polyurethane or supramolecular oligomer systems, as exemplified in *Nat Commun* 2025, 16, 7754; *Angew. Chem. Int. Ed.* 2023, 62, e202301762. The pivotal advance presented in our work is the creation of a fundamentally distinct class of crosslink network that, for the first time, enables the functionality and integration of these known concepts in thermosetting polyurethane systems. The prevailing view in this field has been that while conventional thermoset networks possess advantages in terms of cohesion, structural stability, and thermal stability, the constrained short chains between crosslinks severely inhibit those efficient strengthening and toughening strategies employed in thermoplastic polyurethane and supramolecular oligomer systems, such as crystallization, microphase separation, and hierarchical hydrogen bonds. By redesigning the fundamental thermoset framework and then constructing an ingenious dual-length crosslinked network, we have combined thermoplastic and thermosetting characteristics to overcome these challenges, thus paving the way for the effective application of these strengthening and toughening strategies in previously

incompatible thermoset polyurethane systems. Subsequently, based on the actual performance requirements of adhesives, we employed top-down design of molecular building blocks to achieve the integration of multi-scale organizations. Consequently, the core innovation lies not in those known concepts themselves, but the successful integration of them into the thermosetting polyurethane systems, achieved by designing a novel crosslink network that supports their functionality.

We have refined the narrative in the Introduction (Page 3-4, lines 69-71, 76-85) of the revised manuscript to more sharply articulate the core innovation of this work. All the revisions are marked in blue for reading.

(1) The comparison in Figure 3h is a good start, but should be expanded. Some recent key work is lacking.

Response: We are grateful to your valuable suggestion. In response, we have expanded the literature comparison by incorporating five recent high-impact studies that address key advances in adhesive performance (*Nat. Commun.* 2025, 16, 7754; *Nat. Commun.* 2025, 16, 7871; 2222; *Adv. Mater.* 2026, 38, e14907; *Adv. Mater.* 2025, 37, e07894; *Angew. Chem. Int. Ed.* 2024, 63, e202409705). These additions strengthen our benchmarking by covering critical developments in interfacial engineering, dynamic bonding, and network topology. The revised analysis (now 27 data points from primarily 2023-2025 high-quality reports) demonstrates the superior adhesive strength and work of debonding of our adhesive relative to advanced adhesives in recent literatures. All the revisions are added in the revised manuscript (Fig. 3h and Supplementary Table 10).

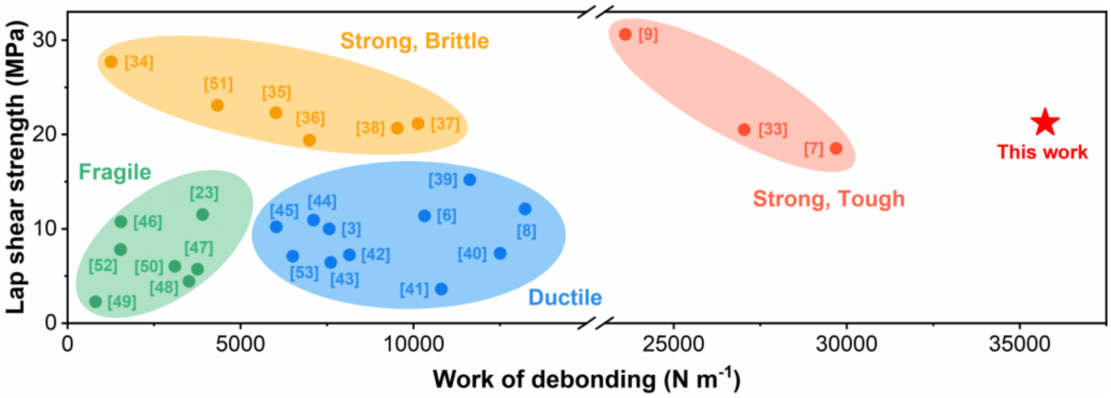

Fig. 3h | Comparison of lap shear strength and work of debonding between PU-P_1H_3 adhesive

and reported advanced adhesives.

(2) The adhesion mechanism is not very clear. The central claim of synergistically enhanced bulk and interfacial adhesion lacks direct proof. The lap shear test is a composite measurement that cannot decouple these two contributions. To validate the mechanism, direct measurements of interfacial fracture toughness and interfacial fatigue threshold are essential.

Response: We sincerely appreciate the reviewer's constructive comments regarding the interfacial interaction and its mechanism. Admittedly, while we demonstrated simultaneous optimization of both bulk cohesion and interfacial adhesion, the original manuscript primarily focused on the former, with comparatively limited characterization and mechanistic discussion of the latter. To address this gap, we have conducted a systematic investigation of interfacial adhesion and its mechanism as follows:

The interfacial adhesion mechanism is illustrated in Fig. 5a. During the hot pressing adhesion process, the MMSIs units with low relaxation time (see the BDS analysis for details, in the original manuscript) and PCL chain segments synergistically enhance interfacial adhesion through multiple non-covalent interactions (e.g., hydrogen bonding, cation-π interaction, metal-ion multiple oxygen binding). These interactions promote intimate contact between the adhesive and substrate, while partially achieving mechanical interlocks with microscopic interfacial gaps. Consequently, multiscale high energy phases can be in situ formed at the adhesive-substrate interface, comprising MMSIs hard domains and PCL crystals. They anchor at the interface and effectively hold the interfacial crack tip, thereby elevating the interfacial adhesion and fatigue threshold. We first employed density functional theory (DFT) simulations (Fig. 5b, c and Supplementary Fig. 23) to elucidate the interfacial adhesion mechanism, where mononuclear Fe(III) hydroxo-aqua complexes were utilized to characterize the hydroxyl-terminated iron substrate surface. Both interaction region indicator (IRI) scatter plots and isosurface maps clearly confirm that IPDH and DABA engage in the aforementioned non-covalent interactions with the substrate. To elucidate the respective contributions of MMSIs hard domains and PCL crystals to interfacial adhesion, two additional prepolymers were synthesized for decoupled comparison (Fig. 5d). The soft segment in prepolymer 1 (P1) was substituted with polypropylene glycol (PPG), whereas prepolymer 2 (P2) was synthesized

without MMSIs units, in contrast to the original prepolymer (P). Thus, the cured PU-$P1_1H_3$ adhesive contained only MMSIs hard domains, while PU-$P2_1H_3$ retained exclusively PCL crystals. Peak Force Quantitative Nanomechanical Mapping (PF QNM) was then employed to characterize the modulus and adhesion distributions across surface layers. As shown in Supplementary Fig. 24, PU-P_1H_3 exhibited higher and more broadly distributed surface modulus compared to PU-$P1_1H_3$ and PU-$P2_1H_3$, suggesting the accumulation of both high energy phases (MMSIs hard domains and PCL crystals) at its surface, achieved through multiscale design. Notably, the mean interfacial adhesion forces for PU-P_1H_3, PU-$P1_1H_3$, and PU-$P2_1H_3$ were quantified as 0.53, 0.80, and 0.22 μN, respectively (Fig. 5e), demonstrating that MMSIs hard domains play a dominant role in interfacial adhesion enhancement, while PCL crystals exhibit a weaker but non-negligible contribution. We further conducted lap shear tests (Fig. 5f). The PU-P_1H_3 adhesive, which combines high bulk cohesion and strong interfacial interactions, exhibited the highest adhesive strength and work of debonding. Due to the absence of PCL crystals, the PU-$P1_1H_3$ adhesive showed reduced bulk cohesion, leading to decreased adhesive strength (9.96 MPa) and work of debonding (8137 $N \cdot m^{-1}$). As for the PU-$P2_1H_3$ adhesive, the lack of MMSIs units substantially weakened interfacial interactions, resulting in significantly compromised adhesive properties. Besides, the strain-induced crystallization behavior of PU-P_1H_3 during lap shear test resulted in dynamic bulk reinforcement, enabling strategic stress redistribution that preferentially initiated atypical interfacial failure without residual adhesive (Supplementary Fig. 25). In contrast, PU-$P1_1H_3$ containing non-crystallizable PPG exhibited a predominantly mixed failure mode, while PU-$P2_1H_3$ with weaker interfacial interactions showed characteristic interfacial failure behavior. These differences highlight the distinct roles of hierarchical structure: PCL crystals primarily enhance bulk cohesion, while MMSIs hard domains are critical for improving interfacial interactions. The above analysis confirms that PU-P_xH_y adhesives form strong interactions with functional groups (e.g., hydroxyl and metal ions) on the substrate through surface-enriched non-covalent units such as ester, urea, amide, and phenyl groups. Then, the resulting multiscale high energy phases (MMSIs hard domains and PCL crystals) become densely anchored at the adhesive-substrate interface, leading to significantly enhanced interfacial adhesion of the adhesive coatings.

To further elucidate how the multiscale structures synergistically enhance both bulk cohesion

and interfacial adhesion, we performed 90° peel tests on stainless steel substrates to decouple and quantify both interfacial fracture toughness and fatigue threshold. As shown in Fig. 5g, PU-P_1H_3, PU-$P1_1H_3$, and PU-$P2_1H_3$ exhibited interfacial fracture toughness values of 4125, 913 and 2957 J m^{-2}, respectively, further evidencing the dominant role of interfacial MMSIs hard domains in adhesion enhancement. Besides, PU-P_1H_3 yielded superior interfacial fatigue threshold (Γ_0 = 1121 J m^{-2}) compared to both counterparts (Fig. 5h), demonstrating that co-anchored MMSIs hard domains and PCL crystals effectively hold the interfacial crack tip while dissipating energy. These quantitative metrics provide compelling support for the synergistic improvement of both bulk cohesion and interfacial adhesion through multiscale engineering.

All the revisions are added in the revised manuscript (Page 20-24, line 462-542) and marked in blue for reading.

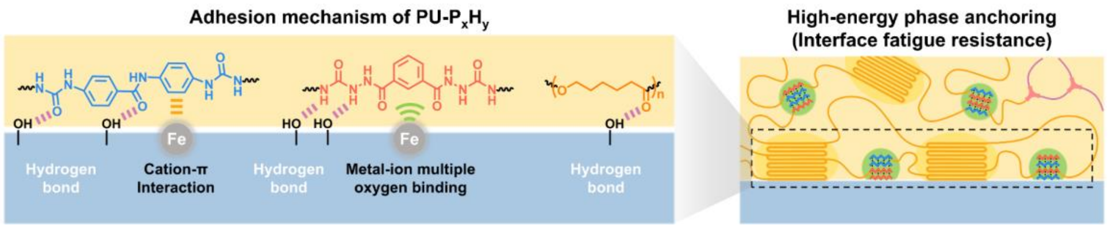

Fig. 5a | Interfacial interaction and fatigue-resistant interface adhesion mechanism.

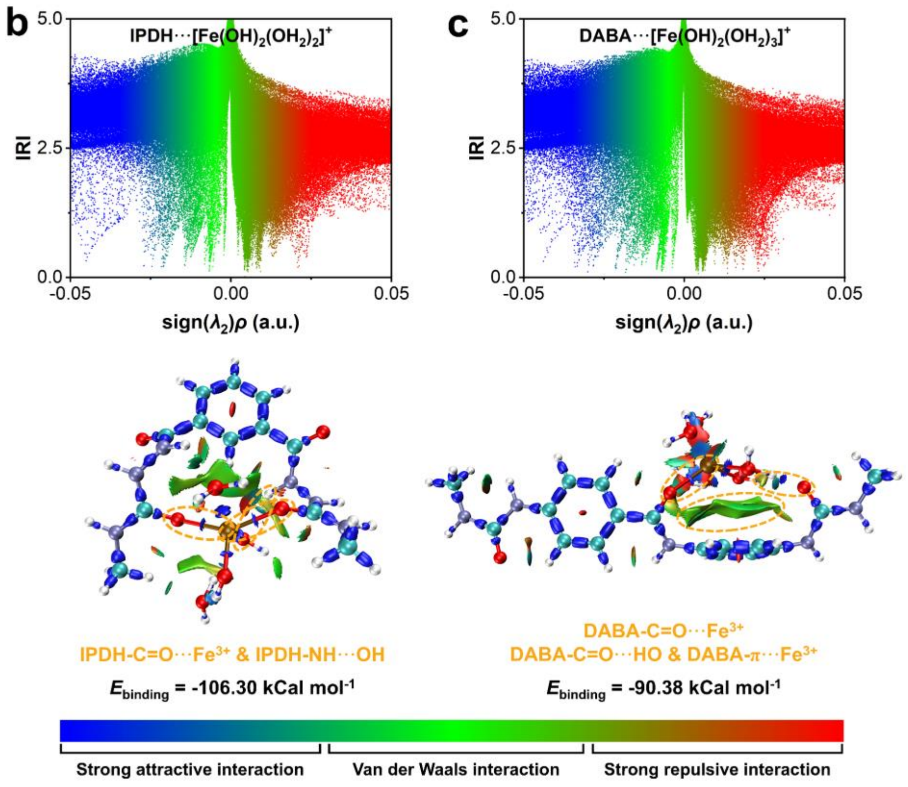

Fig. 5b-c | Plots of IRI versus sign(λ_2)ρ and the gradient isosurfaces for the complexes: **b**

IPDH···$[Fe(OH)_2(OH_2)_2]^+$, **c** DABA···$[Fe(OH)_2(OH_2)_3]^+$.

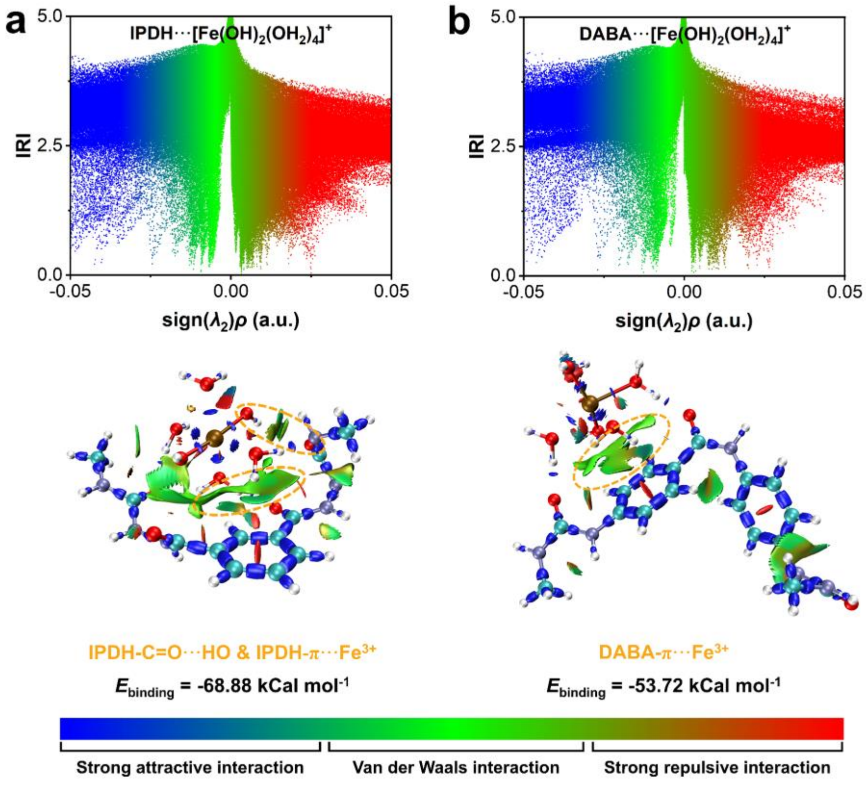

Supplementary Fig. 23 | Plots of IRI versus sign(λ_2)ρ and the gradient isosurfaces for the complexes: **a** IPDH···$[Fe(OH)_2(OH_2)_4]^+$, **b** DABA···$[Fe(OH)_2(OH_2)_4]^+$.

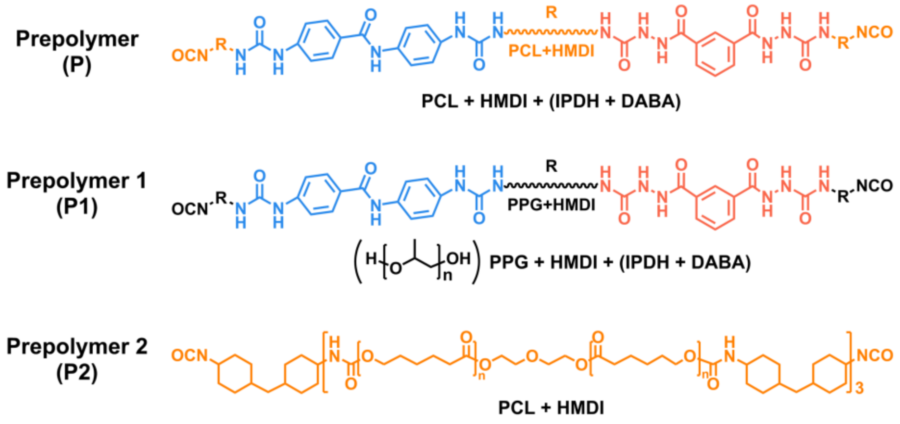

Fig. 5d | Structures of the three prepolymers, imparting distinct multi-scale organizations.

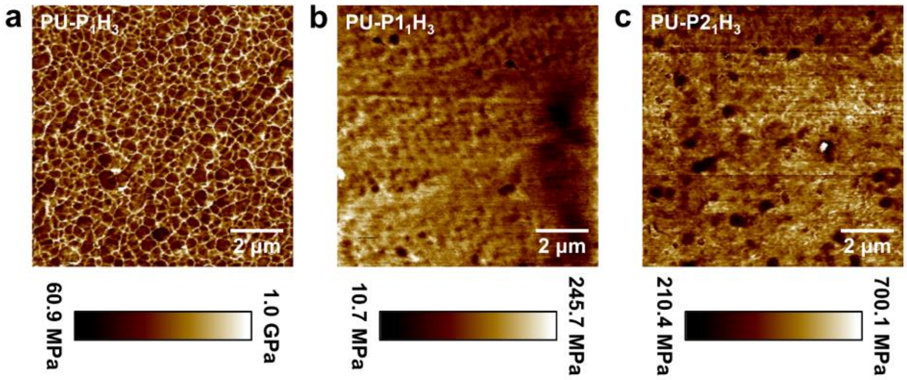

Supplementary Fig. 24 | AFM-based surface modulus measurements. a PU-P$_1$H$_3$, **b** PU-P1$_1$H$_3$, and **c** PU-P2$_1$H$_3$.

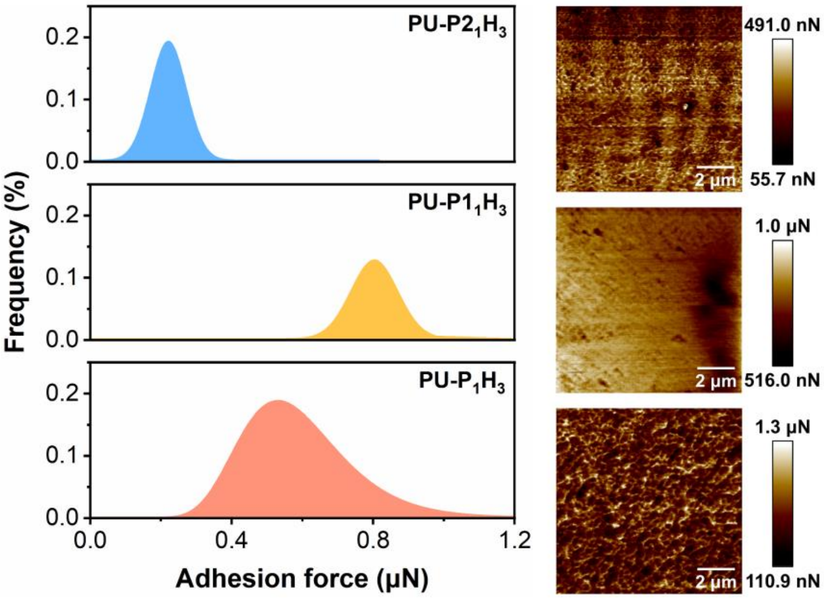

Fig. 5e | Adhesion force distribution maps for PU-P$_1$H$_3$, PU-P1$_1$H$_3$, and PU-P2$_1$H$_3$ adhesives.

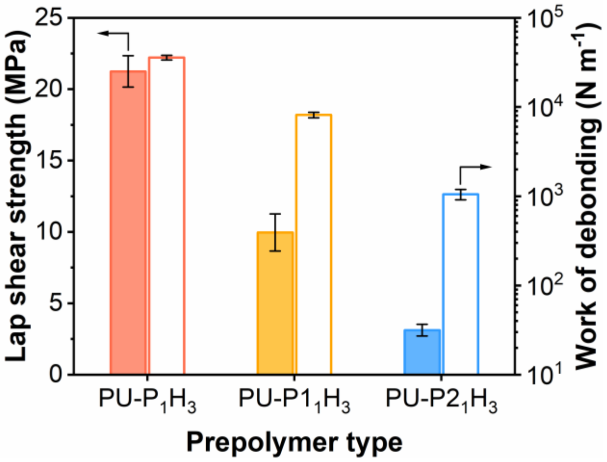

Fig. 5f | Comparison of the adhesion properties of PU-P$_1$H$_3$, PU-P1$_1$H$_3$, and PU-P2$_1$H$_3$

adhesives.

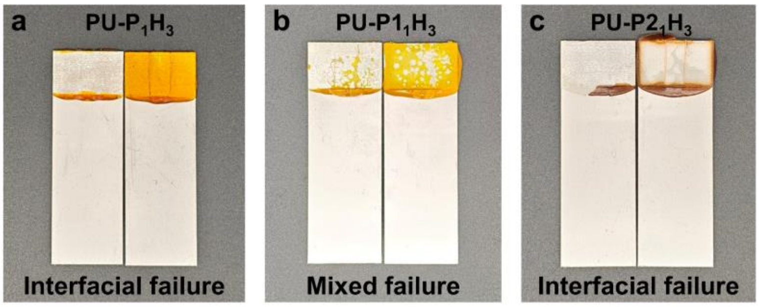

Supplementary Fig. 25 | Failure modes. a Interfacial failure (PU-P_1H_3), **b** mixed failure (PU-$P1_1H_3$), and **c** interfacial failure (PU-$P2_1H_3$).

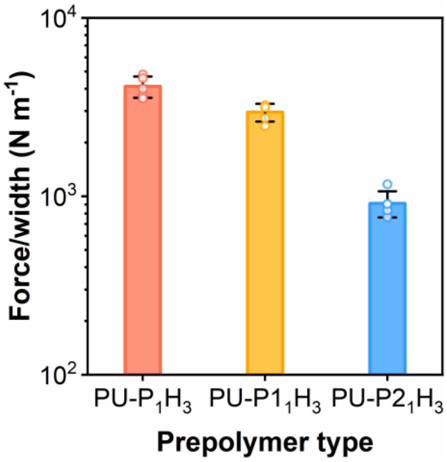

Fig. 5g | Interfacial fracture toughness for PU-P_1H_3, PU-$P1_1H_3$, and PU-$P2_1H_3$ on stainless steel substrates.

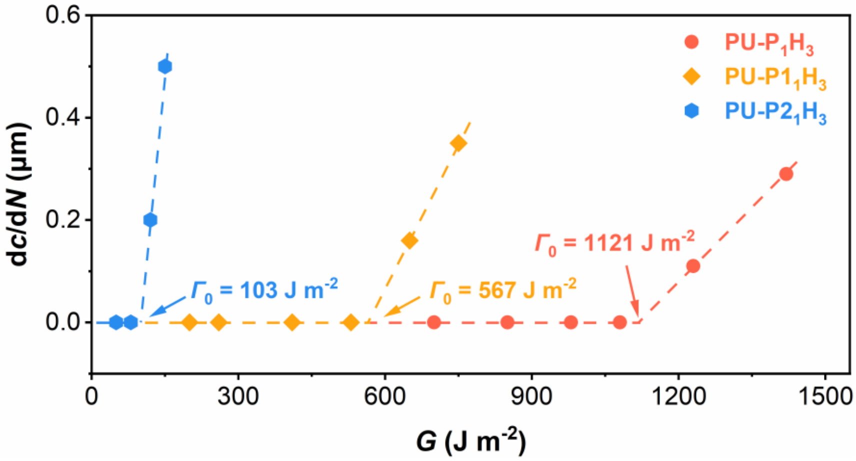

Fig. 5h | Interfacial fatigue threshold for PU-P_1H_3, PU-$P1_1H_3$, and PU-$P2_1H_3$ on stainless steel substrates.

(3) The spatial distribution of the various hard domains (from MMSIs, PCL, or HDI) and specific non-covalent interactions (cation-π, metal-oxygen binding) the adhesive-substrate interface remains speculative and warrants further investigation.

Response: Thank you for your insightful comment regarding the spatial distribution of hard domains and the evidence for specific non-covalent interactions at the adhesive-substrate interface. We have provided responses to each point as follows:

1) For the existence and spatial distribution of various hard domains, we have in fact provided rigorous characterization in the original manuscript. Specifically, the presence of PCL crystals was confirmed by wide-angle X-ray scattering (WAXS, Fig. 2g), while their spatial distribution was demonstrated via polarized optical microscopy (POM, Fig. 2h) images. Density functional theory (DFT) simulations revealed the propensity of MMSIs units to form multimers (Fig. 2d), and small-angle X-ray scattering (SAXS) further verified the existence and periodic spacing of MMSIs hard domains (Fig. 2f). Additionally, HDI serves as a molecular module for short crosslinked networks with restricted chain mobility, thereby inhibiting the formation of typical hard domains. Based on these comprehensive experimental and theoretical analyses, we believe the spatial distribution of hard domains is not merely speculative, but rather well-substantiated by the collected evidence.

2) For the specific interfacial non-covalent interactions, direct experimental characterization is challenging due to bond-interface limitations. Instead, we employed DFT simulations to provide theoretical evidence. Using mononuclear Fe(III) hydroxo-aqua complexes to model hydroxyl-terminated iron substrates (Fig. 5b, c, and Supplementary Fig. 23), we simulated their binding with MMSIs units (IPDH and DABA) and performed interaction region indicator (IRI) analysis. Both the IRI scatter plots and isosurfaces (marked by yellow dashed frames) clearly demonstrate hydrogen bonding, cation-π interaction, and metal-ion multiple oxygen binding. These results collectively confirm the existence of multiple non-covalent interactions at the adhesive-substrate interface, which are crucial for understanding the interfacial adhesion mechanism.

All the revisions are added in the revised manuscript (Page 21-22, line 487-491) and marked in blue for reading.

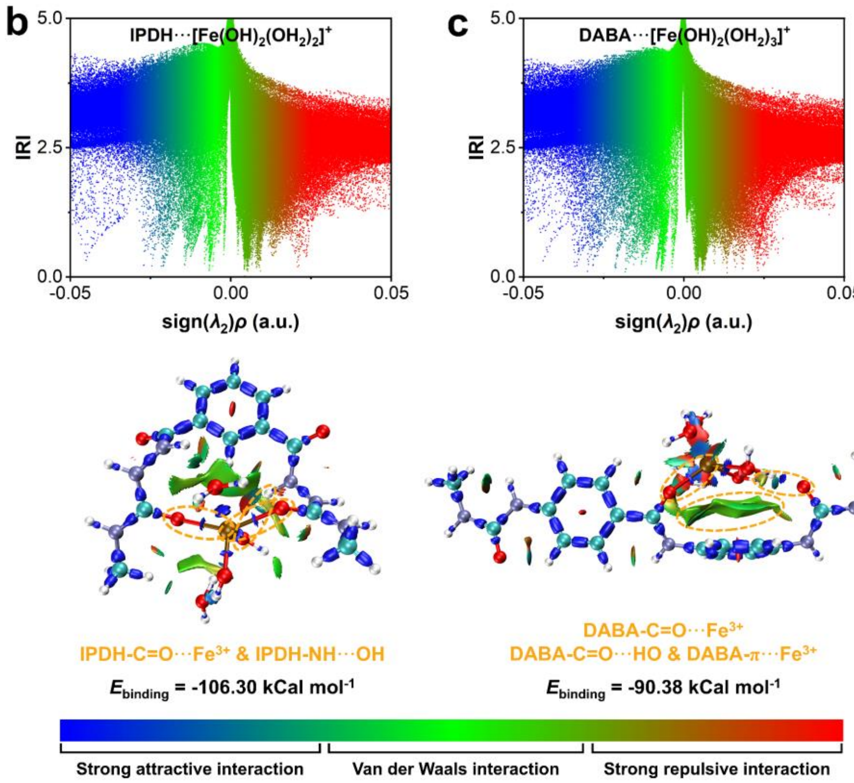

Fig. 5b-c | Plots of IRI versus sign(λ_2)ρ and the gradient isosurfaces for the complexes: **b** IPDH···$[Fe(OH)_2(OH_2)_2]^+$, **c** DABA···$[Fe(OH)_2(OH_2)_3]^+$.

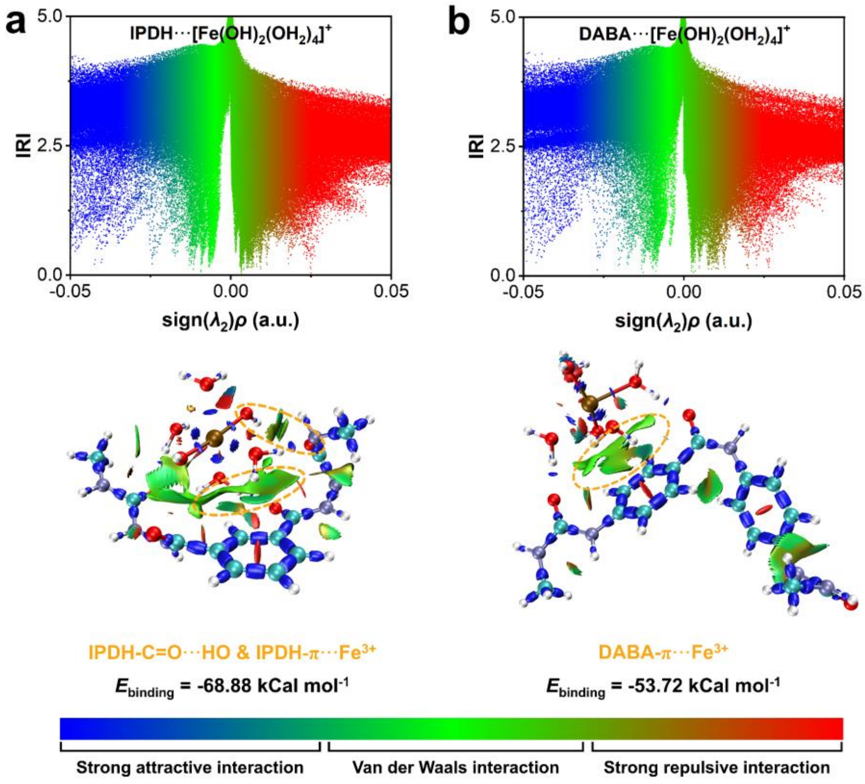

Supplementary Fig. 23 | Plots of IRI versus sign(λ_2)ρ and the gradient isosurfaces for the complexes: **a** IPDH···$[Fe(OH)_2(OH_2)_4]^+$, **b** DABA···$[Fe(OH)_2(OH_2)_4]^+$.

(4) The direct evidence of the failure mode is lacking. Can you provide photographic documentation (macro and/or micro) of the failure surfaces after lap-shear testing?

Response: We are grateful to the reviewer's constructive comment regarding the evidence of failure mode, which is essential for elucidating the coupling between bulk cohesion and interfacial adhesion. Here, we supplemented interfacial failure image of PU-P_1H_3 adhesive-bonded stainless steel joints (Supplementary Fig. 25). Notably, the interfacial failure in our work fundamentally differs from classical cases because interfacial interaction is comparable to bulk cohesion. Through deliberate design, this system simultaneously optimizes both bulk cohesion and interfacial adhesion to enhance adhesive performance. Remarkably, strain-induced crystallization during lap-shear dynamically strengthened the bulk material, strategically redirecting stress distribution to preferentially induce interfacial failure without adhesive residue. In contrast, when replacing the crystallizable PCL in PU-P_1H_3 with non-crystallizable polypropylene glycol (PPG), the resulting PU-$P1_1H_3$ adhesive (see comment 2 for details) exhibited a near-mixed failure mode. All the revisions are added in the revised manuscript (Page 22-23, line 511-516) and marked in blue for reading.

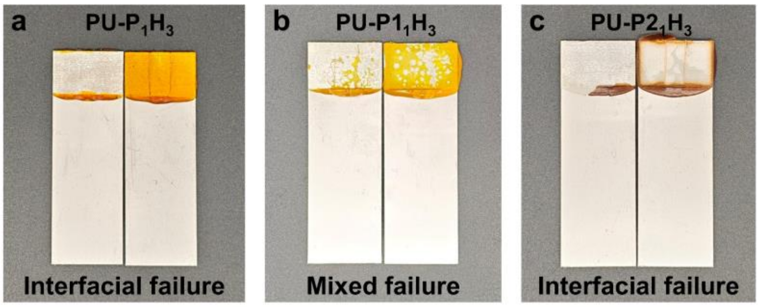

Supplementary Fig. 25 | Failure modes. a Interfacial failure (PU-P_1H_3), **b** mixed failure (PU-$P1_1H_3$), and **c** interfacial failure (PU-$P2_1H_3$).

(5) The retention of high adhesive strength is shown at -196 °C, which is far from the glass transition. The authors should provide a mechanistic explanation for this exceptional low-temperature performance. Does the polymer maintain its toughness at -196 °C? Characterization of the bulk mechanical behavior and fracture mechanics at cryogenic temperatures is necessary to understand this phenomenon.

Response: We are grateful to your insightful comment regarding the exceptional adhesive strength at cryogenic temperatures. The underlying mechanisms are elucidated as follows:

1) Lubrication effect of bound water. For conventional polymer adhesives, low temperatures freeze polymer chain mobility, leading to embrittlement and bond degradation. Notably, adhesives containing free water (e.g., hydrogels) suffer from ice cluster-induced defects at cryogenic temperatures, drastically reducing adhesive strength. In contrast, bound water demonstrates exceptional freeze resistance owing to its temperature-insensitive nature. It provides lubrication to prevent stress concentration or cracking, thereby enhancing cryogenic performance. The PU-P_xH_y adhesives contain abundant ester, amide, and urea groups that stabilize bound water via hydrogen bonds, while its balanced hydrophilic/hydrophobic chain segments design excludes free water infiltration. This bound water-mediated cryogenic chain mobility has been corroborated by *Angew. Chem. Int. Ed.* 2022, e202207425; *Chin. Chem. Lett.* 2023, 107830; *J. Mater. Chem. A* 2023, 6286; *J. Am. Chem. Soc.* 2020, 21522, etc.

2) Hierarchical hydrogen bonds broaden energy dissipation window while nanoscale microphase separation provides free volume. The urea, amide, urethane, ester, and benzene groups in PU-P_xH_y can form hydrogen bonds with varying configurations (single, dual, or multiple), exhibiting diverse dissociation/recombination timescales and broadly distributed binding energies. This hierarchical design enables sequential activation of different hydrogen bonds to dissipate energy under cryogenic conditions. Simultaneously, the nanoscale microphase separation of MMSIs units creates additional free volume, which not only restricts PCL crystallization at -196 °C but also facilitates the dynamic reorganization of hydrogen bond networks. These mechanisms have been experimentally validated by prior research, such as *Adv. Mater.* 2025, 2509421.

3) Decoupling between bulk toughness and adhesive strength. Admittedly, while the aforementioned mechanisms partially mitigate the toughness reduction of PU-P_xH_y, its ductility still extremely decreases at -196 °C, failing to retain the bulk toughness of room temperature. However, this does not directly correlate with its high adhesive strength at -196 °C, since adhesive strength depends on interfacial adhesion and bulk cohesion rather than bulk toughness. Interfacial adhesion of PU-P_xH_y relies on noncovalent interactions, including hydrogen bonding, cation-π interaction, metal-ion multiple oxygen binding. They stabilize or even strengthen at

cryogenic temperatures due to suppressed thermal disruption. As for bulk cohesion, the hard domains (PCL crystals and MMSIs microphases) maintain structural integrity at cryogenic temperatures, while amorphous PCL partially freezes. Consequently, PU-P_xH_y exhibits significantly increased Young's modulus (Fig. 6c, see the DMA data in Fig. 5c of the original manuscript) but reduced toughness, displaying a strong-yet-brittle characteristic (Supplementary Fig. 20). This allows it to withstand high stress at minimal strain, ensuring exceptional adhesive strength at -196 °C.

In summary, the exceptional adhesive strength at -196 °C arises from: (i) bound water lubrication, (ii) hierarchical hydrogen bonds broaden energy dissipation window, (iii) nanoscale microphase separation provides free volume, (iv) enhanced modulus, and (v) stable noncovalent interactions. All the revisions are added in the revised manuscript (Page 19, line 429-432) and marked in blue for reading.

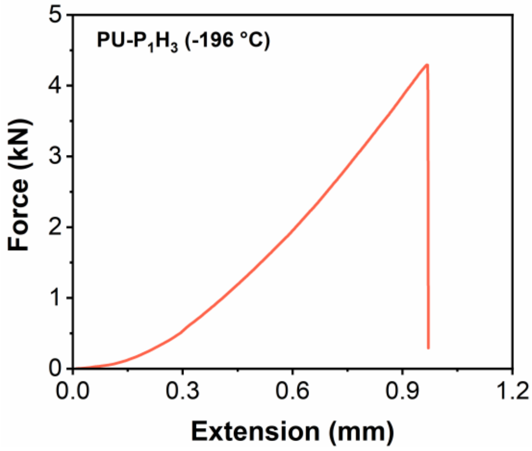

Supplementary Fig. 20 | Force-extension curve from lap shear test of PU-P_1H_3 adhesive at -196 °C.

(6) For a material targeting demanding applications, data on creep resistance and fatigue performance under cyclic loading are critical to assess long-term reliability and are currently absent.

Response: Thank you for your constructive comments regarding the long-term reliability. Here, we supplemented the creep test (Supplementary Fig. 16) according to suggestion, and conducted cyclic 90° peel tests to evaluate the interface fatigue threshold (Fig. 5h). The cyclic 90° peel tests are prioritized over bulk tensile fatigue tests for three reasons: (1) Adhesive joints

in real applications primarily experience peel stresses at edges where failure initiates, which differs fundamentally from uniform bulk tensile loading; (2) The yielding behavior of PU-P_xH_y would lead to misleading damage accumulation in conventional tensile cycles; (3) The cyclic 90° peel test directly measures the critical load (Γ_0), below which theoretically no delamination progresses even after infinite cycles, providing a more relevant durability criteria for adhesive applications. Similarly, we evaluated creep resistance using PU-P_1H_3 adhesive-bonded stainless steel joints rather than bulk films to better simulate real-world service conditions and provide practical performance data for engineering applications.

For the creep resistance, we have conducted a series of creep experiments under lap shear configuration at constant lap shear stress levels corresponding to 1, 2, 3, and 4 MPa (Supplementary Fig. 16). The results indicate that under these conditions, the creep strain all stabilized within 30 minutes, suggesting that the adhesive can maintain its creep within an acceptable range over extended periods under typical service loads, thereby ensuring dimensional stability. In addition, as a practical demonstration, PU-P_1H_3 adhesive-bonded stainless steel joints successfully supported a weight of 8 kg for 30 days without detectable creep or adhesive failure (Supplementary Fig. 15).

For the fatigue performance, we have performed cyclic 90° peel tests of PU-P_1H_3 on stainless steel substrates to quantify interfacial fatigue threshold (Fig. 5h). Attributed to the co-anchored MMSIs hard domains and PCL crystals that effectively hold the interfacial crack tip while dissipating energy, PU-P_1H_3 exhibited a superior interfacial fatigue threshold (Γ_0 = 1121 J m^{-2}) compared to its counterparts (see comment 2 for details), demonstrating exceptional resistance to cyclic delamination, a critical performance metric for adhesive durability under long-term service conditions.

All the revisions are added in the revised manuscript (Page 14, line 318-321, and page 23, line 529-532) and marked in blue for reading.

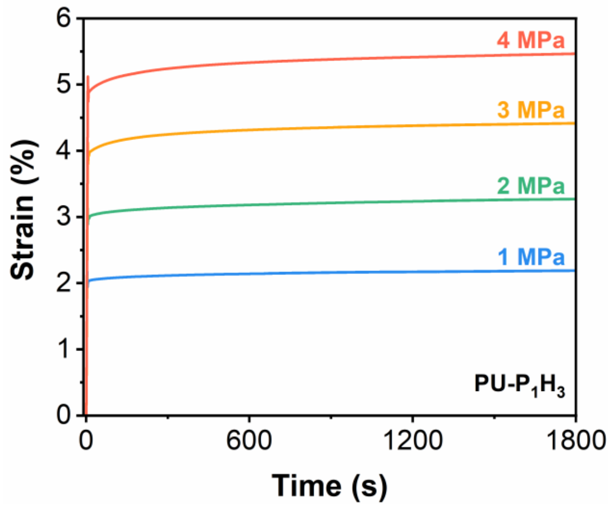

Supplementary Fig. 16 | Creep of the PU-P_1H_3 adhesive-bonded stainless steel joints at constant lap shear stress levels corresponding to 1, 2, 3, and 4 MPa.

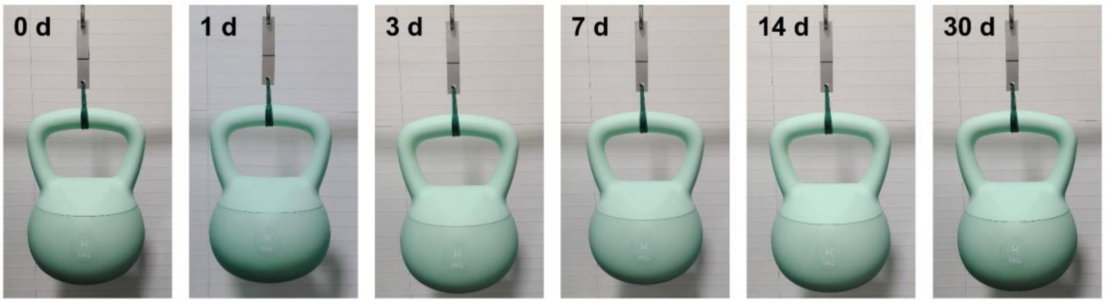

Supplementary Fig. 15 | Images of the PU-P_1H_3 adhesive-bonded stainless steel joints over a 30-day period under a sustained 8 kg load, showing no detectable creep or adhesive failure.

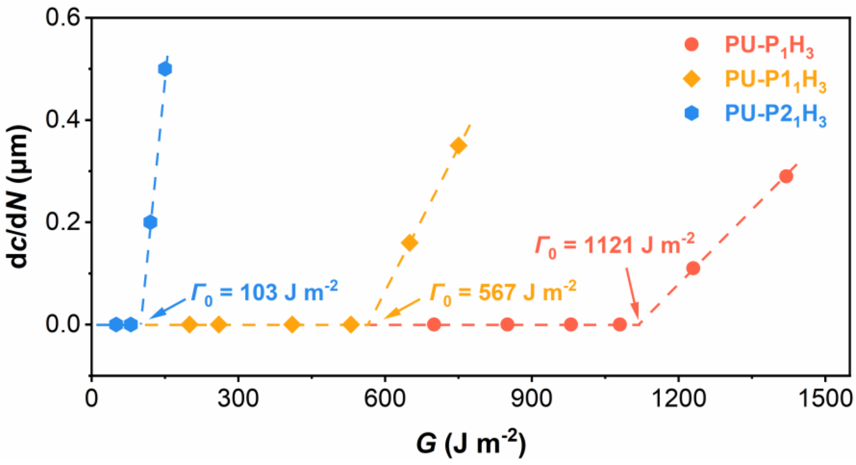

Fig. 5h | Interfacial fatigue threshold for PU-P_1H_3, PU-$P1_1H_3$, and PU-$P2_1H_3$ on stainless steel substrates.

(7) To bridge the gap between laboratory performance and real-world utility, the authors are encouraged to demonstrate the adhesive's use in a prototype device or a simulated real-world scenario.

Response: Thank you for your constructive comments regarding the application simulation of real-world scenario. Here, we supplemented the impact model for the endplate-cell interface connection in new energy battery modules (Fig. 6i).

In new energy battery, metal endplates and frames provide macroscopic constraints. The adhesive, meanwhile, serves dual roles: compensating for microscopic assembly gaps to prevent fretting wear, and more critically, acting as a continuous medium for uniform force distribution. Consequently, its adhesive performance, especially work of debonding during extreme dynamic loads such as collisions, directly governs the safety threshold of battery packs under mechanical abuse conditions. This model evaluates the independent load-bearing capacity of PU-P_xH_y adhesive as a critical (potentially final) energy absorption and failure-delay barrier when battery structures face extreme impacts.

As shown in Supplementary Fig. 26, T-joint specimens composed of stainless steel plates and rods bonded with PU-P_1H_3 and commercial adhesives (3M-DP6310NS, Loctite-480) were subjected to pendulum impact tests to evaluate their impact energy, simulating extreme dynamic loading scenarios in battery module endplate-cell interfaces. The results show that PU-P_1H_3, 3M-DP6310NS, and Loctite-480 adhesives exhibited impact energies of 7.98, 5.64, and 4.05 $kJ \cdot m^{-2}$, respectively (Fig. 6j). These results highlight the exceptional adhesive strength and debonding work of PU-P_1H_3, demonstrating its potential for applications demanding high energy absorption, impact resistance, or tolerance to sudden overloads.

All the revisions are added in the revised manuscript (Page 26-27, line 584-602) and marked in blue for reading.

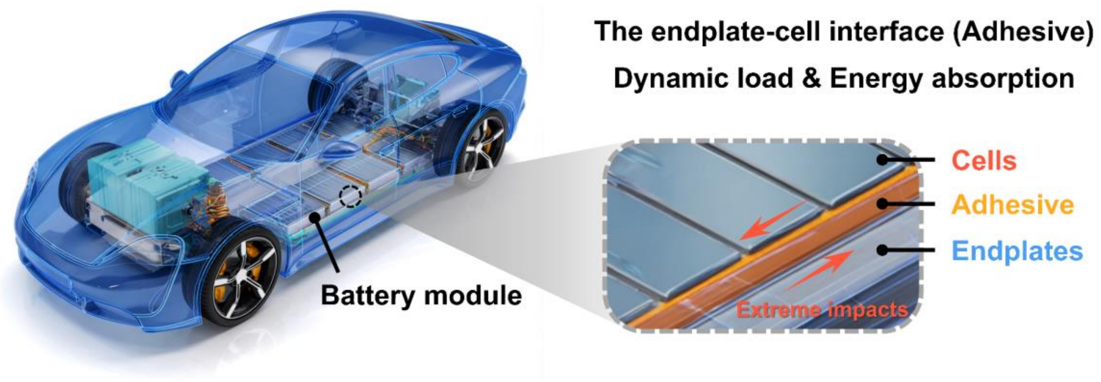

Fig. 6i | Schematic showing the electric vehicle battery module and its endplate-cell interfaces.

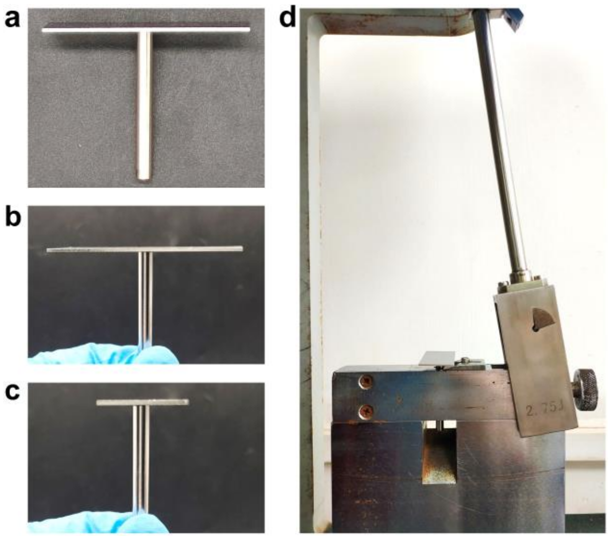

Supplementary Fig. 26 | Optical images showing PU-P_1H_3 adhesive-bonded stainless steel T-joint specimen during impact testing.

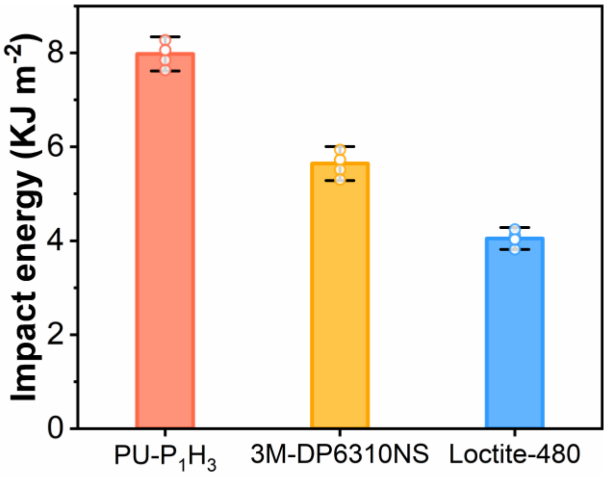

Fig. 6j | Comparison of impact energy for T-type stainless steel models bonded with PU-P_1H_3 adhesive and two different commercial adhesives.

Reviewer #2: The authors report a polyurethane adhesive that exhibits high adhesive strength and ultra-high work of debonding. The work integrates a dual-length dynamic crosslinked network with hierarchical organizations. The manuscript presents an amount of characterization data, and the reported numerical values for bulk mechanical properties are impressive. However, after a careful review, I have concerns regarding the fundamental design philosophy and the practical interpretation of the data. The manuscript appears to be a collection of modular characterizations where the connection between the high numerical values and actual adhesive utility is not fully justified. The study emphasizes bulk mechanical properties, while the critical analysis of interfacial failure mechanisms seems secondary. It appears that the high adhesive performance is merely a derivative of the high bulk tensile strength, rather than a result of specific interfacial engineering. To improve the scientific rigor and practical relevance of this work, significant revisions are required. The authors should address the following points:

General Response: We sincerely appreciate the reviewer for the thorough review and insightful comments. In response to your comments and suggestions, we have thoroughly revised the manuscript with a particular focus on strengthening the experimental validation and explanation of the adhesive-substrate interfacial interactions. A central revision is the decoupling of the contributions from bulk cohesion and interfacial adhesion, which allows us to more directly correlate the adhesive performance with interface engineering, rather than presenting it merely as a derivative of bulk mechanical properties. Overall, these revisions have refined the relationship between adhesion performance, bulk cohesion, and interfacial interactions, leading to a more robust and reasonable interpretation throughout the manuscript. A detailed point-to-point response to your valuable comments can be found below and all the change in the revised manuscript is marked in blue.

(1) The manuscript claims to simultaneously optimize bulk cohesion and interfacial adhesion. However, the results suggest that the performance is dominated by the bulk mechanics of the material. It appears the failure mode is primarily cohesive failure rather than adhesive failure, yet the interfacial adhesion mechanism is rarely discussed.

Response: We are grateful to the reviewer's professional comments for improving our manuscript. And we have provided point-by-point responses to the issues raised in the

comments as follows:

1) For the failure mode, we supplemented interfacial failure image of PU-P_1H_3 adhesive-bonded stainless steel joints. Notably, the interfacial failure in our work fundamentally differs from classical cases because interfacial interaction is comparable to bulk cohesion. Through deliberate design, this system simultaneously optimizes both bulk cohesion and interfacial adhesion to enhance adhesive performance. Remarkably, strain-induced crystallization during lap-shear dynamically strengthened the bulk material, strategically redirecting stress distribution to preferentially induce interfacial failure without adhesive residue (Supplementary Fig. 25). In contrast, when replacing the crystallizable PCL in PU-P_1H_3 with non-crystallizable polypropylene glycol (PPG), the resulting PU-$P1_1H_3$ adhesive (see comment 2 for details) exhibited a near-mixed failure mode.

2) For the interfacial adhesion mechanism, we supplement it as follows: As illustrated in Fig. 5a, during the hot pressing adhesion process, the MMSIs units with low relaxation time (see comment 4 for details) and PCL chain segments synergistically enhance interfacial adhesion through multiple non-covalent interactions (e.g., hydrogen bonding, cation-π interaction, metal-ion multiple oxygen binding). These interactions promote intimate contact between the adhesive and substrate, while partially achieving mechanical interlocks with microscopic interfacial gaps. Consequently, multiscale high energy phases can be in situ formed at the adhesive-substrate interface, comprising MMSIs hard domains and PCL crystals. They anchor at the interface and effectively hold the interfacial crack tip, thereby elevating the interfacial adhesion and fatigue threshold.

Admittedly, while our work synergistically optimized bulk cohesion and interfacial adhesion, the characterization and discussion of interfacial interactions and its mechanism in the original manuscript remained limited. We would emphasize that adhesive performance stems not from bulk mechanics but from bulk-interface coupling. Systematic interfacial studies now decouple these contributions, enabling direct correlation between adhesive performance and interfacial engineering rather than treating it as a bulk mechanics derivative. Considering this section largely overlaps with comment 2, we have provided a detailed discussion there to avoid redundancy. Here, we only briefly summarize the key points.

All the revisions are added in the revised manuscript (Page 20-24, line 462-542) and marked

in blue for reading.

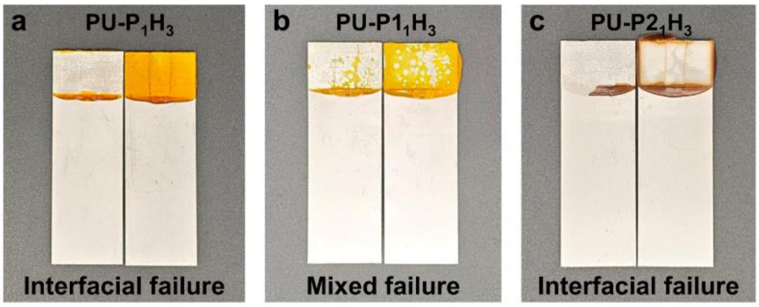

Supplementary Fig. 25 | Failure modes. a Interfacial failure (PU-P_1H_3), **b** mixed failure (PU-$P1_1H_3$), and **c** interfacial failure (PU-$P2_1H_3$).

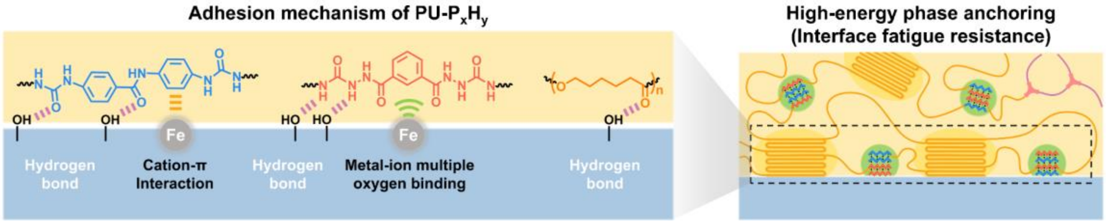

Fig. 5a | Interfacial interaction and fatigue-resistant interface adhesion mechanism.

(2) The substrate scope is limited, and there is little evidence showing how the multiscale structure specifically improves wetting or interfacial interaction distinct from bulk reinforcement. The authors should explain if the adhesive strength is simply controlled by the bulk modulus or strength.

Response: We sincerely appreciate the reviewer's constructive comments regarding the broad applicability of PU-P_xH_y adhesives and the contribution of interfacial interactions. In response to these two suggestions, we have supplemented the schematic diagram, relevant experiments, and detailed analyses in the revised manuscript.

1) For the substrate scope, PU-P_1H_3 adhesive exhibits superior bonding to polar substrates in the original manuscript, including metals (steel, Cu, Fe, Al), wood, glass, and polycarbonate (PC). Here, we supplemented additional lap shear strength of PU-P_1H_3 adhesive bonded on non-polar surfaces such as polyethylene (PE), polypropylene (PP), and polytetrafluoroethylene (PTFE) (Fig. 3f). Encouragingly, the results indicate that PU-P_1H_3 adhesive also exhibits promising adhesive strength on these low surface energy materials, with 4.96 MPa on PP, 4.44 MPa on PE, and 2.25 MPa on PTFE. This can be primarily ascribed to the intimate contact

between the adhesive and the substrate, which enables the formation of densely hydrogen-bonded MMSIs hard domains and PCL crystals, with both anchored at the interface. This not only significantly enhances interfacial adhesion but also contributes to a certain degree of mechanical interlocks.

2) For the interface adhesion and its mechanisms, we have conducted a systematic investigation. As illustrated in Fig. 5a, during the hot pressing adhesion process, the MMSIs units with low relaxation time (see comment 4 for details) and PCL chain segments synergistically enhance interfacial adhesion through multiple non-covalent interactions (e.g., hydrogen bonding, cation-π interaction, metal-ion multiple oxygen binding). These interactions promote intimate contact between the adhesive and substrate, while partially achieving mechanical interlocks with microscopic interfacial gaps. Consequently, multiscale high energy phases can be in situ formed at the adhesive-substrate interface, comprising MMSIs hard domains and PCL crystals. They anchor at the interface and effectively hold the interfacial crack tip, thereby elevating the interfacial adhesion and fatigue threshold. We first employed density functional theory (DFT) simulations (Fig. 5b, c and Supplementary Fig. 23) to elucidate the interfacial adhesion mechanism, where mononuclear Fe(III) hydroxo-aqua complexes were utilized to characterize the hydroxyl-terminated iron substrate surface. Both interaction region indicator (IRI) scatter plots and isosurface maps clearly confirm that IPDH and DABA engage in the aforementioned non-covalent interactions with the substrate. To elucidate the respective contributions of MMSIs hard domains and PCL crystals to interfacial adhesion, two additional prepolymers were synthesized for decoupled comparison (Fig. 5d). The soft segment in prepolymer 1 (P1) was substituted with polypropylene glycol (PPG), whereas prepolymer 2 (P2) was synthesized without MMSIs units, in contrast to the original prepolymer (P). Thus, the cured PU-$P1_1H_3$ adhesive contained only MMSIs hard domains, while PU-$P2_1H_3$ retained exclusively PCL crystals. Peak Force Quantitative Nanomechanical Mapping (PF QNM) was then employed to characterize the modulus and adhesion distributions across surface layers. As shown in Supplementary Fig. 24, PU-P_1H_3 exhibited higher and more broadly distributed surface modulus compared to PU-$P1_1H_3$ and PU-$P2_1H_3$, suggesting the accumulation of both high energy phases (MMSIs hard domains and PCL crystals) at its surface, achieved through multiscale design. Notably, the mean interfacial adhesion forces for PU-P_1H_3, PU-$P1_1H_3$, and

PU-P2$_1$H$_3$ were quantified as 0.53, 0.80, and 0.22 μN, respectively (Fig. 5e), demonstrating that MMSIs hard domains play a dominant role in interfacial adhesion enhancement, while PCL crystals exhibit a weaker but non-negligible contribution. We further conducted lap shear tests (Fig. 5f). The PU-P$_1$H$_3$ adhesive, which combines high bulk cohesion and strong interfacial interactions, exhibited the highest adhesive strength and work of debonding. Due to the absence of PCL crystals, the PU-P1$_1$H$_3$ adhesive showed reduced bulk cohesion, leading to decreased adhesive strength (9.96 MPa) and work of debonding (8137 N·m^{-1}). As for the PU-P2$_1$H$_3$ adhesive, the lack of MMSIs units substantially weakened interfacial interactions, resulting in significantly compromised adhesive properties. Besides, the strain-induced crystallization behavior of PU-P$_1$H$_3$ during lap shear test resulted in dynamic bulk reinforcement, enabling strategic stress redistribution that preferentially initiated atypical interfacial failure without residual adhesive (Supplementary Fig. 25). In contrast, PU-P1$_1$H$_3$ containing non-crystallizable PPG exhibited a predominantly mixed failure mode, while PU-P2$_1$H$_3$ with weaker interfacial interactions showed characteristic interfacial failure behavior. These differences highlight the distinct roles of hierarchical structure: PCL crystals primarily enhance bulk cohesion, while MMSIs hard domains are critical for improving interfacial interactions. The above analysis confirms that PU-P$_x$H$_y$ adhesives form strong interactions with functional groups (e.g., hydroxyl and metal ions) on the substrate through surface-enriched non-covalent units such as ester, urea, amide, and phenyl groups. Then, the resulting multiscale high energy phases (MMSIs hard domains and PCL crystals) become densely anchored at the adhesive-substrate interface, leading to significantly enhanced interfacial adhesion of the adhesive coatings.

To further elucidate how the multiscale structures synergistically enhance both bulk cohesion and interfacial adhesion, we performed 90° peel tests on stainless steel substrates to decouple and quantify both interfacial fracture toughness and fatigue threshold. As shown in Fig. 5g, PU-P$_1$H$_3$, PU-P1$_1$H$_3$, and PU-P2$_1$H$_3$ exhibited interfacial fracture toughness values of 4125, 913 and 2957 J m^{-2}, respectively, further evidencing the dominant role of interfacial MMSIs hard domains in adhesion enhancement. Besides, PU-P$_1$H$_3$ yielded superior interfacial fatigue threshold (Γ_0 = 1121 J m^{-2}) compared to both counterparts (Fig. 5h), demonstrating that co-anchored MMSIs hard domains and PCL crystals effectively hold the interfacial crack tip while dissipating energy. These quantitative metrics provide compelling support for the synergistic

improvement of both bulk cohesion and interfacial adhesion through multiscale engineering.

All the revisions are added in the revised manuscript (Page 16, line 358-360, and page 20-24, line 462-542) and marked in blue for reading.

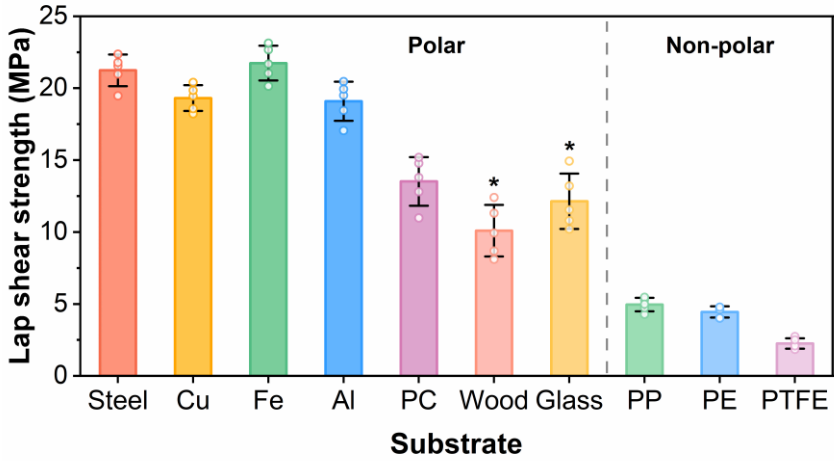

Fig. 3f | Lap shear strength of PU-P_1H_3 adhesive bonded on different substrates.

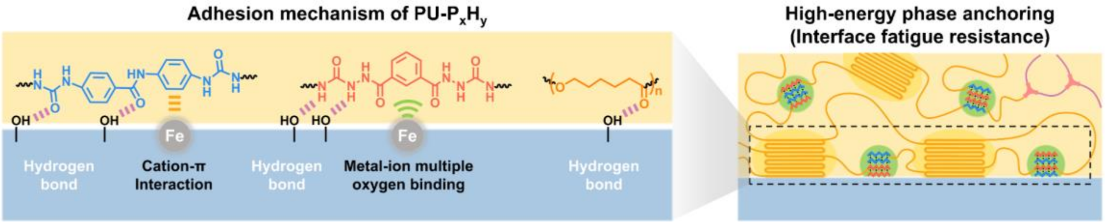

Fig. 5a | Interfacial interaction and fatigue-resistant interface adhesion mechanism.

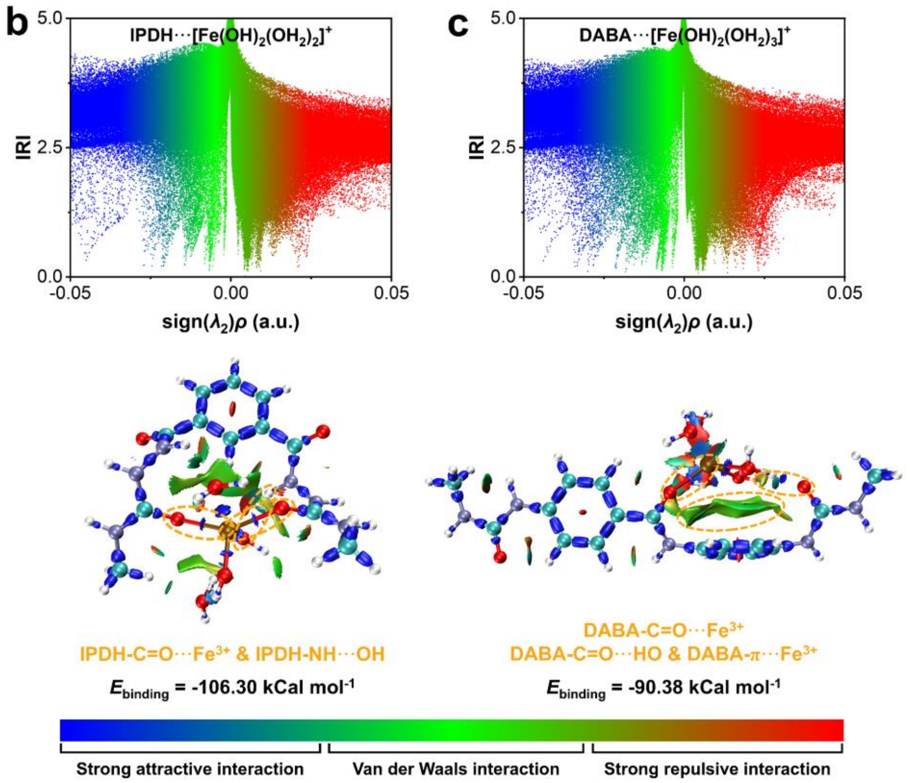

Fig. 5b-c | Plots of IRI versus sign(λ_2)ρ and the gradient isosurfaces for the complexes: **b** IPDH···$[Fe(OH)_2(OH_2)_2]^+$, **c** DABA···$[Fe(OH)_2(OH_2)_3]^+$.

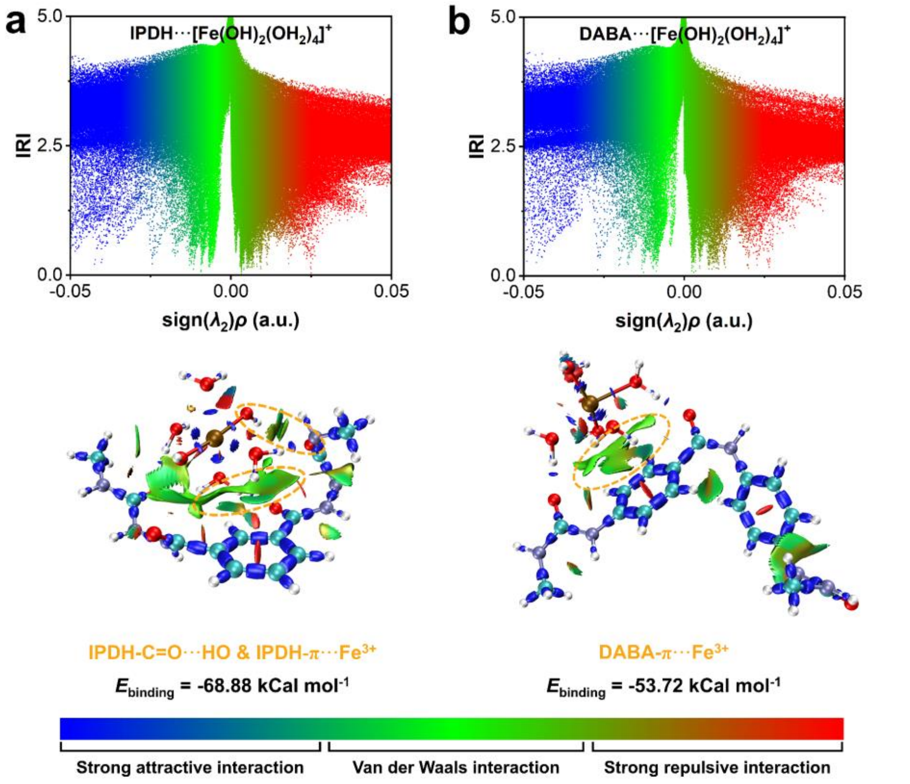

Supplementary Fig. 23 | Plots of IRI versus sign(λ_2)ρ and the gradient isosurfaces for the complexes: **a** IPDH···$[Fe(OH)_2(OH_2)_4]^+$, **b** DABA···$[Fe(OH)_2(OH_2)_4]^+$.

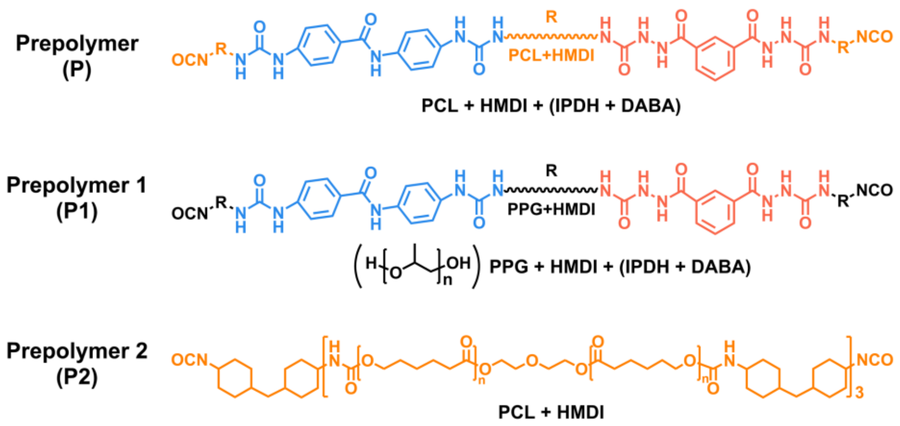

Fig. 5d | Structures of the three prepolymers, imparting distinct multi-scale organizations.

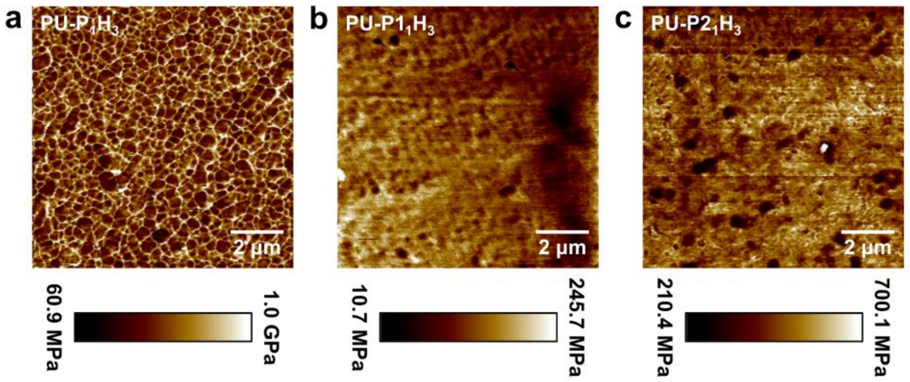

Supplementary Fig. 24 | AFM-based surface modulus measurements. a PU-P_1H_3, **b** PU-$P1_1H_3$, and **c** PU-$P2_1H_3$.

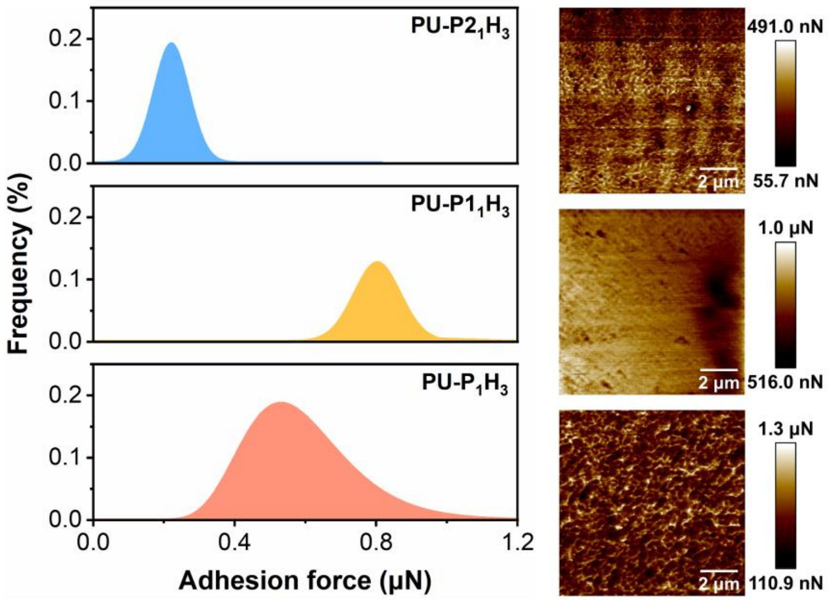

Fig. 5e | Adhesion force distribution maps for PU-P_1H_3, PU-$P1_1H_3$, and PU-$P2_1H_3$ adhesives.

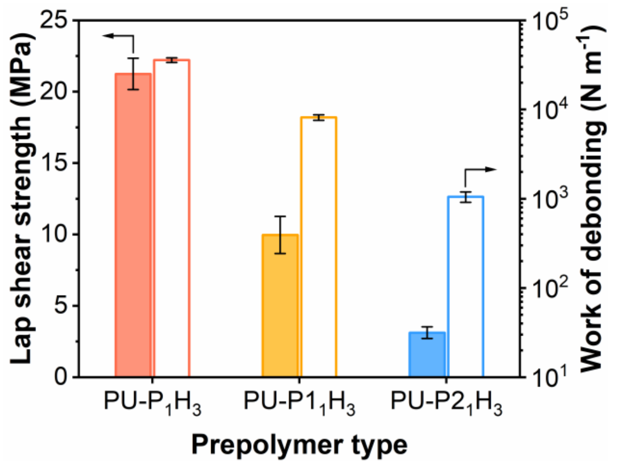

Fig. 5f | Comparison of the adhesion properties of PU-P$_1$H$_3$, PU-P1$_1$H$_3$, and PU-P2$_1$H$_3$ adhesives.

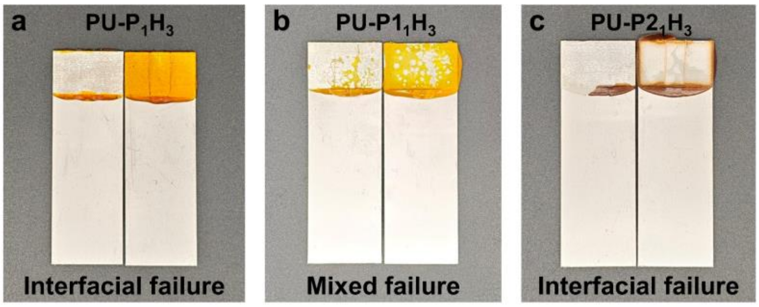

Supplementary Fig. 25 | **Failure modes. a** Interfacial failure (PU-P$_1$H$_3$), **b** mixed failure (PU-P1$_1$H$_3$), and **c** interfacial failure (PU-P2$_1$H$_3$).

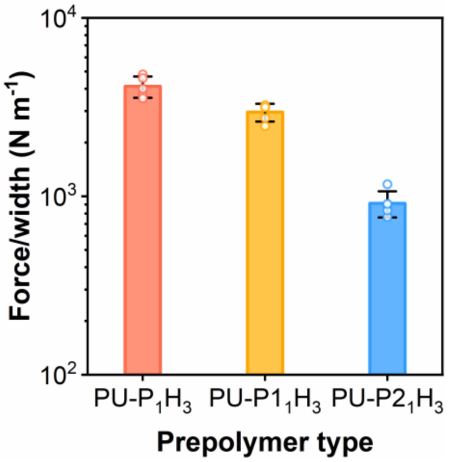

Fig. 5g | Interfacial fracture toughness for PU-P$_1$H$_3$, PU-P1$_1$H$_3$, and PU-P2$_1$H$_3$ on stainless steel substrates.

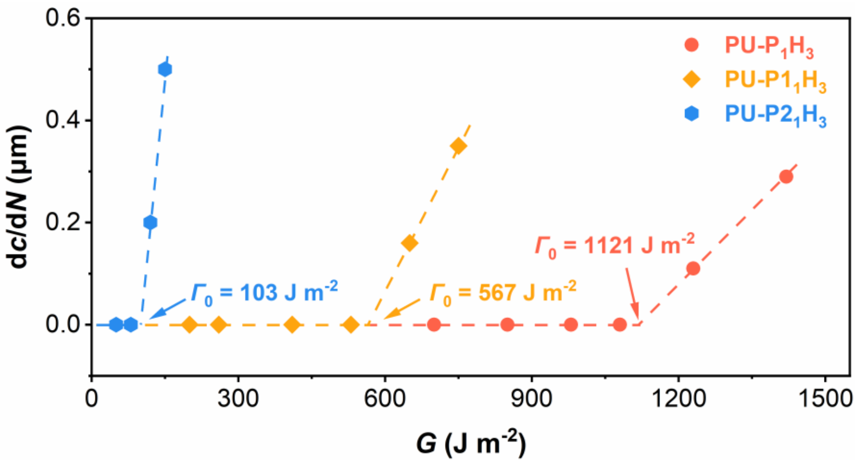

Fig. 5h | Interfacial fatigue threshold for PU-P_1H_3, PU-$P1_1H_3$, and PU-$P2_1H_3$ on stainless steel substrates.

(3) The authors highlight the ultra-high work of debonding (35,731 N m^{-1}) as a major advantage, but this value is derived from massive elongation (displacement > 3.5 mm). Typical structural adhesives fracture at much lower displacements (e.g., ~0.8 mm) to maintain structural rigidity. A joint that undergoes millimeter-scale deformation under load may compromise the assembly's integrity or suffer from creep at low stress levels. Is this high ductility practically useful for an adhesive, or is it simply a high numerical value derived from the material's elastomeric nature? Are there any specific applications that require such extreme compliance?

Response: Thank you for raising this important point regarding the large shear displacement (> 3.5 mm) observed in PU-P_1H_3 adhesive. We acknowledge that this value exceeds the typical range (~0.8 mm) of typical structural adhesives. And we also agree that such high compliance could raise concerns about integrity under sustained load or potential creep at low stress levels. To address this directly, we have conducted a series of creep experiments under lap shear configuration at constant lap shear stress levels corresponding to 1, 2, 3, and 4 MPa, which represent anticipated low service loads (Supplementary Fig. 16). The results indicate that under these conditions, the creep strain all stabilized within 30 minutes, suggesting that the adhesive can maintain its creep within an acceptable range over extended periods under typical service loads, thereby ensuring dimensional stability. In addition, as a practical demonstration, PU-P_1H_3 adhesive-bonded stainless steel joints successfully supported a weight of 8 kg for 30 days without detectable creep or adhesive failure (Supplementary Fig. 15).

The large displacement (> 3.5 mm) reported corresponds to the ultimate failure strain under high load, not to deformation under service conditions. This extreme ductility can be advantageous in applications where energy absorption, impact resistance, or tolerance to sudden overloads is critical, such as dynamic loading environments, vibration damping, or structures requiring crack bridging or stress redistribution. Thus, the high ductility is not merely a result of high numerical value derived from the material's elastomeric nature, but a deliberate bulk material property that offers functional benefits in specific scenarios, combining high toughness with adequate shear strength.

For illustrative purposes, we also supplemented the impact model for the endplate-cell interface connection in new energy battery modules (Fig. 6i). In new energy battery, metal endplates and frames provide macroscopic constraints. The adhesive, meanwhile, serves dual roles: compensating for microscopic assembly gaps to prevent fretting wear, and more critically, acting as a continuous medium for uniform force distribution. Consequently, its adhesive performance, especially work of debonding during extreme dynamic loads such as collisions, directly governs the safety threshold of battery packs under mechanical abuse conditions. This model evaluates the independent load-bearing capacity of PU-P_xH_y adhesive as a critical (potentially final) energy absorption and failure-delay barrier when battery structures face extreme impacts. As shown in Supplementary Fig. 26, T-joint specimens composed of stainless steel plates and rods bonded with PU-P_1H_3 and commercial adhesives (3M-DP6310NS, Loctite-480) were subjected to pendulum impact tests to evaluate their impact energy, simulating extreme dynamic loading scenarios in battery module endplate-cell interfaces. The results show that PU-P_1H_3, 3M-DP6310NS, and Loctite-480 adhesives exhibited impact energies of 7.98, 5.64, and 4.05 $kJ \cdot m^{-2}$, respectively (Fig. 6j). These results highlight the exceptional adhesive strength and debonding work of PU-P_1H_3, demonstrating its potential for applications demanding high energy absorption, impact resistance, or tolerance to sudden overloads.

All the revisions are added in the revised manuscript (Page 14, line 314-316, 318-321, and page 26-27, line 584-602) and marked in blue for reading.

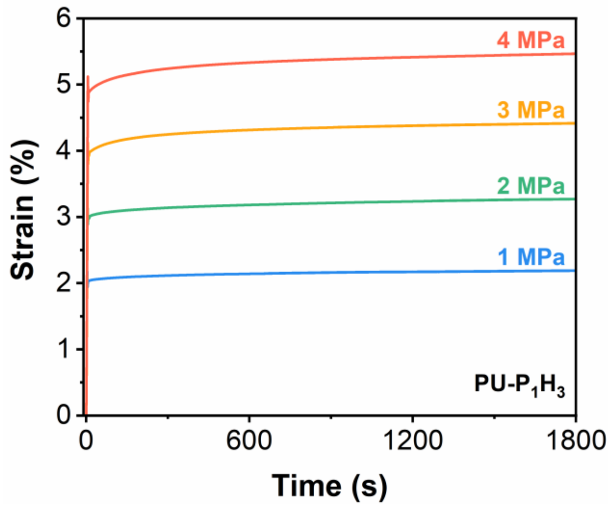

Supplementary Fig. 16 | Creep of the PU-P_1H_3 adhesive-bonded stainless steel joints at constant lap shear stress levels corresponding to 1, 2, 3, and 4 MPa.

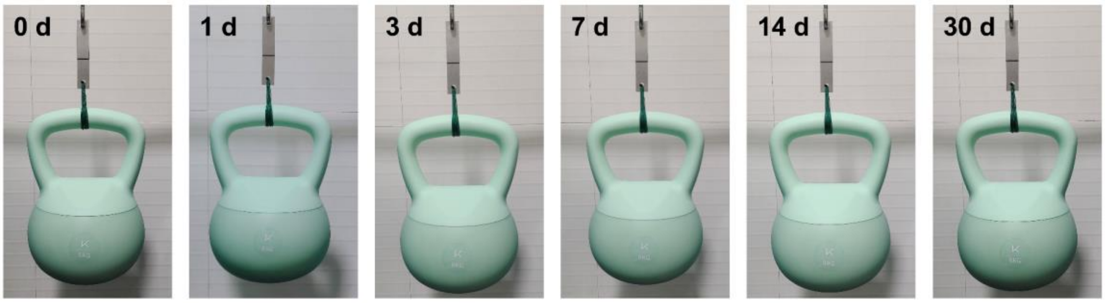

Supplementary Fig. 15 | Images of the PU-P_1H_3 adhesive-bonded stainless steel joints over a 30-day period under a sustained 8 kg load, showing no detectable creep or adhesive failure.

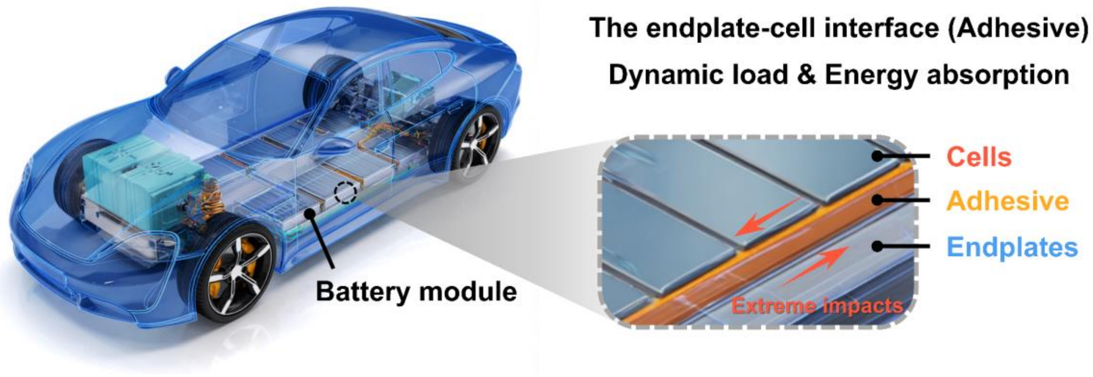

Fig. 6i | Schematic showing the electric vehicle battery module and its endplate-cell interfaces.

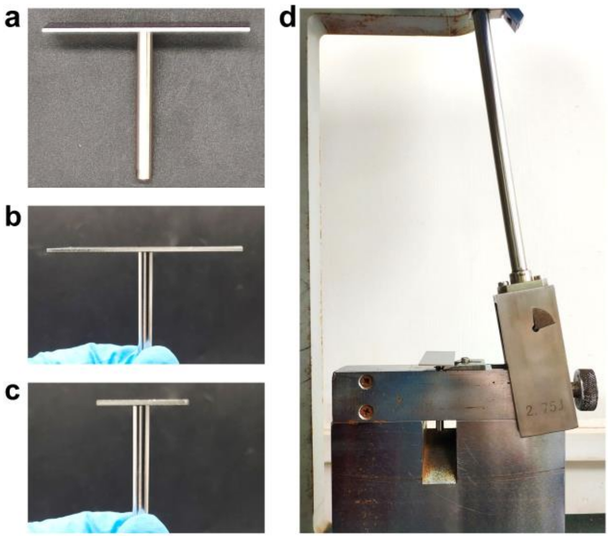

Supplementary Fig. 26 | Optical images showing PU-P_1H_3 adhesive-bonded stainless steel T-joint specimen during impact testing.

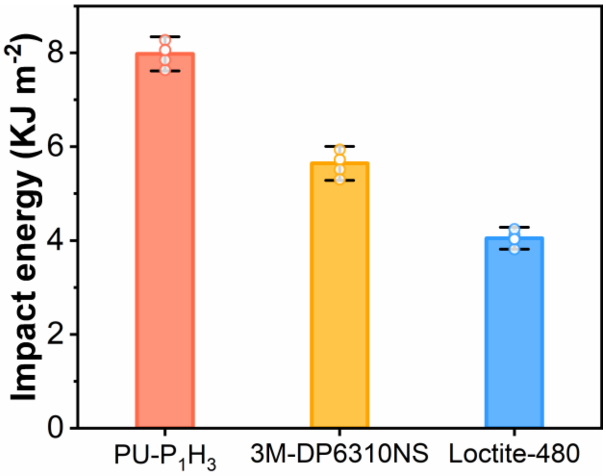

Fig. 6j | Comparison of impact energy for T-type stainless steel models bonded with PU-P_1H_3 adhesive and two different commercial adhesives.

(4) The authors rely on BDS data at -90 °C to explain beta/gamma relaxations. However, adhesive performance is evaluated at room temperature. According to time-temperature superposition, relaxations at -90 °C correspond to extremely high frequencies at room temperature. The authors should justify how these specific molecular motions at cryogenic temperatures contribute to quasi-static adhesive strength at service temperatures.

Response: Thank you for your valuable comment. In response to the controversy surrounding the mismatch between the low-temperature testing environment of BDS for PU-P_xH_y adhesives and its room temperature usage scenarios, we would like to provide the following explanation:

Firstly, the purpose of BDS tests is to utilize it as a high-resolution spectroscopy to identify and compare the local mobility of two different length crosslinked chains, rather than directly simulating the conditions of room temperature quasi-static testing. Regarding the test temperature, only at cryogenic temperatures do the β and γ relaxation peaks exhibit high resolution and can be effectively separated, allowing for accurate quantification of their respective relaxation characteristics and activation energies (E_a). Contrarily, at room temperature, due to the dominance of α relaxation and peak overlap, it is difficult to distinguish secondary relaxation.

Secondly, the fitted intrinsic thermodynamic parameter E_a reflects the essential differences in temperature sensitivity among different secondary relaxations. The β relaxation associated with the local motion of crosslinked long chains exhibits a lower E_a compared to that of γ relaxation which related to the local motion of crosslinked short chains. This indicates that at the molecular level, the local motion energy barriers of long chains containing MMSIs units are lower. Although its exact relaxation time at room temperature is influenced by free volume and coupling effects, this inherent and easily excitable motion characteristic ensures that, compared to motion modes requiring higher E_a, the adhesive functional groups on crosslinked long chains have relatively higher conformational adjustment capabilities and interfacial diffusion probabilities across the entire temperature-time scale of adhesive application and interfacial bonding. This provides a kinetic advantage for PU-P_xH_y adhesives to achieve superior interfacial adhesion. Therefore, BDS tests at low temperatures reveals, to some extent, molecular motion and segment behavior during the bonding process at room temperature.

Finally, regarding the issue of "relaxations at -90 °C correspond to extremely high frequencies at room temperature (i.e. extremely short relaxation time)". This rapid molecular dynamics brings dual advantages to adhesive performance. Specifically, during the adhesive application process, it promotes the adhesive to quickly wet the surface of the substrate and complete the initial anchoring. More importantly, it provides a dynamic driving force for functional groups to efficiently search for and form a large number of strong interactions with active sites on the substrate surface (such as hydrogen bonding, cation-π interaction, metal-ion multiple oxygen binding), thereby maximizing the interfacial adhesion strength. This is also an important proof of the interfacial interaction in this work.

All the revisions are added in the revised manuscript (Page 15, line 332-339) and marked in blue for reading.

(5) What is the specific rationale for using HMDI for the prepolymer while using HDI for the short-chain crosslinking? Additionally, why was 140 °C chosen for hot pressing? How would the performance change if hot pressing were conducted at higher or lower temperatures?

Response: We sincerely thank the reviewer for this valuable comment regarding the raw material components and process parameters. And we have provided responses to each point as follows:

1) The reasons for using HMDI for the prepolymer while using HDI for the short-chain crosslinking is as follows: HMDI contains cyclohexane rings, which helps to improve rigidity and heat resistance. At the same time, the polar HMDI increases the thermodynamic incompatibility between the soft and hard segments, thus promotes microphase separation and enhances bulk cohesion. It is worth noting that the cyclohexane ring in HMDI has to some extent disrupted the regularity, resulting in the nanoscale hard domain not being highly regular and evolving into hard segment crystals. As we all know, compared with hard segment crystallization, ordered yet non-crystalline microphase hard domains enable dynamic responsiveness of internal hydrogen bond networks, achieving a balance between strength and toughness. On the other hand, regarding the selection of short crosslinked segments, we opted for HDI due to its lower steric hindrance and higher flexibility, based on considerations of segmental steric effects and their impact on the degree of reaction.

2) The reasons for choosing 140 °C as the hot pressing temperature is as follows: it represents the optimal viscoelastic balance for the PU-P_1H_3 adhesive. Rheological data (Supplementary Fig. 14) indicate that this temperature lies between the relaxation peak of the MMSIs hard domains physical crosslinked network (130.4 °C) and the solid-liquid transition point (160.3 °C). At 140 °C, PU-P_1H_3 exhibits sufficient flowability for effective interfacial wetting while retaining sufficient viscoelastic integrity to resist excessive flow, achieving an optimal balance between processability and shape retention. As the temperature decreases, the MMSIs hard domains network remains insufficiently softened, resulting in poor flowability, inadequate wetting, and low adhesive strength. Conversely, As the temperature increases, the network

disintegrates, causing the adhesives to behave as a nearly pure viscous fluid. This leads to issues such as adhesive overflow and increased risk of thermal degradation. Besides, increasing the temperature also imposes limitations on the bonded substrates. Therefore, after comprehensive consideration in the early stage, we have set the hot pressing temperature to 140 °C.

All the revisions are added in the revised manuscript (Page 5, line 105-106, 110-111, and page 13, line 299-303) and marked in blue for reading.

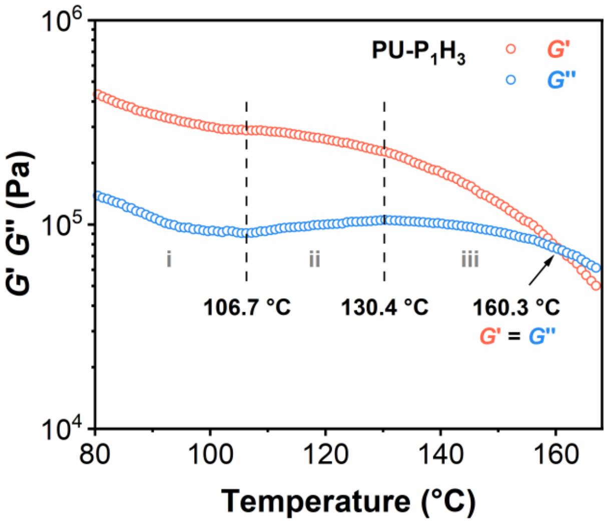

Supplementary Fig. 14 | Temperature-sweep rheological curves of PU-P_1H_3 from 80 to 168 °C.

(6) Conceptual and terminological accuracy:

-"Top-Down" Strategy: The authors describe their synthesis as "top-down." In materials science, synthesizing polymers from monomers is a classic "bottom-up" approach. This terminology is misleading.

-"Cross-linked Long Chain": The authors refer to the prepolymer as a "cross-linked long chain." However, the prepolymer appears to be linear at that stage.

Response: We thank the reviewer for pointing out this inaccurate terminology. We are sorry for the unnecessary negligence and have modified "Cross-linked Long Chain" to "Long Chain used for Crosslinking".

Regarding the description of "Top-down strategy", we would like to provide the following explanation: "Top-down" refers to the philosophy of structural design (see original manuscript, Page 1, line 12-15, and page 3-4, line 75-85), rather than the "bottom-up" synthesis method. Integrating multi-scale structures in cross-linked polymers is difficult and rarely reported. For

example, for conventional crosslinked polyurethane, the constrained short chains between crosslinks severely inhibit those efficient strengthening and toughening strategies employed in thermoplastic polyurethane systems, such as crystallization, microphase separation, and hierarchical hydrogen bonds. By redesigning the fundamental thermoset framework from a macro perspective and then constructing an ingenious dual-length crosslinked network, we have combined thermoplastic and thermosetting characteristics to overcome this challenge, thus paving the way for the effective application of these strengthening and toughening strategies in previously incompatible thermoset polyurethane systems. Subsequently, based on the actual performance requirements of adhesives, we employed top-down design of molecular building blocks to achieve the integration of multi-scale organizations. Therefore, we describe it as a "top-down multiscale structure".

What we want to express about "Cross-linked Long Chain" is that the prepared prepolymer is subsequently used to form long cross-linked chains in a double length cross-linked network. To accurately express and avoid misunderstandings, we have adjusted it to "Long Chain used for Crosslinking"

All the revisions are added in the revised manuscript (Page 7, line 142, and page 30, line 659-660) and marked in blue for reading.

Reviewer #3: The authors design a polyurethane adhesive with multi-scale architecture that integrates dual-length dynamic crosslinks. This strategy successfully combines thermoplastic and thermosetting characteristics, achieving a balance between high strength, toughness, environmental stability, and recyclability. Overall, this is a nice and comprehensive work. I would like to recommend it to be published after addressing the following points.

General Response: We sincerely appreciate the reviewer for reviewing the manuscript and providing valuable and constructive comments as "*This strategy successfully combines thermoplastic and thermosetting characteristics*". All the significant issues have been carefully considered and addressed in the revised manuscript. A detailed point-to-point response to your valuable comments can be found below.

(1) How about the quantitative contribution of each hierarchical structure across multiple length scales to the adhesive strength and work of debonding?

Response: Thank you for raising this important point regarding the quantitative contribution of each hierarchical structure to the adhesive strength and work of debonding.

The contribution of dual-length crosslinked network on adhesive performance has been discussed in the original manuscript (Fig. 3b and Supplementary Table 9). And hierarchical H-bonds rely on PCL chains and MMSIs units to exist. Therefore, we mainly investigate the contribution of PCL crystals and MMSIs hard domains on adhesive strength and work of debonding. In an attempt to isolate each structure, we synthesized controlled systems designed to predominantly exhibit one of the two. However, as correctly noted, their strong interdependence makes complete decoupling synthetically challenging, which may also inadvertently alter other system parameters. Therefore, our comparative studies provide valuable semi-quantitative insights into their contribution. And we have discussed the observed trends with this limitation in mind. Further mechanistic quantification could be an interesting direction for future study.

We synthesized two additional prepolymers for decoupled comparison (Fig. 5d). Specifically, the soft segment in prepolymer 1 (P1) was substituted with polypropylene glycol (PPG), whereas prepolymer 2 (P2) was synthesized without MMSIs units (IPDH+DABA), in contrast to the original prepolymer (P). Thus, the cured PU-$P1_1H_3$ adhesive contained only MMSIs hard

phase domains, while PU-P2$_1$H$_3$ retained exclusively PCL crystals. As shown in Fig. 5f, the PU-P$_1$H$_3$ adhesive, which combines high bulk cohesion and strong interfacial interaction, exhibited the highest adhesive strength and work of debonding. Due to the absence of PCL crystals, the PU-P1$_1$H$_3$ adhesive showed reduced bulk cohesion, leading to decreased adhesive strength (9.96 MPa) and work of debonding (8137 N·m^{-1}). As for the PU-P2$_1$H$_3$ adhesive, the lack of MMSIs units substantially weakened interfacial interactions, resulting in significantly compromised adhesive properties. Our control experiment successfully depicted the different contribution of hierarchical structure: PCL crystals primarily enhance bulk cohesion, while MMSIs hard domains are critical for improving interfacial interactions.

All the revisions are added in the revised manuscript (Page 22, line 491-497, 505-511) and marked in blue for reading.

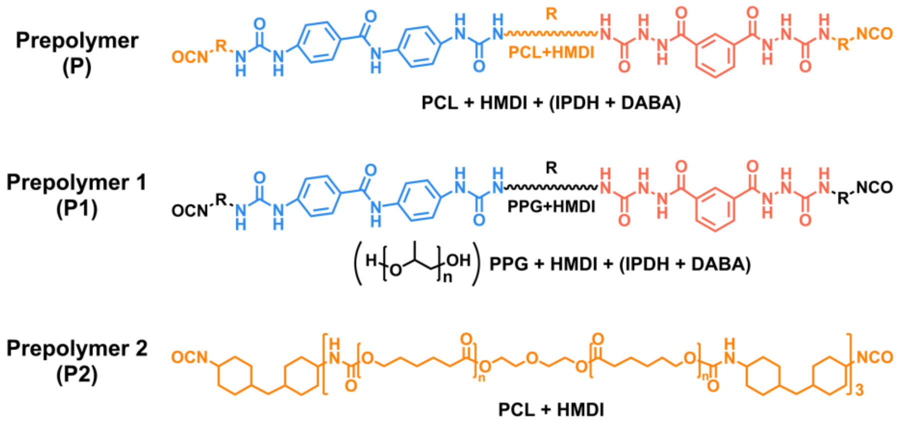

Fig. 5d | Structures of the three prepolymers, imparting distinct multi-scale organizations.

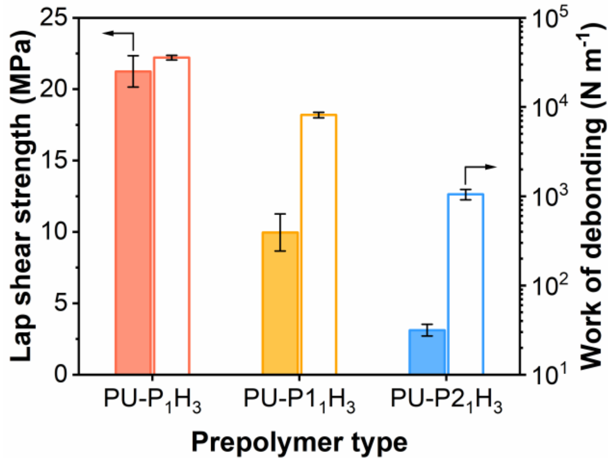

Fig. 5f | Comparison of the adhesion properties of adhesives cured with different prepolymers.

(2) The authors present the temperature-dependent dynamics of hydrogen bonding. Will it be feasible to conduct similar investigations into the thermal evolution of other hierarchical structures such as mesoscale PCL crystallite and nanoscale MMSI hard domain. How about the temperatures where each hierarchical structure begins to disintegrate?

Response: Thank you for this constructive comment, which helps us to gain a deeper understanding of the temperature-dependent dynamics of other hierarchical structures. Here, we supplemented variable-temperature polarized optical microscopy (POM) images (Supplementary Fig. 12) and temperature-sweep rheological curves (Supplementary Fig. 14) of PU-P_1H_3, reflecting the temperature-dependent structural evolution processes of mesoscale PCL crystallite and nanoscale MMSIs hard domain, respectively.

For PCL crystallite, the POM images visually demonstrate that they maintain structural stability up to 40 °C, begin to disintegrate at 45 °C, and essentially disappear at 55 °C. This result aligns with the crystallization melting peak observed in differential scanning calorimetry (Supplementary Fig. 9) and the sharp drop range in modulus seen in temperature sweep dynamic mechanical analysis curves (Fig. 6c).

For MMSIs hard domains, temperature-sweep rheological curves indicate that they begin to disintegrate at 106.7 °C, and essentially disappear at 160.3 °C (Supplementary Fig. 14). Specifically, the initial increase in the loss modulus (G'') at 106.7 °C marks the onset of microphase dissociation. At this temperature, thermal energy becomes sufficient to disrupt the hydrogen bonds that stabilize the MMSIs hard domains, initiating their softening and mobilization. The subsequent broad peak in G'', with a maximum at 130.4 °C, represents the most intensive stage of cooperative relaxation and viscous flow within the non-crystalline hard domains. This temperature corresponds to the effective softening of the MMSIs hard domains, where energy dissipation is maximal. However, a residual physical network persists at this stage, maintaining a solid-like response ($G' > G''$). Finally, the solid-liquid transition temperature (160.3 °C) signifies the complete functional failure of the MMSIs hard domains as an effective physical network.

All the revisions are added in the revised manuscript (Page 12, line 258-260, and page 13, line 299-303) and marked in blue for reading.

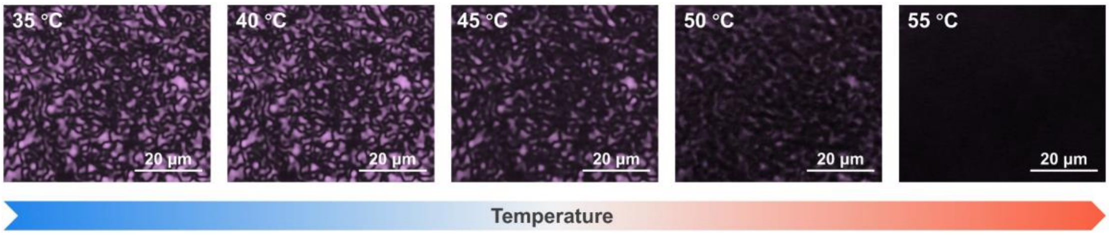

Supplementary Fig. 12 | Variable-temperature polarized optical microscopy images of PU-P_1H_3 from 35 to 55 °C, with 5 °C increments.

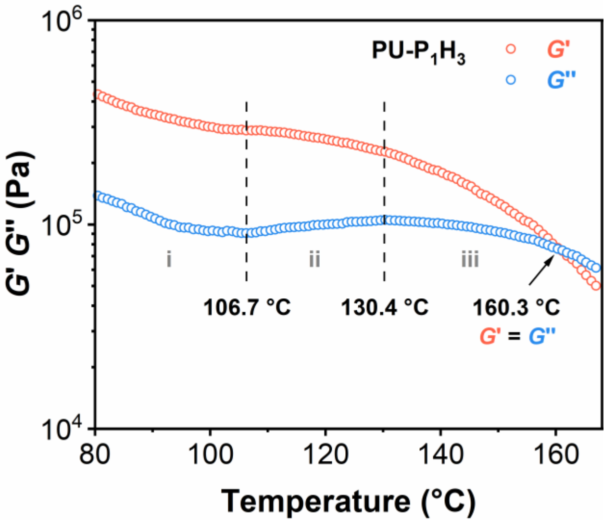

Supplementary Fig. 14 | Temperature-sweep rheological curves of PU-P_1H_3 from 80 to 168 °C.

(3) The dynamic phenol-carbamate bonds are key design of this adhesive. Relevant references are recommended to be cited. E.g. Science Bulletin 2024, 69(12), 1875-1886. Science China Materials 2022, 65(9), 2553–2564. Chemical Engineering Journal 2022, 430(4), 133081.

Response: We thank the reviewer for highlighting the importance of the dynamic phenol-carbamate bonds and for the constructive suggestion to include additional relevant references. As suggested, we have cited the recommended literature in the "Recyclability of PU-P_xH_y adhesives" section of revised manuscript (Page 25, line 552), and the changes have been marked in blue for reading.

(4) Figure 2h illustrates strain-induced crystallization. The stretching direction is recommended to provide.

Response: We sincerely thank the reviewer for this valuable suggestion regarding the clarity of Figure 2h. Upon careful review of this figure, we would like to clarify that Figure 2h is actually

polarized optical microscopy images of PU-P_1H_3 in its unstretched state, rather than depicting strain-induced crystallization. And the spherulites indicate that PU-P_1H_3 is in a semi-crystalline, isotropic state.

(5) Is the strain-induced crystallization (SIC) of PCL reversible after multiple loading cycles? Have fatigue or cyclic tensile tests been performed to confirm the durability of SIC?

Response: Thank you for raising this important question regarding the reversibility and durability of strain-induced crystallization (SIC) in the PCL segments. Here, we supplemented optical photos of PU-P_1H_3 over multiple tensile cycles (600% strain) to directly confirm the reversibility and durability of the SIC (Supplementary Fig. 13).

Indeed, PU-P_1H_3 yields during tensile deformation due to cross-linking and initial crystallization. SIC occurs at strains significantly beyond the yield point, accompanied by macroscopic stress whitening. To specifically examine the reversibility and durability of SIC, we performed multiple loading-unloading cycles up to 600% strain, and relied on direct optical observation rather than stress-strain curves. This approach is based on the following rationale: The stress-strain response is a complex integration of multiple factors, including the yielding of initial crystallites, hard segment dynamics, and irreversible network damage (e.g., Mullins effect), all of which contribute to significant hysteresis and evolving mechanical behavior. These overlapping factors make it difficult to isolate and attribute specific features in the cyclic curves solely to the reversible formation and melting of SIC. In contrast, the macroscopic stress whitening is a direct and unequivocal optical signature of SIC in our system.

We obtained direct visual evidence of SIC reversibility and durability over multiple tensile cycles (600% strain). In each cycle, the sample consistently produced macroscopic stress whitening upon stretching and regained transparency upon unloading. Representative optical photos after the 1st, 10th, and 100th cycles are provided below (Supplementary Fig. 13). These photos clearly show that the SIC phenomenon remains reversible over multiple tensile cycles, confirming its durability under cyclic loading. This reversibility has a solid physicochemical foundation. Thermodynamically, the crystals formed under strain are thin lamellar PCL crystallites. According to the Thompson-Gibbs equation, their effective melting point is significantly lower than that of well-developed equilibrium crystals, rendering them metastable

at room temperature. Kinetically, upon unloading, the stored entropic elasticity of the crosslinked network provides a powerful recovery driving force. This force is sufficient to overcome the cohesive energy of these non-equilibrium crystallites, prompting their rapid and complete melting upon stress removal. Therefore, the macroscopic optical reversibility we captured is a direct manifestation of this reversible structural transformation at the molecular level.

All the revisions are added in the revised manuscript (Page 12, line 265-266) and marked in blue for reading.

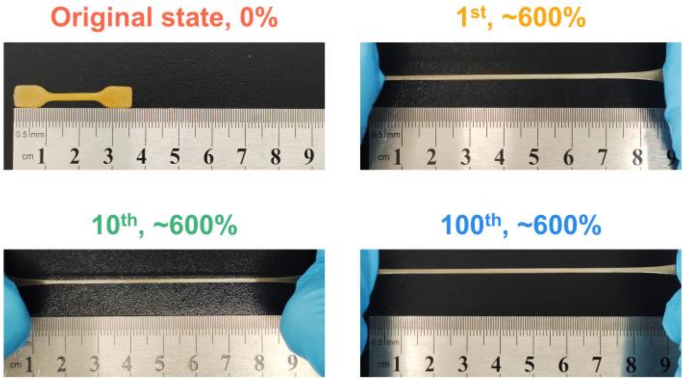

Supplementary Fig. 13 | Optical photos showing strain-induced crystallization of PU-P_1H_3 over multiple tensile cycles (600% strain).

(6) Could the authors supplement the data for the heat resistance with a rheological temperature sweep to pinpoint the solid-liquid transition temperature (G' = G'') of the materials?

Response: Thank you for your valuable suggestion on the solid-liquid transition temperature of the material. Here, we supplemented the temperature-sweep rheological curves of PU-P_1H_3 (Supplementary Fig. 14). Overall, the storage modulus (G') and loss modulus (G'') of PU-P_1H_3 show a decreasing trend with temperature. It is worth noting that due to the highly heat-resistant MMSIs microphase hard domains serving as strong physical crosslinking points, the decrease in G' and G'' at 90-140 °C is not significant. When the MMSIs microphase hard domains begin to disintegrate, G' begins to accelerate its descent and eventually intersects with G'' at 160.3 °C. All the revisions are added in the revised manuscript (Page 13, line 299-303) and marked in blue for reading.

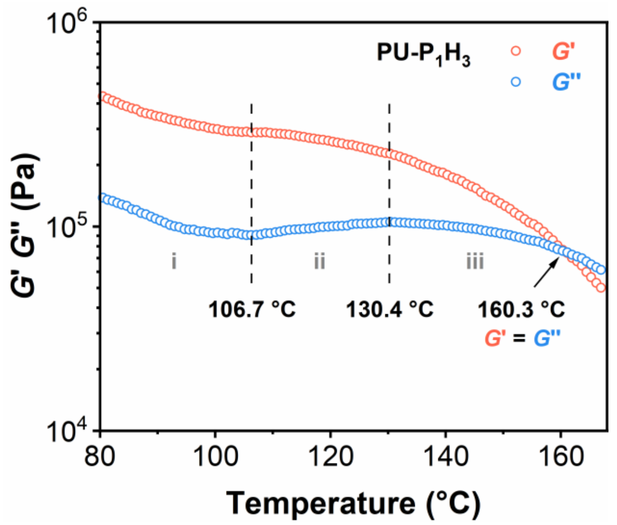

Supplementary Fig. 14 | Temperature-sweep rheological curves of PU-P_1H_3 from 80 to 168 °C.

(7) The adhesive exhibits superior bonding to polar substrates. Its performance on non-polar surfaces such as polypropylene (PP), polyethylene (PE), or polytetrafluoroethylene (PTFE) are recommended to be explored.

Response: We sincerely appreciate the reviewer's valuable suggestion to explore the adhesive performance on non-polar substrates, which is crucial for demonstrating the versatility and broad applicability of PU-P_xH_y adhesives. Here, we supplemented additional lap shear strength of PU-P_1H_3 adhesive bonded on polyethylene (PE), polypropylene (PP), and polytetrafluoroethylene (PTFE) surfaces (Fig. 3f). Encouragingly, the results indicate that PU-P_1H_3 adhesive also exhibits promising adhesive strength on these low surface energy materials, with 4.96 MPa on PP, 4.44 MPa on PP, and 2.25 MPa on PTFE. This can be primarily ascribed to the intimate contact between the adhesive and the substrate, which enables the formation of densely hydrogen-bonded MMSIs hard domains and PCL crystals, with both anchored at the interface. This not only significantly enhances interfacial adhesion but also contributes to a certain degree of mechanical interlocking. All the revisions are added in the revised manuscript (Page 16, line 358-360) and marked in blue for reading.

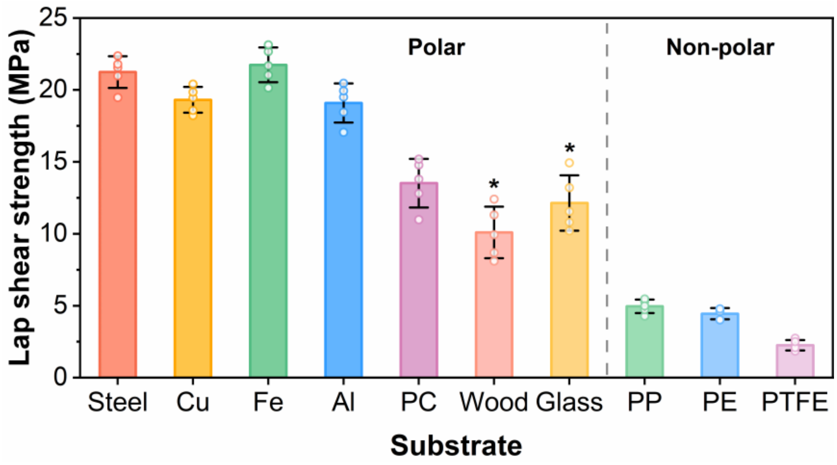

Fig. 3f | Lap shear strength of PU-P_1H_3 adhesive bonded on different substrates.

(8) What is the mass recovery efficiency of the adhesive following each recycling route (chemical dissolution and hot-press reprocessing)?

Response: Thank you for this valuable comment. We have supplemented the study with the required quantitative data.

For the hot-press reprocessing route, the theoretical mass recovery efficiency is 100% since no material is removed from the system during this physical process. We measured the mass of the sample before and after hot pressing and found that the mass loss was less than 0.5%, indicating almost complete recovery. Therefore, we reported a mass recovery efficiency of >99.5% for the hot-press reprocessing route.

For the chemical dissolution route, the process involves solvent transfer and evaporation, which can lead to minor operational losses. We measured the mass of the sample before and after chemical dissolution reprocessing and then reported a mass recovery efficiency of 98.4%.

All the revisions are added in the revised manuscript (Page 25, line 556, 559-560) and marked in blue for reading.

Responses to Reviewers' Comments

Reviewer #1: The authors have made considerable efforts to revise the manuscript and address the reviewers' comments. However, upon careful re-evaluation, several fundamental concerns remain inadequately resolved. These pertain to the conceptual novelty of the work, the rigor of the structural characterization, the directness of the evidence for interfacial interactions, and the reliability of the key mechanical performance data.

General Response: We sincerely appreciate the reviewer for reviewing the manuscript and providing insightful comments and constructive suggestions, which have significantly helped us improve the quality of our manuscript. All the remaining concerns have been carefully considered and addressed in the revised manuscript. A detailed point-to-point response to your valuable comments can be found below.

Major Concerns:

1. Incremental Novelty and Overlap with Recent Literature: In my initial assessment, I noted that the conceptual advance of this work appears incremental. The authors respond by framing their innovation as "the successful integration of known concepts into the thermosetting polyurethane systems." This argument, however, is substantially undermined by the existence of a highly relevant and recently published article in Nature Communications (Lu, G. et al. *Nat. Commun.* 2025, 16, 7754). Both studies address the central challenge of synergistically enhancing bulk cohesion and interfacial adhesion through multiscale structural design. To establish a distinct contribution, the authors must clearly articulate the essential scientific differentiation between their work and this published study. Claiming novelty based solely on a different material platform (thermoset vs. thermoplastic/supramolecular) is, in this context, insufficient.

Response: We sincerely appreciate the reviewer's insightful comments and the opportunity to further clarify the conceptual novelty of our work in relation to the study by Lu et al. (*Nat. Commun.* 2025, 16, 7754). Below we comprehensively address the concerns regarding innovation and differentiation by examining four key aspects: material platforms, scientific challenges, structural novelty, and fundamental differences in structural implementation.

1) Material platform and network design

As detailed in our initial response (Revision #1), the core innovation of our work lies in the fundamental creation of a new thermosetting network (i.e., dual-length dynamic crosslinks) to achieve on-demand integration of multiscale organizations, a capability previously precluded by covalent crosslinking constraints. This contrasts with the supramolecular system reported by Lu et al. where solution shear-induced alignment compensates for disordered self-assembly of multiscale structures. Our design represents a new network paradigm, distinct from external field-assisted approaches.

2) Scientific challenges and material limitations

The distinction manifests in addressing platform-specific constraints: the supramolecular system reported by Lu et al. requires solution shear-induced alignment to mitigate the intrinsic cohesion and adhesion deficiencies caused by the short chain nature of oligomers, whereas our thermoset system overcomes the inherent thermodynamic and kinetic conflict between high crosslink density (providing creep and thermal resistance) with sufficient structural freedom (enabling and integrating multiscale organizations) through the aforementioned network topology design, thereby transforming what was generally considered an irreconcilable trade-off in thermosetting polymers into synergistic coexistence without compensatory processing. This reflects fundamentally different challenges, optimizing pre-existing assembly in supramolecular system versus enabling previously impossible functionality in thermoset system.

3) Novelty beyond conventional multiscale structures

While structural features including hierarchical hydrogen bonds, crystalline domains, and other high-energy phases are conventional strategies for enhancing bulk cohesion and interfacial adhesion, their integration within a single system as demonstrated by both Lu et al. and our work represents a significant technical advancement, amplifying their adhesive enhancement effects. Crucially, however, it does not substantially diminish our contribution, as the primary innovation of each study stems from overcoming the material platform-specific barriers to their implementation. As demonstrated by Lu et al. with supramolecular oligomers possessing self-assembled multiscale structures, external solution-shearing is utilized to achieve oriented alignment of multiscale structures, thereby overcoming the intrinsic

performance limitations of oligomers. Similarly, in our work, the core innovation lies not in the employment of multiscale structures per se, but in overcoming the fundamental thermodynamic and kinetic barriers preventing their effective integration in crosslinked networks. Our approach establishes a new paradigm for thermosets through a key breakthrough: The development of dual-length crosslinked network that permit both high crosslink density and spontaneous integration of multiscale organizations, which is unachievable in conventional thermosetting materials.

4) Fundamental differences in structural control

The fundamental differences between the work of Lu et al. and ours manifests most profoundly in how multiscale structures are realized. The Supramolecular system reported by Lu et al. requires directional shear fields to align pre-assembled structures, yielding process-dependent anisotropic properties. In contrast, our thermosetting system achieves spontaneous isotropic organizations through network-guided structural control, eliminating orientation-dependent performance and enabling simplified fabrication. Moreover, the dual-length dynamic crosslinked network serves as the macroscale structure, which not only constitutes the core for achieving top-down multiscale structures integration but also represents a crucial component distinguishing our multiscale structures from that reported by Lu et al. This distinction between externally assisted and intrinsically encoded structural control expands the design spectrum for advanced adhesives.

Collectively, these analyses demonstrate that while both studies employ multiscale structures designs, the fundamental innovations lie in their respective solutions to material-specific challenges: Lu et al.'s solution shear-mediated alignment overcomes the intrinsic limitations of supramolecular oligomers, whereas our network reengineering transcends traditional thermoset constraints. This work thus establishes a new crosslinked networks paradigm for thermosetting adhesives, achieving previously inaccessible integration of multiscale structures and thereby synergistically enhancing bulk cohesion and interfacial adhesion.

All the revisions are added in the revised manuscript (Page 1, line 22-24; Page 4, line 77-79, and 93-95) and marked in blue for reading.

2. Ambiguity in Structural Differentiation: A critical mechanistic question remains unaddressed:

Does the crystallization of the PCL chains influence the microphase separation scale or the ordering of the MMSIs hard domains within the original PU-P_1H_3 sample? The potential interplay between these two structural elements requires explicit investigation to validate the proposed decoupled contributions to bulk cohesion and interfacial adhesion.

Response: Thank you for your insightful comment regarding the potential interplay between PCL crystals and MMSIs hard domains. Below, we address this concern in two parts:

1) Theoretical and experimental evidence of structural interplay

We fully acknowledge that PCL crystals and MMSIs hard domains may thermodynamically and kinetically influence each other, as is well-established for competing ordering processes in multiblock copolymers (*The Physics of Block Copolymers*, Oxford University Press, 1998; *Principles of Polymer Chemistry*, Cornell University Press, 1953; *Prog. Polym. Sci.* 2009, 34, 1161-1210). Specifically, PCL crystallization inevitably restricts chain mobility, which could perturb MMSIs microphase separation by reducing the effective χ parameter or slowing domain ordering kinetics. However, our multiple characterization techniques in original manuscript (e.g. AFM, POM, WAXS, and SAXS) confirm that both PCL crystals and MMSIs hard domains coexist in the PU-P_xH_y sample, despite their competitive formation kinetics. While the degree of ordering (e.g., crystallinity or domain size) may be slightly compromised compared to systems with only one ordered structure, the coexistence of both phases has been proven to be optimal for adhesion performance (Revision #1) since their primary contributions remain distinct. More importantly, we would like to emphasize that the mutual influence between PCL crystals and MMSIs hard domains during the formation process does not preclude their contributions as two distinct organizations to the adhesive performance. The above shows that competing ordered structures can synergize in PU-P_xH_y adhesives, even if their formation pathways interfere.

2) Control experiments decouple functional contributions

The concern about structural interplay does not invalidate our conclusion of the proposed decoupled contributions to bulk cohesion and interfacial adhesion because the control experiments (Revision #1. Page 22-23, line 497-531 in the manuscript of revision #2) have explicitly isolated the functional roles of PCL crystals and MMSIs hard domains. Specifically, to elucidate their respective contributions to interfacial adhesion, two additional prepolymers

were synthesized for decoupled comparison (Fig. 5d, in the manuscript of revision #2). Compared with PU-P_1H_3, replacing the soft segment with non-crystallizable polypropylene glycol (PPG) yielded PU-$P1_1H_3$ containing only MMSIs hard domains, while removing the MMSIs units produced PU-$P2_1H_3$ retaining solely PCL crystals. The comparative results of Peak Force Quantitative Nanomechanical Mapping (PF QNM) and lap shear tests among the three groups provide clear evidence for the decoupled contributions of PCL crystals and MMSIs hard domains to interfacial adhesion (Fig. 5e, f, and Supplementary Fig. 26, in the manuscript of revision #2): i) PU-$P2_1H_3$ exhibited the highest average surface modulus, PU-$P1_1H_3$ the lowest, and PU-P_1H_3 showed an intermediate value with a broader distribution, confirming the coexistence of these two competing high-energy phases on the PU-P_1H_3 surface. ii) PU-$P1_1H_3$ demonstrated the highest mean interfacial adhesion forces, while PU-$P2_1H_3$ displayed the lowest, indicating that MMSIs hard domains dominate interfacial adhesion enhancement, whereas PCL crystals play a weaker yet non-negligible role. iii) PU-P_1H_3, incorporating both MMSIs hard domains and PCL crystals, achieved the highest adhesive strength and work of debonding. In comparison, the absence of PCL crystals in PU-$P1_1H_3$ led to a sizeable decrease in bulk cohesion, resulting in a reduction of over 50% in both adhesive strength and debonding work, thereby demonstrating that the primary contribution of PCL crystals is enhancing bulk cohesion.

Finally, while we acknowledge that a detailed quantitative investigation into the potential interplay between PCL crystals and MMSIs hard domains in PU-P_xH_y during the formation process could constitute an interesting independent study, we respectfully submit that such research would not substantially alter the core conclusions and key contributions of this work. Our study focuses on establishing a novel dual-length dynamic crosslinked network paradigm that enables on-demand integration of multiscale organizations to synergistically enhance both bulk cohesion and interfacial adhesion of the adhesive. Regardless of the specific quantitative relationship between PCL crystals and MMSIs hard domains during the ordering process, the proposed decoupled contributions demonstrated by our control experiments remains valid.

3. Lack of Direct Chemical Evidence for Interfacial Bonding: The authors have supplemented their manuscript with DFT simulations and AFM adhesion force mapping. While these

techniques provide useful indirect probes, they do not constitute direct chemical evidence for the proposed interfacial interactions (e.g., hydrogen bonding, cation-π interaction, metal-ion multiple oxygen binding). More definitive spectroscopic evidence at the interface is necessary to substantiate these claims.

Response: Thank you for your insightful comment regarding the direct chemical evidence for interfacial bonding.

We acknowledge that direct spectroscopic characterization of buried interfaces in adhesive joints is inherently challenging due to the inaccessibility of the interface. Once the adhesive bonds to the substrate, the interface is physically buried, making traditional spectroscopic techniques (e.g., FTIR, Raman, XPS) difficult to apply in situ.

Nevertheless, we have made significant efforts to provide direct chemical evidence supporting the proposed interfacial interactions. To probe the interfacial interactions, we conducted FTIR and Raman comparative analyses between the bulk adhesive and the adhesive-side surface of the interfacial failure joints (where the adhesive detached from the stainless steel substrate). Notably, even after interfacial failure of the adhesive joints, the transferred interfacial species (e.g., single atomic layers of Fe) are present at trace levels and non-uniformly distributed. Therefore, the related signal sensitivity of these spectra is low. As shown in Supplementary Fig. 24, FTIR spectra revealed a redshift in the N-H stretching vibration peak from 3369.0 cm^{-1} (adhesive) to 3365.2 cm^{-1} (adhesive interface), indicating hydrogen bond formation at the interface. Similarly, the C=C stretching vibration peak of benzene ring shifted from 1515.8 cm^{-1} to 1513.8 cm^{-1}, supporting the presence of cation-π interactions. As shown in Supplementary Fig. 25, Raman spectroscopy further confirmed interfacial bonding, showing a redshift in the C=O stretching peak (1768.7 cm^{-1} → 1765.7 cm^{-1}) and the emergence of a new peak at 515.6 cm^{-1}, assigned to Fe-O coordination. While these spectral shifts are subtle, as expected for interfacial interactions involving trace amounts of transferred species, they are nevertheless reproducible and show excellent agreement with our DFT predictions. This consistency provides compelling evidence for the presence of hydrogen bonding, cation-π interaction, and metal-ion multiple oxygen binding at the adhesive interface.

All the revisions are added in the revised manuscript (Page 22, line 491-497) and marked in blue for reading.

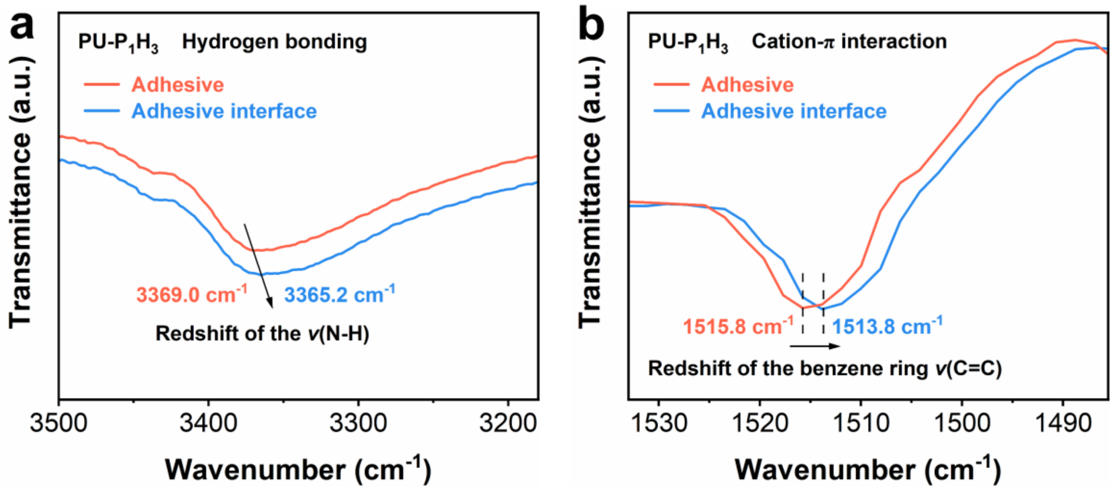

Supplementary Fig. 24 | FTIR spectra of PU-P$_1$H$_3$ before and after interfacial failure with stainless steel substrate. a N-H stretching vibration, **b** C=C stretching vibration of benzene ring.

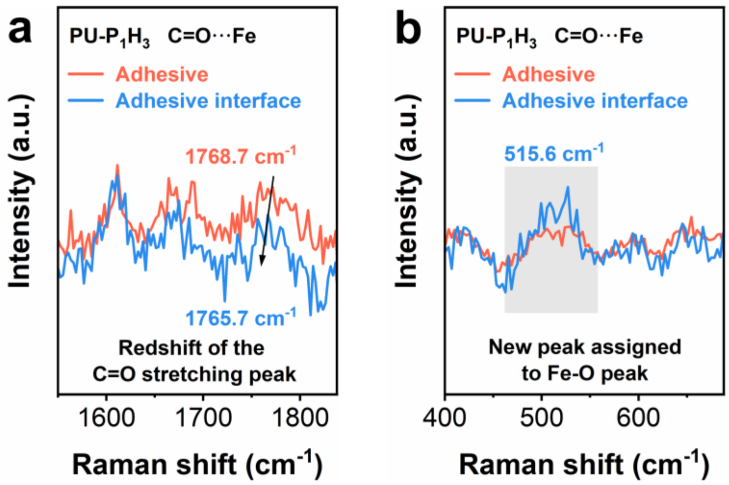

Supplementary Fig. 25 | Raman spectra of PU-P$_1$H$_3$ before and after interfacial failure with stainless steel substrate. a C=O stretching peak, **b** new Fe-O peak formed at the adhesive interface.

4. Insufficient Experimental Details for Key Mechanical Tests: The inclusion of interfacial fracture toughness and fatigue threshold data is a positive addition. However, the experimental details provided are insufficient to assess the reliability of these critical measurements. A comprehensive description of the testing protocols is essential for the data to be properly evaluated.

Response: We sincerely appreciate the reviewer's constructive comments regarding the experimental details. As suggested, we have expanded the Methods section to include the

following additional details.

The 90° peeling test was conducted to evaluate the interfacial fracture toughness of PU-P_1H_3 adhesive to stainless steel substrates (ASTM D2861). The adhesive, thickness-controlled by a mold, was sandwiched between a plasma-treated PET backing and a stainless steel substrate for hot-pressing bonding. During testing, the sample (110 × 20 × 0.5 mm) was mounted on a 90° peel fixture, with the stainless steel substrate fixed horizontally and the PET backing clamped to the force sensor of UTM2203 instrument (SUNS, China). The backing was peeled at a constant 90° angle (50 mm min^{-1}) via a pulley system. Interfacial fracture toughness was calculated as the average steady-state peel force (F_s) divided by the specimen width.

The cyclic 90° peeling test was conducted to characterize the interfacial fatigue threshold of PU-P_1H_3 adhesive to stainless steel substrates. The methodology involved applying cyclic peeling forces F_a ($F_a < F_s$) over N cycles while monitoring the interfacial crack extension (c) as a function of cycle number N. Accordingly, the applied energy release rate G can be calculated as the F_a divided by the specimen width, and the interfacial crack propagation rate as dc/dN. Through systematic variation of applied force amplitudes, the characteristic dc/dN-G relationship was established, from which Γ_0 was extrapolated as the G-axis intercept of the linear regression.

All the revisions are added in the revised manuscript (Page 35-36, line 793-808) and marked in blue for reading.

Reviewer #2: The authors have significantly improved the manuscript and provided substantial new data. However, a few critical points regarding the underlying mechanism and terminology require further clarification.

General Response: We sincerely thank the reviewers for acknowledging our efforts in improving the manuscript. All critical points raised regarding the underlying mechanism and terminology have been considered and addressed in the revised manuscript. A detailed point-to-point response to your valuable comments can be found below.

1. Clarification of the Bulk-Interface Relationship

A logical gap persists in the interpretation of the adhesion mechanism. Previous results indicated that the lap shear strength correlates strongly with the bulk tensile strength. However, the optical images show a clean interfacial failure (Supplementary Fig. 25), which typically indicates that the interfacial interaction is the performance-limiting factor. Furthermore, Fig. 5e shows that the adhesion force of PU-P_1H_3 is only moderate. These observations strongly suggest that interfacial adhesion chemistry may not be the dominant factor driving the macroscopic performance. The authors should clarify which factor is the ultimate determinant of the lap shear limit in this system: the bulk yield strength or the interfacial bonding strength? A more systematic discussion on this relationship would significantly improve the scientific impact of the work.

Response: We are grateful to the reviewer's insightful comments regarding the adhesion mechanism. Here we clarify that the lap shear strength in our system is governed primarily by bulk properties rather than interfacial interaction, despite the apparent interfacial failure mode.

As noted in your comment, our experimental data confirm a strong correlation between lap shear strength and bulk tensile strength, indicating that the bulk mechanical strength governs the practical adhesion limit. Regarding the observed interfacial failure of PU-P_1H_3, it does not indicate weak interfacial bonding, but rather stems from sufficiently enhanced bulk strength and strain-induced crystallization (SIC)-modified stress distribution. We term this specific scenario "forced interfacial failure".

Specifically, as the proportion of crosslinked short chains (y) in PU-P_xH_y increases, its tensile strength and bulk cohesion improve. Conversely, due to the enhanced modulus, reduced

wettability, and decreased mass fraction of MMSIs units, the interfacial interactions actually exhibit a declining trend. The lap shear strength closely aligns with the trend of tensile strength, indicating that the lap shear limit is governed by bulk cohesion. Furthermore, interfacial adhesion is primarily provided by molecular interactions, while the modulus (stiffness) of the adhesive directly influences stress transfer efficiency. A higher modulus of adhesive leads to reduced deformation under load, resulting in more significant interfacial stress concentration. If interfacial adhesion were the dominant factor, changes in modulus should have a more pronounced impact on lap shear strength. However, the opposite is observed, further supporting the dominance of bulk strength. Regarding the observed interfacial failure in PU-P_1H_3, we propose the following mechanistic interpretation. The lap shear strength of PU-P_xH_y corresponds to the tensile stress within the 200-300% strain range (Fig. 2a, in the original manuscript), where the stress response is governed by the synergistic interplay between intrinsic network strength and SIC. And the SIC process has been effectively demonstrated by in situ tensile wide-angle X-ray scattering (WAXS) and its crystallinity calculation (Fig. 2g and Supplementary Table 5, in the original manuscript and supplementary information). The counterintuitive interfacial failure phenomenon is documented and systematically researched in polyurethane adhesives (e.g., *Mater. Horiz.* 2023, 10, 4183-4191): although the initial interfacial bonding exceeds the bulk strength (nominally favoring cohesive failure), the dynamic modulus enhancement via SIC during shear deformation elevates the bulk strength above the interfacial threshold, resulting in an anomalous interfacial failure that contradicts classical adhesion paradigms. In our work, this was further proven by the control group (PU-P1_1H_3) using non-crystallizable soft segments (PPG), which exhibited improved interfacial adhesion force (Fig. 5e, in the original manuscript) and reduced bulk strength (Supplementary Fig. 27), showing a mixed failure mode (Supplementary Fig. 28, in the revised supplementary information). This reveals two key findings: (1) When the ratio of long to short crosslinked chain reaches 1:3, the bulk cohesion of PU-P_1H_3 may match or even exceed interfacial interactions; (2) SIC promotes bulk self-reinforcement, surpassing interfacial bonding and leading to stress concentration and failure at the interface. Thus, while interfacial chemistry remains important, the ultimate lap shear limit is determined by the bulk strength, with SIC kinetically controlling the final failure mode. This mechanistic framework aligns with

established literature while providing new insights into how crystallizable networks achieve strong yet cleanly debonding adhesives.

All the revisions are added in the revised manuscript (Page 23, line 517-529) and marked in blue for reading.

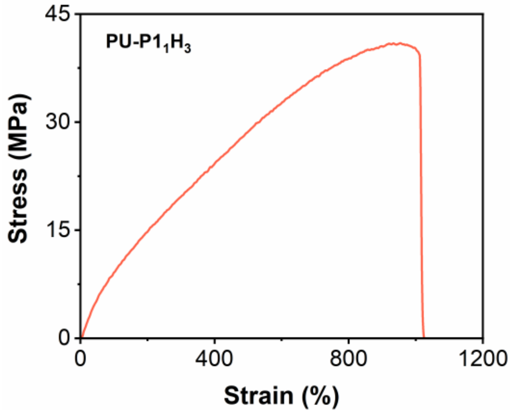

Supplementary Fig. 27 | Stress-strain curve of PU-P1$_1$H$_3$.

2. Additional Data for PPG Control

While surface adhesion data for the PPG-based adhesive were provided, please supplement its bulk tensile stress-strain curves as well. This is necessary to determine if its mixed failure mode stems from interfacial effects or simply a consequence of reduced bulk cohesive strength.

Response: We sincerely appreciate the reviewer's valuable suggestion. The requested bulk tensile stress-strain curve of the PPG-based adhesive (PU-P1$_1$H$_3$) have been supplemented as follows (Supplementary Fig. 27).

The tensile stress-strain curve of PU-P1$_1$H$_3$ provide critical insights into the failure mechanism transition. Overall, compared to PU-P$_1$H$_3$, PU-P1$_1$H$_3$ exhibited improved interfacial adhesion force (Fig. 5e, in the original manuscript) and significant reduced bulk strength (Supplementary Fig. 27). At this stage, the bulk cohesion matches the interfacial interaction, causing the crack to propagate along a stochastic path. This indicates that its mixed failure mode results from the combined effects of interfacial interaction and bulk cohesion, with the influence of bulk strength reduction playing a dominant role. More importantly, the elimination of strain-induced crystallization (SIC) fundamentally alters the stress distribution behavior. Without SIC-mediated dynamic self-reinforcing, the system achieves more uniform stress distribution, allowing simultaneous activation of both interfacial debonding and bulk plastic deformation.

This contrasts sharply with the PU-P_1H_3 where SIC forces stress concentration at the interface, causing preferential debonding despite strong intrinsic adhesion.

All the revisions are added in the revised manuscript (Page 22, line 514) and marked in blue for reading.

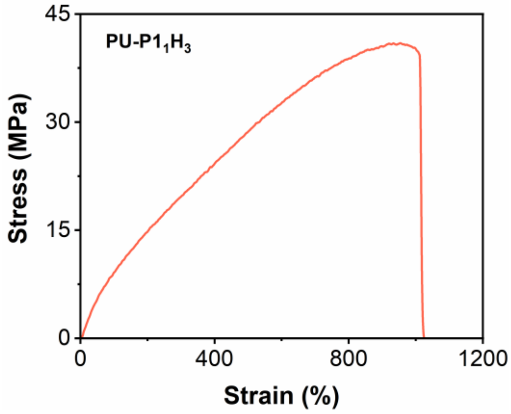

Supplementary Fig. 27 | Stress-strain curve of PU-$P1_1H_3$.

3. Terminology: "Top-Down" Design

In materials science, “top-down” (e.g., lithography) and “bottom-up” (e.g., molecular synthesis) have well-established, distinct definitions. The authors are encouraged to re-examine these standard definitions and reconsider if “top-down” accurately describes a molecular-scale synthesis and assembly process.

Response: We appreciate the reviewer’s suggestion for clearer terminology. To avoid potential confusion with conventional definitions of “top-down” (e.g., lithography), we have removed the term “top-down” from expressions such as “this top-down design” and “top-down multiscale structures” throughout the manuscript, retaining only one instance of “top-down design philosophy” to preserve the original conceptual framework. This adjustment better reflects our strategy of initiating from macroscale crosslinked network design and proceeding to molecular scale optimization, while maintaining consistency with materials science terminology.

All the revisions are added in the revised manuscript (Page 1, line 1; Page 3, line 76; Page 5, line 115; Page 6, line 133-134; Page 15, line 342; Page 21, line 467; Page 29, line 634-635, and line 649) and marked in blue for reading.

Reviewer #3: The previous comments have been addressed.

General Response: We are grateful for your recognition of our work and the decision to accept our paper for publication. Thank you for your time and constructive feedback throughout the review process.

Responses to Reviewers' Comments

Reviewer #1: The authors have made substantial efforts to revise the manuscript and address previous concerns, and the overall quality of the work has been significantly improved. However, before final acceptance, I would like to offer a few final suggestions to further strengthen the contextualization and completeness of the manuscript.

General Response: We sincerely thank the reviewer for acknowledging our efforts in improving the manuscript. All critical points concerning the novelty discussion and important literature citations have been considered and addressed in the revised manuscript. A detailed point-to-point response to your valuable comments can be found below.

1. Differentiation from a closely related work in Introduction

In the rebuttal letter, the authors provided a detailed point-by-point differentiation between their work and a recently published Nature Communications article by Lu et al. (Nat. Commun. 2025, 16, 7754). To better guide readers and clearly establish the conceptual novelty of the present study, I strongly recommend that the authors incorporate this critical comparison directly into the Introduction section of the main manuscript. Specifically, the authors should highlight how their thermosetting dual-length dynamic crosslinked network approach fundamentally differs from the solution shear-induced alignment strategy used in Lu et al.' s supramolecular oligomer system, particularly regarding how each platform addresses the central challenge of simultaneously enhancing interfacial adhesion and bulk cohesion.

Response: We sincerely appreciate the reviewer's constructive suggestion. We have incorporated the critical comparison with the recently published article by Lu et al. (*Nat. Commun.* 2025, 16, 7754) directly into the Introduction section of the main manuscript. Specifically, we have highlighted that Lu et al. overcame the intrinsic limitations of supramolecular oligomers by using a solution shear-induced alignment strategy to form oriented multiscale structures. In contrast, our work establishes a new crosslinked networks paradigm (dual-length crosslinked network) to overcome the fundamental thermodynamic and kinetic barriers that preventing effective integration of multiscale structures in crosslinked networks, thereby synergistically enhancing bulk cohesion and interfacial adhesion.

All the revisions are added in the revised manuscript (Page 3, line 55-69, line 73-75) and marked in blue for reading.

2. Missing important references in the second paragraph of Introduction

In the second paragraph of the Introduction, when discussing molecular design strategies for simultaneously improving the strength and toughness of adhesives, several important recent contributions have been overlooked. The authors should cite the following relevant works to provide a more comprehensive literature background:

Science Advances 2025, 11, eaea3097

Advanced Functional Materials 2023, 33, 2306914

ACS Applied Materials & Interfaces 2023, 15, 44194-44204

These studies present complementary approaches to addressing the strength-toughness dilemma in polymeric adhesives and deserve appropriate acknowledgment in the revised manuscript.

Response: We sincerely appreciate the reviewer for the valuable suggestion to include additional recent and relevant references. As suggested, we have cited the recommended literature in the second paragraph of Introduction of revised manuscript (Page 3, line 59), and the changes have been marked in blue for reading.

Given the overall novelty of the multiscale design concept, the impressive performance metrics, and the comprehensive set of characterization data provided, the manuscript is suitable for publication pending the incorporation of the above minor revisions.

Response: We are grateful for your insightful suggestions and recognition of our work. Thank you for your time and constructive feedback throughout the review process.

Reviewer #2: The revised manuscript can be accepted.

General Response: We are grateful for your positive evaluation and the acceptance of our paper for publication. Thank you for your time and constructive feedback throughout the review process.
